# On Regularized Systems of Equations for Gas Mixture Dynamics with New Regularizing Velocities and Diffusion Fluxes

**DOI:** 10.3390/e25010158

**Published:** 2023-01-12

**Authors:** Alexander Zlotnik, Timofey Lomonosov

**Affiliations:** 1 Department of Mathematics, Faculty of Economic Sciences, Higher School of Economics University, Pokrovskii Bd. 11, Moscow 109028, Russia; 2 Keldysh Institute of Applied Mathematics, Miusskaya Sqr., 4, Moscow 125047, Russia

**Keywords:** regularized equations for one-velocity and one-temperature gas mixture dynamics, entropy balance equation, linearization, three-point symmetric spatial discretization, discrete entropy balance equation, 35K40, 65M06, 76N99, 76T99

## Abstract

We deal with multidimensional regularized systems of equations for the one-velocity and one-temperature inert gas mixture dynamics consisting of the balance equations for the mass of components and the momentum and total energy of the mixture, with diffusion fluxes between the components as well as the viscosity and heat conductivity terms. The regularizations are kinetically motivated and aimed at constructing conditionally stable symmetric in space discretizations without limiters. We consider a new combined form of regularizing velocities containing the total pressure of the mixture. To confirm the physical correctness of the regularized systems, we derive the balance equation for the mixture entropy with the non-negative entropy production, under generalized assumptions on the diffusion fluxes. To confirm nice regularizing properties, we derive the systems of equations linearized at constant solutions and provide the existence, uniqueness and *L*^2^-dissipativity of weak solutions to an initial-boundary problem for them. For the original systems, we also discuss the related Petrovskii parabolicity property and its important corollaries. In addition, in the one-dimensional case, we also present the special three-point and symmetric finite-difference discretization in space of the regularized systems and prove that it inherits the entropy correctness property. We also give results of numerical experiments confirming that the discretization is able to simulate well various dynamic problems of contact between two different gases.

## 1. Introduction

Multicomponent compressible gas mixture dynamics is an important field in science and engineering, and a number of systems of partial differential equations (PDEs) were developed to describe phenomena of such type, see, in particular, references [1,2,3] and references therein.

Numerical methods serve as the most powerful tool to solve and simulate such systems of quasilinear PDEs. Originally, various numerical methods were designed to solve the compressible single-component gas dynamics systems of PDEs, and vast literature is devoted to this subject, see, in particular, references [4,5,6] and references therein.

Preliminary regularization of equations is an important and frequently used approach in constructing numerical methods for solving various scientific problems. In computational physics, those regularizations that have a physical basis are usually preferred. Such numerical methods are also used in computational gas dynamics. These include explicit in time conditionally stable and symmetric in space finite-difference and finite volume methods without limiters based on the discretization of regularized, or the so-called quasi-gas-dynamic (QGD), equations of gas dynamics. It is well known that, without regularization, methods of such type are unstable. These QGD equations were originally constructed on the basis of the Bhatnagar–Gross–Krook model kinetic equations, see monographs [7,8,9]. They can be rewritten in the form akin to compressible Navier–Stokes–Fourier equations with artificial coefficients of viscosity and heat conductivity and additional second order terms in space representing the regularizing velocity, viscous stress and heat flux, with a small parameter τ>0. These equations can also be obtained on the basis of compressible Navier–Stokes–Fourier equations using formal procedures of time averaging and expansion [10,11,12]. Numerical methods based on the QGD equations have been successfully tested in practice for almost 40 years, including complex applied problems; see an extensive bibliography in the above monographs and numerous subsequent works, among which we highlight only a few works devoted to 3D turbulence and magnetohydrodynamics problems and the inclusion of such methods in the well known open source software package OpenFOAM [13,14,15,16]. Note that τ is taken proportional to the characteristic spatial mesh step in numerical methods. The QGD equations were proved to be physically correct, in the sense that they imply the correct entropy balance equation, i.e., with a non-negative entropy production. Some mathematical regularizing properties of the QGD equations were also confirmed, including their Petrovskii parabolicity, in contrast to the Euler equations and compressible Navier–Stokes–Fourier equations, which have hyperbolic and composite hyperbolic–parabolic types, respectively, as well as $L^{2}$-dissipativity of the QGD equations linearized on constant and equilibrium solutions [17,18]. Recall that the important question of correct setting of boundary conditions is closely related to the type of a system of PDEs, and this setting is usually the most complicated in the hyperbolic case and the simplest in the parabolic case. In addition, conditional stability theorems were proved in the linearized statement for the above mentioned difference methods based on the QGD equations [19,20,21]. Notice also a quasi-hydrodynamic (QHD) regularization which can be considered as a simplification of the QGD one applicable to some subsonic or transonic flows [8,9].

There are other regularizations of the gas dynamics equations, which were also studied mathematically and aimed at constructing new numerical methods; in particular, see three approaches [22,23,24,25] and [26,27]. In all the approaches, much attention is paid to the entropy correctness of regularized equations. Among the listed approaches, the last one based on the so-called bi-velocity hydrodynamics [28,29], is closest to the QGD approach in structure of equations, although they are far from being the same. Alternative approaches also demonstrate success, but so far they have not yet undergone such extensive multi-year testing as the QGD approach.

This paper is related to further development of the QGD and QHD regularizations and the corresponding numerical methods in the case of multicomponent gas mixture dynamics, and we deal with the so-called one-velocity and one-temperature homogeneous gas mixture model, with the perfect polytropic components. The reason is that such type models are widespread in practice including the computational design of aircraft and rocket engines.

For binary mixtures, the original regularized QGD multi-velocity and multi-temperature homogeneous gas mixture model was constructed on the basis of kinetic equations for mixtures in ([8], Chapter 9). It was rewritten in [30] in the form of the compressible Navier–Stokes–Fourier equations with exchange terms for components and additional regularizing velocities, viscous stresses and heat fluxes, and a justification of its entropy correctness was given. Concerning applications, in particular, see [8,31]. For multicomponent mixtures, see similar QGD model and its applications in [32,33]. We do not touch this model here. The transition to the QGD one-velocity and one-temperature model was accomplished in ([34], Section 1) by aggregating the PDEs of the original model. The aggregation procedure is simple and consists in using the balance PDEs for the mass of components and the momentum and total energy of the mixture, followed by taking the common velocities and temperatures of the components in them. The main advantage of this procedure is that the entropy correctness of the resulting QGD model is guaranteed. A mathematical analysis of such a multicomponent QGD regularization, as well as its QHD simplification, with additional allowance for diffusion fluxes between components, has recently been given in [35].

In [36], another approach to the construction of regularized QHD Navier–Stokes–Fourier–Cahn–Hilliard equations at low Mach numbers was given, based on the well-known Coleman–Noll procedure and also ensuring the entropy correctness of the QHD model. The regularizing velocity $w_{\alpha }$ of the component α, which play an important role in the QGD and QHD regularizations, turned out to be different in [34,36] depending on the partial pressure $p_{\alpha }$ and the total pressure *p*, respectively. An additional full or partial averaging of $w_{\alpha }$ from [34] can be applied (for the QHD regularization, they are the same), which also makes the result depending on *p*, see the next Section. A mathematical analysis of such a multicomponent QHD regularization has recently been given in [37]; among other things, it turned out that, in contrast to the single-component case, in the absence of diffusion fluxes, the QHD system of PDEs acquires the composite hyperbolic–parabolic type, i.e., the regularization becomes incomplete. Theoretical constructions were accompanied by experiments with corresponding difference schemes, see [34,38,39,40,41], etc. The full averaging of $w_{\alpha }$ seems to be unsuccessful since the entropy correctness of the QGD regularization with it was established in ([34], Section 2) only by passing to a non-conservative modification of the balance PDE for the total energy of the mixture, and the corresponding difference schemes gave satisfactory results only in simple 1D tests, in particular, see Section 5 below.

The partial averaging of $w_{\alpha }$ corresponds to the combined $w_{\alpha }$, and the first sufficiently successful experiments with corresponding difference schemes are presented in [42]. An incomplete attempt to derive the combined $w_{\alpha }$ and the entire regularized system using the approach from [10] is also made there. In this case, the regularizing viscous stress tensor and heat flux turn out to be different than in ([34], Section 1) in contrast to the single-component case, and a significant drawback of such a system in [42] is the loss of entropy correctness.

In this paper, we analyze the effect of these new combined regularizing velocities of components $w_{\alpha }$ depending on the densities of the components and the total pressure. However, we still apply the same aggregated regularized balance PDEs for the momentum and total energy of the mixture as in ([34], Section 1.2) and [35] in the case of binary and general multicomponent mixtures, respectively. In addition, we involve a new generalized form of the diffusion fluxes between the mixture components. Following [35], we study both the QGD and QHD regularizations in a unified manner by introducing a parameter in the corresponding PDEs. The first main theoretical result of the paper is the derivation of the balance equation for the mixture entropy with non-negative entropy production for our essentially modified system of equations. The second result concerns the derivation and study of the linearized system of PDEs: we justify the existence, uniqueness and $L^{2}$-dissipativity of weak solutions to an initial-boundary value problem for this system. We also discuss that our results imply the Petrovskii parabolicity of the original quasilinear system of PDEs which allows one to obtain the local-in-time classical unique solvability of the Cauchy problem for this system identical to ([35], Theorem 3.3) and simplify the statement of correct boundary conditions for it. We emphasize that the presence of the diffusion fluxes is crucial for validity of the second and related results, for, without them, the original system of PDEs becomes a more complicated composite hyperbolic–parabolic, as in [37], not parabolic. This discovered regularizing role of the diffusion fluxes is nontrivial and even somewhat surprising. Notice that important mathematical results on the properties of other PDEs for compressible heat-conducting gas mixtures were proved, in particular, in [2,43,44,45,46].

In the one-dimensional (1D) case, we also consider the new special three-point and symmetric finite-difference discretization which modifies one suggested in [41] in the case of the new regularizing velocities and more general form of the diffusion fluxes; for the single-component gas dynamics PDEs, this discretization was suggested, generalized and computationally tested in [47,48,49]. The discretization uses some non-standard nonlinear averages of the densities of the components and temperature and is conservative in the mass of the components and the momentum and total energy of the mixture. Our main theoretical result relating to this discretization is the semi-discrete balance equation for the mixture entropy with the non-negative entropy production; it means that the constructed discretization is entropy correct. In addition, results of our numerical experiments demonstrate better (sometimes, much better) or not worse behaviour, depending on the test, for the new combined regularizing velocities compared to those used previously. Now we can hope that the entropy correct discretizations of the considered type will be further developed for the general multidimensional gas mixture dynamics PDEs in the spirit of [48].

Vast literature is devoted to other numerical methods for solving multicomponent gas dynamics PDEs. We refer the reader to the brief review and a collection of references in the recent paper [50]. Note that only a few of the papers touch the entropy correct methods [51]. This is an important but complicated subject even in the case of single-component gas dynamics PDEs, see, in particular, reviews: Tadmor, E., Entropy stable schemes ([6], Chapter 17) and Carpenter, M.H.; Fisher, T.C.; Nielsen, E.J. et al. Entropy stable summation-by-parts formulations for compressible computational fluid dynamics ([6], Chapter 19) and references therein.

The structure and the results of the paper in more detail are as follows. In Section 2, we present the aggregated regularized systems of PDEs describing the multidimensional one-velocity and one-temperature homogeneous gas mixture model, define the collection of all the involved functions and pass to the combined regularizing velocities. Proposition 1 concerns properties of the average gas mixture parameters, and Proposition 2 establishes a useful particular connection between solutions to the regularized systems of PDEs for the gas mixture dynamics and single-component gas dynamics. The main result is Theorem 1 about the balance equation for the mixture entropy with the non-negative entropy production. In Section 3, we derive and study the linearized system of PDEs. The key role belongs to the properties of symmetry/skew symmetry and positive definiteness of the related bilinear forms considered in Lemma 2. Theorem 2 states the existence, uniqueness and $L^{2}$-dissipativity of weak solutions to an initial-boundary value problem for the linearized system. We also discuss the Petrovskii parabolicity of the original quasilinear system of PDEs and a local-in-time classical unique solvability of the Cauchy problem for this system. In Section 4, we pass to the 1D case of the regularized system of PDEs, introduce the mesh notation and present a special three-point and symmetric discretization in space for 1D regularized systems. Theorem 3 contains a semi-discrete balance equation for the entropy of the gas mixture, with a non-negative entropy production, and serves as a counterpart of Theorem 1. Section 5 is devoted to 1D numerical experiments. Applying the constructed discretization, we solve four known tests from [52,53,54,55]. The results confirm that the discretization is able to simulate well various dynamic problems of contact between two different gases, including the case of high initial pressure drops, and have some advantages over other choices of the regularizing velocities from [34] and especially from [37,39].

The paper also contains four appendices. Appendix A is devoted to derivation of the combined regularizing velocities and the full regularized system of PDEs from [39] based on the Euler-type system of PDEs for multicomponent gas mixture dynamics, by applying a formal procedure suggested in [11]. In Appendix B, we accomplish the scaling of the regularized system of PDEs from Section 2 that is often used to solve practical problems. In Appendix C, for the 1D case of the Euler-type system of PDEs from Appendix A, the Rankine–Hugoniot relations on the shock wave are given, and conditions for the existence of a stationary shock wave and the relationship between the values of the sought functions to the left and right of it are derived. Finally, in Appendix D, the 1D finite-difference counterpart of Proposition 2 is given.

## 2. A Regularized System of Equations for the Multicomponent Gas Mixture Dynamics with New Regularizing Velocities in the Presence of Diffusion Fluxes

The aggregated regularized system of PDEs for one-velocity and one-temperature multicomponent homogeneous gas mixture dynamics consists of the following balance PDEs for the mass of components, total momentum and total energy of the mixture:

(1) $$\begin{array}{c} \partial_{t}\rho_{\alpha }+\mathrm{div}\left(\rho_{\alpha }\left(u-w_{\ell \alpha }\right)+d_{\alpha }\right)=0,\;\;\alpha =\bar{1,K}, \end{array}$$

(2) $$\begin{array}{c} \partial_{t}\left(\rho u\right)+\mathrm{div}\left(\rho (u-w_{\ell })⊗u\right)+\nabla p=\mathrm{div}\Pi_{\ell }+\left(\rho -\ell \tau \mathrm{div}(\rho u)\right)f, \end{array}$$

(3) $$\begin{array}{c} \;\partial_{t}E+\mathrm{div}\left({0.5\rho |u|}^{2}\left(u-w_{\ell }\right)+\langle \rho_{\alpha }h_{\alpha }\left(u-w_{\ell \alpha }\right)\rangle \right)=\mathrm{div}\left(-q+\Pi_{\ell }u\right)+\rho \left(u-w_{\ell }\right)\cdot f+Q. \end{array}$$

Here, the main sought functions are the densities of the mixture components $\rho_{1}>0,\ldots ,\rho_{K}>0$ (K⩾2 is their amount), their common velocity $u=(u_{1},\ldots ,u_{n})$ and absolute temperature θ>0. These functions depend on $x=(x_{1},\ldots ,x_{n})\in \Omega$ and t⩾0, where Ω is a domain in $R^{n}$, n=1,2,3, and $\alpha =\bar{1,K}$ means that α=1,…,K. Vector-functions are written in bold. The operators div=∇· and $\nabla =(\partial_{1},\ldots ,\partial_{n})$ are taken in *x*, $\partial_{t}=\frac{\partial }{\partial t}$ and $\partial_{i}=\frac{\partial }{\partial x_{i}}$. In this section, the symbols ⊗ and · denote the tensor and scalar products of vectors, the tensor divergence is taken with respect to its first index, and 〈·〉 is the operation of summation over index $\alpha =\bar{1,K}$.

This regularized system of PDEs was derived in [34] for K=2 and $d_{1}=d_{2}=0$ by aggregating the regularized multi-velocity and multi-temperature gas mixture PDEs [30]. In general case, it has recently been studied mathematically in [35] in general case.

Now, we sequentially define a number of functions involved in these PDEs. We assume that the mixture components are perfect polytropic gases and exploit the following expressions for the pressure, specific internal energy, the total energy and the specific enthalpy of the component α:

(4) $$\begin{array}{c} p_{\alpha }=\left(\gamma_{\alpha }-1\right)\rho_{\alpha }\varepsilon_{\alpha }=R_{\alpha }\rho_{\alpha }\theta ,\;\varepsilon_{\alpha }=c_{V\alpha }\theta ,\;E_{\alpha }=0.5\rho_{\alpha }{\left|u\right|}^{2}+\rho_{\alpha }\varepsilon_{\alpha },\;h_{\alpha }=\varepsilon_{\alpha }+\frac{p_{\alpha }}{\rho_{\alpha }}=c_{p\alpha }\theta , \end{array}$$

with physical constants $\gamma_{\alpha }=\frac{R_{\alpha }}{c_{V\alpha }}+1>1$, $R_{\alpha }>0$, $c_{V\alpha }>0$ and $c_{p\alpha }=c_{V\alpha }+R_{\alpha }=\gamma_{\alpha }c_{V\alpha }$, the last two of which are the specific heat capacities at constant volume and pressure, $\alpha =\bar{1,K}$. One can consider any two of four constants $\gamma_{\alpha }>1$, $R_{\alpha }>0$, $c_{V\alpha }>0$ and $c_{p\alpha }>0$ as the main independent ones; below, in computations in Section 5, such role is played by $\gamma_{\alpha }$ and $c_{V\alpha }$.

The total density and pressure, average specific internal energy and total energy of the mixture are expressed by the formulas

(5) $$\begin{array}{c} \rho =\langle \rho_{\alpha }\rangle ,\;\;p=\langle p_{\alpha }\rangle =R\rho \theta ,\;\;\varepsilon =\langle \frac{\rho_{\alpha }}{\rho }\varepsilon_{\alpha }\rangle =c_{V}\theta ,\;\;E=\langle E_{\alpha }\rangle =0.5\rho {\left|u\right|}^{2}+\rho \varepsilon , \end{array}$$

with the average gas mixture parameters

(6) $$\begin{array}{c} R:=\langle \frac{\rho_{\alpha }}{\rho }R_{\alpha }\rangle ,\;\;c_{V}:=\langle \frac{\rho_{\alpha }}{\rho }c_{V\alpha }\rangle . \end{array}$$

The second Formula (5) is the Dalton law for mixtures. The function $\frac{\rho_{\alpha }}{\rho }=:C_{\alpha }$ is the mass concentration of the mixture component α. Consequently, the important formula of the standard form for the total pressure holds as well

$$p=\left(\gamma -1\right)\rho \varepsilon ,\;\;\gamma :=\frac{R}{c_{V}}+1=\frac{c_{p}}{c_{V}}\;\;\mathrm{with}\;\;c_{p}:=\langle \frac{\rho_{\alpha }}{\rho }c_{p\alpha }\rangle .$$

In contrast to the single-component case, *R*, $c_{V}$ and γ are functions, not constants, except for the particular cases $R_{1}=\ldots =R_{K}$, $c_{V1}=\ldots =c_{VK}$ and $\gamma_{1}=\ldots \gamma_{K}$, respectively.

In the above PDEs, the following regularizing velocities for the component α and average ones were originally used

(7) $$\begin{array}{c} w_{\ell \alpha }=\ell \frac{\tau }{\rho_{\alpha }}\mathrm{div}\left(\rho_{\alpha }u\right)u+{\hat{w}}_{\alpha },\;\;{\hat{w}}_{\alpha }=\tau \left(\left(u\cdot \nabla \right)u+\frac{1}{\rho_{\alpha }}\nabla p_{\alpha }-f\right), \end{array}$$

(8) $$\begin{array}{c} w_{\ell }:=\langle \frac{\rho_{\alpha }}{\rho }w_{\ell \alpha }\rangle =\ell \frac{\tau }{\rho }\mathrm{div}\left(\rho u\right)u+\hat{w},\;\;\hat{w}:=\langle \frac{\rho_{\alpha }}{\rho }{\hat{w}}_{\alpha }\rangle =\tau \left(\left(u\cdot \nabla \right)u+\frac{1}{\rho }\nabla p-f\right), \end{array}$$

see [34], where τ=τ(ρ,u,θ)>0 is a regularization (relaxation) parameter which is usually a function, not constant, with $\rho :=(\rho_{1},\ldots ,\rho_{K})$. Here, ℓ=0 or 1, and the regularization is of the so-called quasi-gasdynamic (QGD) type for ℓ=1 or essentially simpler quasi-hydrodynamic (QHD) type for ℓ=0, so actually we consider two different, albeit related, systems in a unified manner similarly to [35]. The Formula (8) mean takes the average of $w_{\ell \alpha }$ and ${\hat{w}}_{\alpha }$, in other words, the full and partial averaging of $w_{\ell \alpha }$.

In this paper, we replace ${\hat{w}}_{\alpha }$ by $\hat{w}$ making $w_{\ell \alpha }$ combined and dependent on the total pressure *p* instead of the partial pressure $p_{\alpha }$:

(9) $$\begin{array}{c} w_{\ell \alpha }=\ell \frac{\tau }{\rho_{\alpha }}\mathrm{div}\left(\rho_{\alpha }u\right)u+\hat{w},\;\;\hat{w}=\tau \left(\left(u\cdot \nabla \right)u+\frac{1}{\rho }\nabla p-f\right) \end{array}$$

and analyze the effect of this replacement discussed above in Introduction. Notice that the replacement does not affect the validity of Formula (8) for $w_{\ell }$.

The viscosity tensor and heat flux are expressed, respectively, by the formulas

$$\Pi_{\ell }=\Pi^{NS}+\Pi_{\ell }^{\tau },\;\;q=q^{F}+q^{d}+\ell q^{\tau }$$

and contain the standard-type terms and the regularizing ones with the superscript τ. The classical Navier–Stokes viscosity tensor and the Fourier heat flux are given by the formulas

(10) $$\begin{array}{c} \Pi^{NS}=\mu \left(\nabla u+{\left(\nabla u\right)}^{T}\right)+\left(\lambda -\frac{2}{3}\mu \right)\left(\mathrm{div}u\right)I,\;\;-q^{F}=\varkappa \nabla \theta , \end{array}$$

where μ>0, λ⩾0 and ϰ>0 are the total coefficients of dynamic and bulk viscosities and heat conductivity (which can depend on the sought functions (ρ,u,θ)), $\nabla u={\left\{\partial_{i}u_{j}\right\}}_{i,j=1}^{n}$ and I is the unit tensor of order *n*.

Next, the regularizing viscosity tensor and heat flux are given by the formulas

(11) $$\begin{array}{c} \Pi_{\ell }^{\tau }=\rho u⊗\hat{w}+\ell \tau \left(u\cdot \nabla p+\langle \gamma_{\alpha }p_{\alpha }\rangle \mathrm{div}u-\langle \gamma_{\alpha }Q_{\alpha }\rangle +Q\right)I, \end{array}$$

(12) $$\begin{array}{c} -q^{\tau }=\tau \left\{\left(c_{V}\rho \nabla \theta -\theta \nabla \left(R\rho \right)\right)\cdot u-Q\right\}u. \end{array}$$

The density of body force f and intensities of heat sources $Q_{\alpha }⩾0$ (acting on the component α) are given functions, and $Q:=\langle Q_{\alpha }\rangle ⩾0$.

Finally, we consider the diffusion fluxes and additional respective heat flux of the form

(13) $$\begin{array}{c} \;-d_{\alpha }:={\langle d_{\alpha \beta }\left(\nabla \left(G_{\alpha }-G_{\beta }\right)+\left(e_{\alpha }-e_{\beta }\right)\nabla \theta \right)\rangle }_{\beta }={\langle d_{\alpha \beta }\left(\nabla G_{\alpha }+e_{\alpha }\nabla \theta -\left(\nabla G_{\beta }+e_{\beta }\nabla \theta \right)\right)\rangle }_{\beta }, \end{array}$$

(14) $$\begin{array}{c} q^{d}=\langle \left(G_{\alpha }+e_{\alpha }\theta \right)d_{\alpha }\rangle , \end{array}$$

(15) $$\begin{array}{c} G_{\alpha }:=\varepsilon_{\alpha }+\frac{p_{\alpha }}{\rho_{\alpha }}-s_{\alpha }\theta =h_{\alpha }-s_{\alpha }\theta =\left(c_{p\alpha }-s_{\alpha }\right)\theta ,\;\;s_{\alpha }=s_{\alpha 0}-R_{\alpha }\ln\frac{\rho_{\alpha }}{\rho_{\alpha 0}}+c_{V\alpha }\ln\frac{\theta }{\theta_{0}}, \end{array}$$

where ${\langle \cdot \rangle }_{\beta }$ means the summation over index $\beta =\bar{1,K}$. The functions $G_{\alpha }$ and $s_{\alpha }$ are the usual Gibbs potential and specific entropy of the component α, and $s_{\alpha 0}$, $\rho_{\alpha 0}>0$ and $\theta_{0}>0$ are constant reference values for $s_{\alpha }$, $\rho_{\alpha }$ and θ, $\alpha =\bar{1,K}$. The functions-coefficients $d_{\alpha \beta }$ and $e_{\alpha }$ can depend on the sought functions. Their specific form is not essential below, and we only assume the symmetry property $d_{\alpha \beta }=d_{\beta \alpha }$ for any α≠β.

Let ${\langle \cdot \rangle }_{\alpha ,\beta }:=\langle {\langle \cdot \rangle }_{\beta }\rangle$ mean the summation over $\alpha ,\beta =\bar{1,K}$. Using permutations of indices α and β and then this symmetry property, we obtain two identities

(16) $$\begin{array}{c} {\langle d_{\alpha \beta }\left(\varphi_{\alpha }-\varphi_{\beta }\right)\rangle }_{\alpha ,\beta }={\langle \left(d_{\alpha \beta }-d_{\beta \alpha }\right)\varphi_{\alpha }\rangle }_{\alpha ,\beta }=0,\\ {\langle d_{\alpha \beta }\left(\varphi_{\alpha }-\varphi_{\beta }\right)\psi_{\alpha }\rangle }_{\alpha ,\beta }\\ =0.5{\langle d_{\alpha \beta }\left(\varphi_{\alpha }-\varphi_{\beta }\right)\varphi_{\alpha }+d_{\beta \alpha }\left(\varphi_{\beta }-\varphi_{\alpha }\right)\psi_{\beta }\rangle }_{\alpha ,\beta }=0.5{\langle d_{\alpha \beta }\left(\varphi_{\alpha }-\varphi_{\beta }\right)\left(\psi_{\alpha }-\psi_{\beta }\right)\rangle }_{\alpha ,\beta } \end{array}$$

for any numbers $\varphi_{1},\ldots ,\varphi_{K}$ and $\psi_{1},\ldots ,\psi_{K}$. The first identity implies the important physical property $\langle d_{\alpha }\rangle =0$, and the second one will also be essential below.

We can avoid the explicit usage of $s_{\alpha }$ due to the formulas

(17) $$\begin{array}{c} \nabla G_{\alpha }+e_{\alpha }\nabla \theta =-\theta \nabla s_{\alpha }+\left(c_{p\alpha }-s_{\alpha }+e_{\alpha }\right)\nabla \theta =R_{\alpha }\frac{\theta }{\rho_{\alpha }}\nabla \rho_{\alpha }+{\bar{e}}_{\alpha }\nabla \theta \\ =\frac{1}{\rho_{\alpha }}\nabla p_{\alpha }+\left({\bar{e}}_{\alpha }-R_{\alpha }\right)\nabla \theta \;\;\mathrm{and}\;\;G_{\alpha }+e_{\alpha }\theta =\left(c_{V\alpha }+{\bar{e}}_{\alpha }\right)\theta ,\;\;\mathrm{with}\;\;{\bar{e}}_{\alpha }:=R_{\alpha }-s_{\alpha }+e_{\alpha }. \end{array}$$

In particular, for $e_{\alpha }=s_{\alpha }$, we obtain the simplest formulas

$$\nabla G_{\alpha }+s_{\alpha }\nabla \theta =R_{\alpha }\frac{\theta }{\rho_{\alpha }}\nabla \rho_{\alpha }+R_{\alpha }\nabla \theta =\frac{1}{\rho_{\alpha }}\nabla p_{\alpha },\;\;G_{\alpha }+s_{\alpha }\theta =h_{\alpha }.$$

The general multicomponent case (K⩾2) for ℓ=0,1 in the presence of $d_{\alpha }$ and $q^{d}$ has recently been studied mathematically in [35] but only in the particular case $d_{\alpha \beta }\equiv d_{0}$ and $Ke_{\alpha }-{\langle e_{\beta }\rangle }_{\beta }=b_{\alpha }$, that is the same for K=2 but much less general for K⩾3. The above quantities $d_{\alpha }$ and $q^{d}$ generalize those proposed in [1] in the case K=2; in this case, the formulas are transformed and discussed in more detail in [35]. Notice also that a much more general approach for introducing these quantities is known, for example, see [2].

Without the regularization, i.e., for τ=0, the above regularized system of PDEs is simplified and reduced to the compressible Navier–Stokes–Fourier-type system for the one-velocity and one-temperature multicomponent gas mixture dynamics for $\mu_{\alpha }>0$, $\lambda_{\alpha }>0$ and $\varkappa_{\alpha }>0$ or the Euler-type one for $\mu_{\alpha }=\lambda_{\alpha }=\varkappa_{\alpha }=0$, $\alpha =\bar{1,K}$, in particular, see [1,2,50] and references therein, and also Appendix A.

In [34], the above total coefficients μ, λ and ϰ are defined simply as

(18) $$\begin{array}{c} \mu =\langle \mu_{\alpha }\rangle ,\;\;\lambda =\langle \lambda_{\alpha }\rangle ,\;\;\varkappa =\langle \varkappa_{\alpha }\rangle , \end{array}$$

i.e., the sums of the corresponding coefficients of the components. These coefficients can be artificial depending on τ in order to ensure stability of symmetric in space discretizations for computations, or physical, or sums of them. In the first case, the typical formulas for τ and them are as follows

(19) $$\begin{array}{c} \tau =\frac{ah}{c_{s}+i_{\tau }\left|u\right|},\;\;\mu =\tau \langle a_{S\alpha }p_{\alpha }\rangle ,\;\;\lambda =\tau \langle a_{1S\alpha }p_{\alpha }\rangle ,\;\;\varkappa =\tau \langle a_{Pr\alpha }\gamma_{\alpha }c_{V\alpha }p_{\alpha }\rangle \end{array}$$

in accordance with Formula (18). Here, 0<a⩽1 is a parameter, $a_{S\alpha }>0$ and $a_{Pr\alpha }>0$ are the Schmidt and inverse Prandtl numbers for the component α; $a_{1S\alpha }⩾0$ is a counterpart of $a_{S\alpha }$ (in particular, $a_{1S\alpha }=0$), which can be also used as adjusting numerical parameters,

$$c_{s}=\sqrt{\gamma (\gamma -1)\varepsilon }=\sqrt{\gamma R\theta }$$

is the sound speed of the mixture, $i_{\tau }=0$ or 1, and *h* is a characteristic size of the spatial mesh. In the case of $a_{S\alpha }=a_{S}$, $a_{1S\alpha }=a_{1S}$ and $a_{Pr\alpha }=a_{Pr}$ independent of α, the formulas for μ, λ and ϰ are simplified:

$$\begin{array}{c} \mu =a_{S}\tau p,\;\;\lambda =a_{1S}\tau p,\;\;\varkappa =\tau a_{Pr}\langle \gamma_{\alpha }c_{V\alpha }p_{\alpha }\rangle . \end{array}$$

For the single-component gas dynamics, see such formulas, in particular, in [8,9,19].

Recall that $R_{\alpha }=\frac{R_{0}}{m_{\alpha }}$, where $R_{0}$ is the universal gas constant and $m_{\alpha }>0$ is the molecular mass of gas α. In some cases, $\gamma_{\alpha }$ and $m_{\alpha }$ are taken as the two main gas constants, and the average molecular mass of the mixture *m* is defined by $\frac{1}{m}=\langle \frac{\rho_{\alpha }}{\rho }\frac{1}{m_{\alpha }}\rangle .$ Then the other gas constants can be expressed in the form

$$c_{V\alpha }=\frac{R_{0}}{\left(\gamma_{\alpha }-1\right)m_{\alpha }},\;\;R=\frac{R_{0}}{m},\;\;c_{V}=R_{0}\langle \frac{\rho_{\alpha }}{\rho }\frac{1}{\left(\gamma_{\alpha }-1\right)m_{\alpha }}\rangle ,\;\;\gamma -1=\frac{R}{c_{V}}={\langle \frac{\rho_{\alpha }}{\rho }\frac{1}{\gamma_{\alpha }-1}\frac{m}{m_{\alpha }}\rangle }^{-1}.$$

We first give some inequalities for *R*, $c_{V}$, *m* and γ.

 **Proposition 1.** *1. The two-sided bounds hold*

$$\min_{\alpha =\bar{1,K}}R_{\alpha }⩽R⩽\max_{\alpha =\bar{1,K}}R_{\alpha },\;\;\min_{\alpha =\bar{1,K}}c_{V\alpha }⩽c_{V}⩽\max_{\alpha =\bar{1,K}}c_{V\alpha },\;\;\min_{\alpha =\bar{1,K}}m_{\alpha }⩽m⩽\max_{\alpha =\bar{1,K}}m_{\alpha }.$$

*Moreover, all the inequalities are strict, except for the particular cases $R_{1}=\ldots =R_{K}$, $c_{V1}=\ldots =c_{VK}$ and $m_{1}=\ldots =m_{K}$, respectively.*

*2. The formula and two-sided bounds also hold*

(20) $$\begin{array}{c} \min_{\alpha =\bar{1,K}}\gamma_{\alpha }⩽\gamma =\frac{R+c_{V}}{c_{V}}=\frac{\langle \rho_{\alpha }c_{V\alpha }\gamma_{\alpha }\rangle }{\langle \rho_{\alpha }c_{V\alpha }\rangle }⩽\max_{\alpha =\bar{1,K}}\gamma_{\alpha }. \end{array}$$

*Moreover, both the bounds are strict excluding the particular case $\gamma_{1}=\ldots =\gamma_{K}$; in that case, we have $\gamma =\gamma_{1}$ even if $c_{V\alpha }$ are not identical for all $\alpha =\bar{1,K}$.*

*3. The following relations hold*

(21) $$\begin{array}{c} \;\langle \gamma_{\alpha }p_{\alpha }\rangle =\tilde{\gamma }p⩾\gamma p\;\;\mathrm{with}\;\;\tilde{\gamma }:=\frac{\langle \rho_{\alpha }R_{\alpha }\gamma_{\alpha }\rangle }{\langle \rho_{\alpha }R_{\alpha }\rangle }=\langle \frac{\rho_{\alpha }}{\rho }\frac{m}{m_{\alpha }}\gamma_{\alpha }\rangle \Leftrightarrow \langle \frac{\rho_{\alpha }}{\rho }c_{s\alpha }^{2}\rangle ⩾c_{s}^{2}=\gamma \left(\gamma -1\right)\varepsilon . \end{array}$$

*with $c_{s\alpha }^{2}=\gamma_{\alpha }\left(\gamma_{\alpha }-1\right)\varepsilon_{\alpha }$. Here, $\langle \gamma_{\alpha }p_{\alpha }\rangle =\gamma p$, or $\tilde{\gamma }=\gamma$, in the case $\gamma_{1}=\ldots =\gamma_{K}$ only.*

 **Proof.**  Items 1 and 2 are elementary. Item 3 is valid since

(22) $$\begin{array}{c} \langle \gamma_{\alpha }p_{\alpha }\rangle -\gamma p=\langle \left(\gamma_{\alpha }-1\right)p_{\alpha }\rangle -\left(\gamma -1\right)p=\left(\langle \frac{R_{\alpha }^{2}}{c_{V\alpha }}\rho_{\alpha }\rangle -\frac{{\langle \rho_{\alpha }R_{\alpha }\rangle }^{2}}{\langle \rho_{\alpha }c_{V\alpha }\rangle }\right)\theta ⩾0 \end{array}$$

owing to the Cauchy inequality

$${\langle \rho_{\alpha }R_{\alpha }\rangle }^{2}={\langle R_{\alpha }\sqrt{\frac{\rho_{\alpha }}{c_{V\alpha }}}\sqrt{\rho_{\alpha }c_{V\alpha }}\rangle }^{2}⩽\langle \frac{R_{\alpha }^{2}}{c_{V\alpha }}\rho_{\alpha }\rangle \langle c_{V\alpha }\rho_{\alpha }\rangle .$$

The inequality becomes an equality only for $\frac{R_{\alpha }^{2}}{c_{V\alpha }^{2}}$ independent of α, i.e., $\gamma_{1}=\ldots =\gamma_{K}$. □

 **Remark 1.** *Starting from Formula (22), we can accomplish the following transformations*

$$\begin{array}{c} \left(\langle \gamma_{\alpha }p_{\alpha }\rangle -\gamma p\right)\frac{\langle \rho_{\alpha }c_{V\alpha }\rangle }{\theta }=\langle {\left(\gamma_{\alpha }-1\right)}^{2}c_{V\alpha }\rho_{\alpha }\rangle \langle \rho_{\alpha }c_{V\alpha }\rangle -{\langle \left(\gamma_{\alpha }-1\right)\rho_{\alpha }c_{V\alpha }\rangle }^{2}\\ ={\langle \left({\left(\gamma_{\alpha }-1\right)}^{2}-\left(\gamma_{\alpha }-1\right)\left(\gamma_{\beta }-1\right)\right)\rho_{\alpha }\rho_{\beta }c_{V\alpha }c_{V\beta }\rangle }_{\alpha ,\beta }={\langle \left(\gamma_{\alpha }-1\right)\left(\gamma_{\alpha }-\gamma_{\beta }\right)\rho_{\alpha }\rho_{\beta }c_{V\alpha }c_{V\beta }\rangle }_{\alpha ,\beta }. \end{array}$$

*Permuting indexes α and β, similarly to identity (16), we derive the representation*

$$\begin{array}{c} \langle \gamma_{\alpha }p_{\alpha }\rangle -\gamma p=\frac{\theta }{2\langle c_{V\alpha }\rho_{\alpha }\rangle }{\langle {\left(\gamma_{\alpha }-\gamma_{\beta }\right)}^{2}\rho_{\alpha }\rho_{\beta }c_{V\alpha }c_{V\beta }\rangle }_{\alpha ,\beta }⩾0 \end{array}$$

*since $\left(\gamma_{\alpha }-1\right)\left(\gamma_{\alpha }-\gamma_{\beta }\right)+\left(\gamma_{\beta }-1\right)\left(\gamma_{\beta }-\gamma_{\alpha }\right)={\left(\gamma_{\alpha }-\gamma_{\beta }\right)}^{2}$. This formula also implies Item 3.*

Formulas for γ and $\tilde{\gamma }$ in relations (20) and (21) show that both of them are averages of $\gamma_{1},\ldots ,\gamma_{K}$, and clearly $c_{V\alpha }$ and $R_{\alpha }$ can be scaled in these formulas. For $\tilde{\gamma }$, the same bounds as in Item 2 for γ are valid. Note that, in our expression for $\Pi^{\tau }$, see (11), we use the term $\langle \gamma_{\alpha }p_{\alpha }\rangle$ as in [34,35], in contrast to γp in [39]. Moreover, both sides of the second inequality (21) can be considered as different definitions for the squared sound speed of the mixture, and we prefer to use the right-hand side in this role in this paper (Appendix C helps to make the choice).

We first consider the sought functions of the particular form.

 **Proposition 2.** *Let $0<C_{\alpha }<1$, $\alpha =\bar{1,K}$, be arbitrary constants such that $\langle C_{\alpha }\rangle =1$. Consider the sought functions of the particular form $\rho_{\alpha }=C_{\alpha }\rho$ with ρ>0 ($\alpha =\bar{1,K}$), u and θ>0 and the case of $d_{\alpha }=0$ and ψ=ψ(ρ,u,θ) for the functions ψ=τ,μ,λ,ϰ. For them, the above regularized system of PDEs for the gas mixture dynamics is reduced to the following regularized system of PDEs for a single-component gas dynamics*

(23) $$\begin{array}{c} \partial_{t}\rho +\mathrm{div}\left(\rho (u-w_{\ell })\right)=0, \end{array}$$

(24) $$\begin{array}{c} \partial_{t}\left(\rho u\right)+\mathrm{div}\left(\rho (u-w_{\ell })⊗u\right)+\nabla p=\mathrm{div}\Pi_{\ell }+\left(\rho -\ell \tau \mathrm{div}(\rho u)\right)f, \end{array}$$

(25) $$\begin{array}{c} \partial_{t}E+\mathrm{div}\left(\left(E+p\right)\left(u-w_{\ell }\right)\right)=\mathrm{div}\left(-q+\Pi_{\ell }u\right)+\rho \left(u-w_{\ell }\right)\cdot f+Q \end{array}$$

*for the sought functions ρ, u and θ and (x,t)∈Ω×[0,T]. Here,*

(26) $$\begin{array}{c} p=\left(\gamma -1\right)\rho \varepsilon =R\rho \theta ,\;\;\varepsilon =c_{V}\theta ,\;\;E=0.5\rho {\left|u\right|}^{2}+\rho \varepsilon ,\;R=\langle C_{\alpha }R_{\alpha }\rangle ,\;\;c_{V}=\langle C_{\alpha }c_{V\alpha }\rangle \end{array}$$

*with constant $\gamma =\frac{R}{c_{V}}+1$, R and $c_{V}$, together with*

(27) $$\begin{array}{c} w_{\ell }=\ell \frac{\tau }{\rho }\mathrm{div}\left(\rho u\right)u+\hat{w},\;\;\hat{w}=\tau \left(\left(u\cdot \nabla \right)u+\frac{1}{\rho }\nabla p-f\right),\\ \Pi_{\ell }=\Pi^{NS}+\Pi_{\ell }^{\tau },\;\;\Pi_{\ell }^{\tau }=\rho u⊗\hat{w}+\ell \tau \left(u\cdot \nabla p+\tilde{\gamma }p\mathrm{div}u-\left(\gamma_{1}-1\right)Q\right)I, \end{array}$$

(28) $$\begin{array}{c} \;q=q^{F}+\ell q^{\tau },\;-q^{\tau }=\tau \left\{\left(c_{V}\rho \nabla \theta -R\theta \nabla \rho \right)\cdot u-Q\right\}u=\tau \left\{\left(\rho \nabla \varepsilon -\frac{p}{\rho }\nabla \rho \right)\cdot u-Q\right\}u, \end{array}$$

*where $\tilde{\gamma }=\langle C_{\alpha }R_{\alpha }\gamma_{\alpha }\rangle /\langle C_{\alpha }R_{\alpha }\rangle$ and the above formulas (10) for $\Pi^{NS}$ and $q^{F}$ are in use.*

 **Proof.**  For $\rho_{\alpha }=C_{\alpha }\rho$, under the assumptions made about $C_{\alpha }$, we clearly obtain $\rho =\langle \rho_{\alpha }\rangle$ and

$$w_{\ell \alpha }=\ell \frac{\tau }{\rho }\mathrm{div}\left(\rho u\right)u+\hat{w}=w_{\ell },\;\;\langle \rho_{\alpha }h_{\alpha }\rangle =\rho \langle C_{\alpha }c_{p\alpha }\rangle \theta =\rho \varepsilon +p.$$

Thus, all the balance PDEs for the mass of components (1), after division by $C_{\alpha }$, are reduced to Equation (23). Moreover, expressions (11) for $\Pi_{\ell }^{\tau }$ and (12) for $-q^{\tau }$ are reduced to those given in Formulas (27) and (28), using the expression for $\tilde{\gamma }$ in relations (21). Therefore, now the original balance PDEs for the total momentum and total energy (2) and (3) take forms (24) and (25). □

This proposition establishes a particular connection between solutions to the regularized systems of PDEs for the gas mixture dynamics and single-component gas dynamics for any $\gamma_{\alpha }>1$ and $c_{V\alpha }>0$, $\alpha =\bar{1,K}$, and can be useful to check properties of the former system. In fact, it enlarges the corresponding 1D Proposition 1 in [41]. However, recall that $\tilde{\gamma }=\gamma$ in Formula (27) only in the particular case $\gamma_{1}=\ldots =\gamma_{K}$, see Proposition 1, Item 3 or Remark 1.

Applying the operation 〈·〉 to the mass balance equation for the mixture components (1), using the formula $\langle \rho_{\alpha }\left(u-w_{\ell \alpha }\right)\rangle =\rho \left(u-w_{\ell }\right)$ valid according to the first expression (8), and the property $\langle d_{\alpha }\rangle =0$, we obtain the important total mass balance equation

(29) $$\partial_{t}\rho +\mathrm{div}\left(\rho \left(u-w_{\ell }\right)\right)=0.$$

Here, $\rho_{1},\ldots ,\rho_{K}$ and θ appear only implicitly since $\rho =\langle \rho_{\alpha }\rangle$ and *p* in $w_{\ell }$ depend on them.

The balance PDEs for the total momentum and total energy of the mixture (2) and (3) entail sequentially the balance PDEs for the kinetic and internal energies of the mixture

(30) $$\begin{array}{c} \;0.5\partial_{t}{\left(\rho |u\right|}^{2})+0.5\mathrm{div}\left({\rho |u|}^{2}\left(u-w_{\ell }\right)\right)+\left(\nabla p\right)\cdot u=\left(\mathrm{div}\Pi \right)\cdot u+\left(\rho -\ell \tau \mathrm{div}(\rho u)\right)f\cdot u, \end{array}$$

(31) $$\begin{array}{c} \;\partial_{t}\left(\rho \varepsilon \right)+\mathrm{div}\langle \rho_{\alpha }\varepsilon_{\alpha }\left(u-w_{\ell \alpha }\right)\rangle +p\mathrm{div}u=\mathrm{div}\left(-q+\langle p_{\alpha }w_{\ell \alpha }\rangle \right)+\Pi :\nabla u-\rho \hat{w}\cdot f+Q, \end{array}$$

where the symbol: denotes the scalar product of tensors. The derivation exploits the total mass balance equation (29) and is valid for any $w_{\ell }$, for the former equation, and exploits only the relation $w_{\ell }=\ell \tau \mathrm{div}\left(\rho u\right)u+\hat{w}$ (for f¬≡0), but not explicitly Formulas (7) and (8), for the latter equation, see ([35], Section 4.1).

The first main result of the paper concerns the total entropy balance equation. Recall that the specific entropy of the mixture is given by the formula $s=\langle \frac{\rho_{\alpha }}{\rho }s_{\alpha }\rangle$. The result corresponds to ([35], Theorem 3.1) but concerns another definition of the regularizing velocity (9) and deals with much more general form of $d_{\alpha }$, for K⩾3; this form is applicable in [35,37] as well.

 **Theorem 1.** *Let $d_{\alpha \beta }=d_{\beta \alpha }⩾0$ for any α≠β. The following regularized entropy balance equation for the multicomponent mixture in the presence of diffusion fluxes holds*

$$\begin{array}{c} \partial_{t}\left(\rho s\right)+\mathrm{div}\left\{\langle \rho_{\alpha }s_{\alpha }\left(u-w_{\ell \alpha }\right)\rangle +\langle e_{\alpha }d_{\alpha }\rangle +\frac{1}{\theta }\left(q^{F}+\ell q^{\tau }\right)\right\}=P^{NS}+P^{\tau } \end{array}$$

*with the entropy production $P^{NS}+P^{\tau }$, where*

$$\begin{array}{c} P^{NS}=\frac{1}{\theta }\{\frac{\mu }{2}|\nabla u+\nabla u^{T}{|}_{F}^{2}+\left(\lambda -\frac{2}{3}\mu \right){\left(\mathrm{div}u\right)}^{2}+\frac{1}{\theta }\varkappa {\left|\nabla \theta \right|}^{2}\\ +\frac{1}{2}{\langle d_{\alpha \beta }|\nabla \left(G_{\alpha }-G_{\beta }\right)+\left(e_{\alpha }-e_{\beta }\right)\nabla \theta {|}^{2}\rangle }_{\alpha ,\beta }\}⩾0,\\ P^{\tau }=\frac{\rho }{\tau \theta }{|\hat{w}|}^{2}+\ell \langle \tau \frac{R_{\alpha }}{\rho_{\alpha }}{\left(\mathrm{div}\left(\rho_{\alpha }u\right)\right)}^{2}+\tau c_{V\alpha }\rho_{\alpha }{\left(u\cdot \nabla \ln\theta +\left(\gamma_{\alpha }-1\right)\mathrm{div}u-\frac{\left(\gamma_{\alpha }-1\right)Q_{\alpha }}{2p_{\alpha }}\right)}^{2}\rangle \\ +\langle \frac{Q_{\alpha }}{\theta }\left(1-\ell \frac{\tau \left(\gamma_{\alpha }-1\right)Q_{\alpha }}{4p_{\alpha }}\right)\rangle , \end{array}$$

*and ${\left|\cdot \right|}_{F}$ is the Frobenius norm. Moreover, $P^{\tau }$ is non-negative for ℓ=0, as well as for ℓ=1 under the condition*

$$\tau \langle \frac{\left(\gamma_{\alpha }-1\right)Q_{\alpha }^{2}}{4p_{\alpha }}\rangle ⩽Q.$$

*This condition is certainly true provided that $\tau \left(\gamma_{\alpha }-1\right)Q_{\alpha }⩽4p_{\alpha }$, $\alpha =\bar{1,K}$.*

 **Proof.**  According to ([35], proof of Theorem 3.1), the following preliminary equation involving the entropies of the mixture and the components holds

(32) $$\begin{array}{c} \;\partial_{t}\left(\rho s\right)+\mathrm{div}\left\{\langle \rho_{\alpha }s_{\alpha }\left(u-w_{\ell \alpha }\right)+e_{\alpha }d_{\alpha }\rangle +\frac{1}{\theta }\left(q^{F}+\ell q^{\tau }\right)\right\}=-\frac{1}{\theta }\nabla \theta \cdot \langle e_{\alpha }d_{\alpha }\rangle -\frac{1}{\theta }\langle \nabla G_{\alpha }\cdot d_{\alpha }\rangle \\ +\frac{1}{\theta^{2}}\varkappa {\left|\nabla \theta \right|}^{2}+\frac{1}{\theta }\left\{\frac{\mu }{2}{|\nabla u+\nabla u^{T}|}^{2}+\left(\lambda -\frac{2}{3}\mu \right){\left(\mathrm{div}u\right)}^{2}\right\}+\frac{1}{\theta }B^{\tau }, \end{array}$$

where

$$B^{\tau }:=\langle \nabla p_{\alpha }\cdot w_{\ell \alpha }\rangle -\ell q^{\tau }\cdot \frac{1}{\theta }\nabla \theta +\Pi^{\tau }:\nabla u-\rho \hat{w}\cdot f+Q.$$

This equation is derived from the balance PDEs (1) and (31) and does not exploit specific expressions for $w_{\ell \alpha }$ and $\hat{w}$ in them. In the first term on the right, we have taken into account the following obvious formula

$$\begin{array}{c} \langle \frac{G_{\alpha }}{\theta }d_{\alpha }\rangle -\frac{q^{d}}{\theta }=-\langle e_{\alpha }d_{\alpha }\rangle , \end{array}$$

see definition (14) of $q^{d}$, that only slightly differs from the similar formula in [35].

Using identity (16), we can write the first and second terms on the right in Equation (32) in the form

$$\begin{array}{c} -\frac{1}{\theta }\nabla \theta \cdot \langle e_{\alpha }d_{\alpha }\rangle -\frac{1}{\theta }\langle \nabla G_{\alpha }\cdot d_{\alpha }\rangle =-\frac{1}{\theta }\langle \left(\nabla G_{\alpha }+e_{\alpha }\nabla \theta \right)\cdot d_{\alpha }\rangle \\ =\frac{1}{2\theta }{\langle d_{\alpha \beta }{\left(\nabla G_{\alpha }+e_{\alpha }\nabla \theta -\left(\nabla G_{\beta }+e_{\beta }\nabla \theta \right)\right)}^{2}\rangle }_{\alpha ,\beta }. \end{array}$$

Next, in the case of expressions (9), we can extract and collect the terms with $\hat{w}$ from the first, third and fourth terms of $B^{\tau }$ and thus write

$$\begin{array}{c} B^{\tau }=\langle \nabla p_{\alpha }\cdot \hat{w}\rangle +\left(u\cdot \nabla \right)u\cdot \rho \hat{w}-\rho \hat{w}\cdot f+{\tilde{B}}^{\tau }\\ =\left(\rho (u\cdot \nabla )u+\nabla p-\rho f\right)\cdot \hat{w}+{\tilde{B}}^{\tau }=\frac{\rho }{\tau }{\left|\hat{w}\right|}^{2}+{\tilde{B}}^{\tau }. \end{array}$$

Concerning the remainder ${\tilde{B}}^{\tau }$, the following formula holds $\frac{1}{\theta }{\tilde{B}}^{\tau }=P^{\tau }-\frac{\rho }{\tau \theta }{\left|\hat{w}\right|}^{2},$ see ([35], proof of Theorem 3.1) and the references therein, that completes the proof. □

Clearly, $P^{NS}+\frac{Q}{\theta }$ and $P^{\tau }-\frac{Q}{\theta }$ are the Navier–Stokes–Fourier and regularizing contributions to the entropy production. Theorem 1 remains valid for τ⩾0 (in particular, τ=0, i.e., without a regularization), when one should pass to a different form for the first relaxation term:

$$\frac{\rho }{\tau \theta }|\hat{w}{|}^{2}=\frac{\tau }{\rho \theta }{\left|\rho \left(u\cdot \nabla \right)u+\nabla p-\rho f\right|}^{2}.$$

## 3. Linearized Regularized System of PDEs for Gas Mixture Dynamics, Its Properties and Corollaries

### 3.1. An Auxiliary Reduction of the Balance Equations

Let f=0 and $Q_{1}=\ldots =Q_{K}=0$. We introduce the vector of the sought functions z:=(ρ,u,θ) and first present an important auxiliary reduction of the balance PDEs for $\rho_{\alpha }$, u and θ up to the terms $O(|\nabla z{|}^{2})$.

 **Lemma 1.** *The following reduced PDEs hold: for the densities of the components*

(33) $$\begin{array}{c} \partial_{t}\rho_{\alpha }+\nabla \rho_{\alpha }\cdot u+\rho_{\alpha }\mathrm{div}u\\ =\tau \left[\frac{\rho_{\alpha }\theta }{\rho }{\langle R_{\beta }\Delta \rho_{\beta }\rangle }_{\beta }+\ell \left[u\cdot \left(u\cdot \nabla \right)\nabla \right]\rho_{\alpha }+\left(\ell +1\right)\rho_{\alpha }\left(u\cdot \nabla \right)\mathrm{div}u+R\rho_{\alpha }\Delta \theta \right]\\ +\theta {\langle d_{\alpha \beta }\left(\frac{R_{\alpha }}{\rho_{\alpha }}\Delta \rho_{\alpha }-\frac{R_{\beta }}{\rho_{\beta }}\Delta \rho_{\beta }\right)\rangle }_{\beta }+{\langle d_{\alpha \beta }\left({\bar{e}}_{\alpha }-{\bar{e}}_{\beta }\right)\rangle }_{\beta }\Delta \theta +{O(|\nabla z|}^{2}),\;\;\alpha =\bar{1,K}, \end{array}$$

*for the velocity*

(34) $$\begin{array}{c} \partial_{t}u+\frac{\theta }{\rho }\langle R_{\alpha }\nabla \rho_{\alpha }\rangle +\left(u\cdot \nabla \right)u+R\nabla \theta \\ =\left(\ell +1\right)\tau \frac{\theta }{\rho }\left(u\cdot \nabla \right)\langle R_{\alpha }\nabla \rho_{\alpha }\rangle +\frac{\mu }{\rho }\Delta u+\frac{\chi }{\rho }\nabla \mathrm{div}u+\ell \tau \frac{\langle \gamma_{\alpha }p_{\alpha }\rangle }{\rho }\nabla \mathrm{div}u\\ +\tau \left[u\cdot \left(u\cdot \nabla \right)\nabla \right]u+\left(\ell +1\right)\tau R\left(u\cdot \nabla \right)\nabla \theta +O(|\nabla z{|}^{2}), \end{array}$$

*with $\chi :=\frac{1}{3}\mu +\lambda$, and for the temperature*

(35) $$\begin{array}{c} \partial_{t}\theta +\frac{R}{c_{V}}\theta \mathrm{div}u+u\cdot \nabla \theta \\ =\tau \frac{R\theta^{2}}{c_{V}\rho }\langle R_{\alpha }\Delta \rho_{\alpha }\rangle +\left(\ell +1\right)\tau \frac{R\theta }{c_{V}}\left(u\cdot \nabla \right)\mathrm{div}u+\ell \tau \left[u\cdot \left(u\cdot \nabla \right)\nabla \right]\theta +\left(\frac{\varkappa }{c_{V}\rho }+\tau \frac{R^{2}\theta }{c_{V}\rho }\right)\Delta \theta \\ +\frac{\theta }{c_{V}\rho }{\langle d_{\alpha \beta }\left(\frac{R_{\alpha }}{\rho_{\alpha }}\Delta \rho_{\alpha }-\frac{R_{\beta }}{\rho_{\beta }}\Delta \rho_{\beta }\right){\bar{e}}_{\alpha }\rangle }_{\alpha ,\beta }+\frac{1}{c_{V}\rho }{\langle d_{\alpha \beta }\left({\bar{e}}_{\alpha }-{\bar{e}}_{\beta }\right){\bar{e}}_{\alpha }\rangle }_{\alpha ,\beta }\Delta \theta +{O(|\nabla z|}^{2}). \end{array}$$

*Hereafter, Δ=div∇ is the Laplace operator and ${\bar{e}}_{\alpha }=R_{\alpha }-s_{\alpha }+e_{\alpha }$, see the last formula (17).*

 **Proof.**  According to ([35], Section 3.2), the balance equation for the velocity holds

(36) $$\begin{array}{c} \partial_{t}u+\left(\left(u-w_{\ell }\right)\cdot \nabla \right)u+\frac{1}{\rho }\nabla p=\frac{1}{\rho }\{\mathrm{div}\Pi^{NS}+\left(u\cdot \nabla \right)\left(\rho \hat{w}\right)+\left(\mathrm{div}u\right)\left(\rho \hat{w}+\ell \nabla \langle \tau \gamma_{\alpha }p_{\alpha }\rangle \right)\\ +\ell \tau \langle \gamma_{\alpha }p_{\alpha }\rangle \nabla \mathrm{div}u+\ell \nabla \left(\tau u\cdot \nabla p-\tau (\langle \gamma_{\alpha }Q_{\alpha }\rangle -Q)\right)\}+\left(1-\ell \frac{\tau }{\rho }\mathrm{div}\left(\rho u\right)\right)f, \end{array}$$

and the balance equation for the temperature holds

(37) $$\begin{array}{c} \partial_{t}\theta +\left(u-\frac{\langle c_{V\alpha }\rho_{\alpha }w_{\ell \alpha }\rangle }{c_{V}\rho }\right)\cdot \nabla \theta +\frac{R}{c_{V}}\theta \mathrm{div}u\\ =\frac{1}{c_{V}\rho }\left\{\langle c_{V\alpha }\mathrm{div}d_{\alpha }\rangle \theta +\mathrm{div}\left(-q+\langle p_{\alpha }w_{\ell \alpha }\rangle \right)+\Pi :\nabla u-\rho \hat{w}\cdot f+Q\right\} \end{array}$$

as well. Their derivation does not exploit a specific form of $w_{\ell \alpha }$. For $d_{1}=\ldots =d_{K}=0$, the presented reductions have recently been proved in [37] for ℓ=0, and the similar reductions have also been accomplished in [35], where the terms with multiplier *ℓ* are the same as here, whereas the other terms are partially different.

So, it suffices to reduce the terms with $d_{\alpha }$. In the balance PDEs for the mass of components (1), we obtain

(38) $$\begin{array}{c} -\mathrm{div}d_{\alpha }={\langle d_{\alpha \beta }\left(\Delta \left(G_{\alpha }-G_{\beta }\right)+\left(e_{\alpha }-e_{\beta }\right)\Delta \theta \right)\rangle }_{\beta }+{O(|\nabla z|}^{2})\\ =\theta {\langle d_{\alpha \beta }\left(\frac{R_{\alpha }}{\rho_{\alpha }}\Delta \rho_{\alpha }-\frac{R_{\beta }}{\rho_{\beta }}\Delta \rho_{\beta }\right)\rangle }_{\beta }+{\langle d_{\alpha \beta }\left({\bar{e}}_{\alpha }-{\bar{e}}_{\beta }\right)\rangle }_{\beta }\Delta \theta +{O(|\nabla z|}^{2}),\;\;\alpha =\bar{1,K}, \end{array}$$

since the following chain of transformations

$$\begin{array}{c} \Delta G_{\alpha }=-\theta \Delta s_{\alpha }+\left(c_{p\alpha }-s_{\alpha }\right)\Delta \theta +{O(|\nabla z|}^{2})=\theta \left(\frac{R_{\alpha }}{\rho_{\alpha }}\Delta \rho_{\alpha }-\frac{c_{V\alpha }}{\theta }\Delta \theta \right)+\left(c_{p\alpha }-s_{\alpha }\right)\Delta \theta +{O(|\nabla z|}^{2})\\ =\theta \frac{R_{\alpha }}{\rho_{\alpha }}\Delta \rho_{\alpha }+\left(c_{p\alpha }-c_{V\alpha }-s_{\alpha }\right)\Delta \theta +{O(|\nabla z|}^{2}) \end{array}$$

is valid and $c_{p\alpha }-c_{V\alpha }=R_{\alpha }$. In the balance equation for the temperature (37), we can write

$$\begin{array}{c} \langle c_{V\alpha }\mathrm{div}d_{\alpha }\rangle \theta +\mathrm{div}\left(-q^{d}\right)=\langle c_{V\alpha }\mathrm{div}d_{\alpha }\rangle \theta -\left(G_{\alpha }+e_{\alpha }\theta \right)\mathrm{div}d_{\alpha }+{O(|\nabla z|}^{2})\\ =-\langle \left(\mathrm{div}d_{\alpha }\right)\left(c_{p\alpha }-c_{V\alpha }-s_{\alpha }+e_{\alpha }\right)\theta \rangle +{O(|\nabla z|}^{2})\\ =\theta^{2}{\langle d_{\alpha \beta }\left(\frac{R_{\alpha }}{\rho_{\alpha }}\Delta \rho_{\alpha }-\frac{R_{\beta }}{\rho_{\beta }}\Delta \rho_{\beta }\right){\bar{e}}_{\alpha }\rangle }_{\alpha ,\beta }+\theta {\langle d_{\alpha \beta }\left({\bar{e}}_{\alpha }-{\bar{e}}_{\beta }\right){\bar{e}}_{\alpha }\rangle }_{\alpha ,\beta }\Delta \theta +{O(|\nabla z|}^{2}) \end{array}$$

using the previous decomposition (38). This completes the proof. □

The systems of PDEs (1)–(3) and (1), (36), (37) (taking into account formula (11)) are equivalent for classical (smooth) solutions. Below the reduced system of PDEs (33)–(35) helps to linearize the original system of PDEs and perform its parabolicity analysis. Clearly, the left-hand sides of these PDEs are independent of τ and *ℓ*.

### 3.2. Linearized Regularized System of PDEs, Its Properties and Corollaries

In the case f=0 and $Q_{1}=\ldots =Q_{K}=0$, the system of PDEs (1)–(3) has constant solutions

$$\left(\rho ,u,\theta \right)\left(x,t\right)\equiv z_{0}=\left(\rho_{0},u_{0},\theta_{0}\right),\;\mathrm{with}\;\mathrm{any}\;\rho_{0}:=\left(\rho_{10},\ldots ,\rho_{K0}\right),\;\rho_{10}>0,\ldots ,\rho_{K0}>0,\;\theta_{0}>0$$

and any $u_{0}$. We perform the linearization of the solution z to this system on $z_{0}$ and write

$$\begin{array}{c} \rho_{\alpha }=\rho_{\alpha 0}+\rho_{\alpha *}{\tilde{\rho }}_{\alpha }\;\;\left(\alpha =\bar{1,K}\right),\;\;u=u_{0}+u_{*}\tilde{u},\;\;\theta =\theta_{0}+\theta_{*}\tilde{\theta }, \end{array}$$

where $\tilde{z}:=\left(\tilde{\rho },\tilde{u},\tilde{\theta }\right)$ with $\tilde{\rho }:=\left({\tilde{\rho }}_{1},\ldots ,{\tilde{\rho }}_{K}\right)$ is the vector of dimensionless perturbations and $\rho_{\alpha *}>0$, $u_{*}>0$ and $\theta_{*}>0$ are scaling parameters selected below. We substitute the solution in this form into the reduced system of PDEs (33)–(35) and discard the terms of the second order of smallness with respect to $\tilde{z}$ and its first and second order derivatives using the formula $\nabla z=(\rho_{1*}\nabla {\tilde{\rho }}_{1},\ldots ,\rho_{K*}\nabla {\tilde{\rho }}_{K},u_{*}\nabla \tilde{u},\theta_{*}\nabla \tilde{\theta }).$ Then, we divide the resulting PDEs by $\rho_{\alpha *}$, $u_{*}$ and $\theta_{*}$, respectively, and derive the linearized system of PDEs with constant coefficients for $\tilde{z}$:

$$\begin{array}{c} \partial_{t}{\tilde{\rho }}_{\alpha }+u_{*}\left({\hat{u}}_{0}\cdot \nabla {\tilde{\rho }}_{\alpha }+{\hat{\rho }}_{\alpha 0}\mathrm{div}\tilde{u}\right)\\ =\tau_{0}u_{*}^{2}\left\{\frac{{\hat{\rho }}_{\alpha 0}\theta_{0}}{\rho_{0}u_{*}^{2}}{\langle R_{\beta }\rho_{\beta *}\Delta {\tilde{\rho }}_{\beta }\rangle }_{\beta }+\ell {\left({\hat{u}}_{0}\cdot \nabla \right)}^{2}{\tilde{\rho }}_{\alpha }+\left(\ell +1\right){\hat{\rho }}_{\alpha 0}\left({\hat{u}}_{0}\cdot \nabla \right)\mathrm{div}\tilde{u}+\frac{R_{0}{\hat{\rho }}_{\alpha 0}\theta_{*}}{u_{*}^{2}}\Delta \tilde{\theta }\;\right\}\\ +\theta_{0}{\langle d_{\alpha \beta 0}\left(\frac{R_{\alpha }}{\rho_{\alpha 0}}\Delta {\tilde{\rho }}_{\alpha }-\frac{R_{\beta }\rho_{\beta *}}{\rho_{\beta 0}\rho_{\alpha *}}\Delta {\tilde{\rho }}_{\beta }\right)\rangle }_{\beta }+\frac{\theta_{*}}{\rho_{\alpha *}}{\langle d_{\alpha \beta 0}\left({\bar{e}}_{\alpha 0}-{\bar{e}}_{\beta 0}\right)\rangle }_{\beta }\Delta \tilde{\theta },\;\;\alpha =\bar{1,K},\\ \partial_{t}\tilde{u}+u_{*}\left(\frac{\theta_{0}}{\rho_{0}u_{*}^{2}}{\langle R_{\alpha }\rho_{\alpha *}\nabla {\tilde{\rho }}_{\alpha }\rangle }_{\beta }+\left({\hat{u}}_{0}\cdot \nabla \right)\tilde{u}+\frac{R_{0}\theta_{*}}{u_{*}^{2}}\nabla \tilde{\theta }\;\right)=u_{*}^{2}\{\left(\ell +1\right)\tau_{0}\frac{\theta_{0}}{\rho_{0}u_{*}^{2}}\left({\hat{u}}_{0}\cdot \nabla \right)\langle R_{\alpha }\rho_{\alpha *}\nabla {\tilde{\rho }}_{\alpha }\rangle \\ +\frac{\mu_{0}}{\rho_{0}u_{*}^{2}}\Delta \tilde{u}+\left(\frac{\chi_{0}}{\rho_{0}u_{*}^{2}}+\ell \tau_{0}\frac{{\left(R\gamma \right)}_{0}\theta_{0}}{u_{*}^{2}}\right)\nabla \mathrm{div}\tilde{u}+\tau_{0}{\left({\hat{u}}_{0}\cdot \nabla \right)}^{2}\tilde{u}+\left(\ell +1\right)\tau_{0}\frac{R_{0}\theta_{*}}{u_{*}^{2}}\left({\hat{u}}_{0}\cdot \nabla \right)\nabla \tilde{\theta }\;\},\\ \partial_{t}\tilde{\theta }+u_{*}\left(\frac{R_{0}{\hat{\theta }}_{0}}{c_{V0}}\mathrm{div}\tilde{u}+{\hat{u}}_{0}\cdot \nabla \tilde{\theta }\;\right)=u_{*}^{2}\{\tau_{0}\frac{R_{0}{\hat{\theta }}_{0}^{\;2}\theta_{*}}{c_{V0}\rho_{0}u_{*}^{2}}\langle R_{\alpha }\rho_{\alpha *}\Delta {\tilde{\rho }}_{\alpha }\rangle \\ +\left(\ell +1\right)\tau_{0}\frac{R_{0}{\hat{\theta }}_{0}}{c_{V0}}\left({\hat{u}}_{0}\cdot \nabla \right)\mathrm{div}\tilde{u}+\ell \tau_{0}{\left({\hat{u}}_{0}\cdot \nabla \right)}^{2}\tilde{\theta }+\left(\frac{\varkappa_{0}}{c_{V0}\rho_{0}u_{*}^{2}}+\tau_{0}\frac{R_{0}^{2}\theta_{0}}{c_{V0}u_{*}^{2}}\right)\Delta \tilde{\theta }\;\}\\ +\frac{{\hat{\theta }}_{0}\theta_{0}\rho_{\alpha *}}{c_{V0}\rho_{0}}{\langle d_{\alpha \beta 0}\left(\frac{R_{\alpha }}{\rho_{\alpha 0}}\Delta {\tilde{\rho }}_{\alpha }-\frac{R_{\beta }\rho_{\beta *}}{\rho_{\beta 0}\rho_{\alpha *}}\Delta {\tilde{\rho }}_{\beta }\right){\bar{e}}_{\alpha 0}\rangle }_{\alpha ,\beta }+\frac{\theta_{0}}{c_{V0}\rho_{0}}{\langle d_{\alpha \beta 0}\left({\bar{e}}_{\alpha 0}-{\bar{e}}_{\beta 0}\right){\bar{e}}_{\alpha 0}\rangle }_{\alpha ,\beta }\Delta \tilde{\theta }\}. \end{array}$$

Here, moreover, the scaling factors $u_{*}$ and $u_{*}^{2}$ are taken out of the convective and dissipative terms (i.e., the terms with the first and second order derivatives except for the diffusion terms), respectively, and the following notation is introduced for the components of the scaled background solution, background values of ρ, *R* and $c_{V}$ and the average value of $R_{\alpha }\gamma_{\alpha }$:

$$\begin{array}{c} {\hat{\rho }}_{\alpha 0}:=\frac{\rho_{\alpha 0}}{\rho_{\alpha *}},\;\;{\hat{u}}_{0}=\left({\hat{u}}_{10},\ldots ,{\hat{u}}_{n0}\right):=\frac{u_{0}}{u_{*}},\;\;{\hat{\theta }}_{0}:=\frac{\theta_{0}}{\theta_{*}},\\ \rho_{0}:=\langle \rho_{\alpha 0}\rangle ,\;\;R_{0}:=\langle \frac{\rho_{\alpha 0}}{\rho_{0}}R_{\alpha }\rangle ,\;\;c_{V0}:=\langle \frac{\rho_{\alpha 0}}{\rho_{0}}c_{V\alpha }\rangle ,\;\;{\left(R\gamma \right)}_{0}=\langle \frac{\rho_{\alpha 0}}{\rho_{0}}R_{\alpha }\gamma_{\alpha }\rangle . \end{array}$$

In addition, $d_{\alpha \beta 0}$, ${\bar{e}}_{\alpha 0}$, $\tau_{0}$, $\mu_{0}$, $\chi_{0}$ and $\varkappa_{0}$ are the values of $d_{\alpha \beta }$, ${\bar{e}}_{\alpha }$, τ, μ, χ and ϰ, respectively, on the background solution $z_{0}$, and the following PDE operator is involved

$${\left({\hat{u}}_{0}\cdot \nabla \right)}^{2}:=\left({\hat{u}}_{0}\cdot \nabla \right)\left({\hat{u}}_{0}\cdot \nabla \right)=\sum_{i,j=1}^{n}{\hat{u}}_{0i}{\hat{u}}_{0j}\partial_{i}\partial_{j}.$$

For $d_{1}=\ldots =d_{K}=0$, the possibility of simultaneous symmetrization of the convective and dissipative terms has recently been found in [35,37] by choosing the scaling parameters

(39) $$\begin{array}{c} \rho_{\alpha *}=b\sqrt{\frac{\rho_{\alpha 0}c_{V0}\rho_{0}}{R_{\alpha }}},\;\;\alpha =\bar{1,K},\;\;u_{*}=b\sqrt{c_{V0}\theta_{0}},\;\;\theta_{*}=b\theta_{0}\;\;\forall b>0, \end{array}$$

with a free parameter *b*. We accept this choice and pass to a much simpler form of the above linearized system of PDEs

(40) $$\begin{array}{c} \partial_{t}{\tilde{\rho }}_{\alpha }+u_{*}\left({\hat{u}}_{0}\cdot \nabla {\tilde{\rho }}_{\alpha }+{\hat{\rho }}_{\alpha 0}\mathrm{div}\tilde{u}\right)\\ =\tau_{0}u_{*}^{2}\left\{{\hat{\rho }}_{\alpha 0}{\langle {\hat{\rho }}_{\beta 0}\Delta {\tilde{\rho }}_{\beta }\rangle }_{\beta }+\ell {\left({\hat{u}}_{0}\cdot \nabla \right)}^{2}{\tilde{\rho }}_{\alpha }+\left(\ell +1\right){\hat{\rho }}_{\alpha 0}\left({\hat{u}}_{0}\cdot \nabla \right)\mathrm{div}\tilde{u}+a_{0}{\hat{\rho }}_{\alpha 0}\Delta \tilde{\theta }\;\right\}\\ +b_{\alpha }{\langle d_{\alpha \beta 0}\left(b_{\alpha }\Delta {\tilde{\rho }}_{\alpha }-b_{\beta }\Delta {\tilde{\rho }}_{\beta }\right)\rangle }_{\beta }+b_{\alpha }b_{\left(\theta \right)}{\langle d_{\alpha \beta 0}\left({\bar{e}}_{\alpha 0}-{\bar{e}}_{\beta 0}\right)\rangle }_{\beta }\Delta \tilde{\theta },\;\;\alpha =\bar{1,K}, \end{array}$$

(41) $$\begin{array}{c} \partial_{t}\tilde{u}+u_{*}\left(\langle {\hat{\rho }}_{\alpha 0}\nabla {\tilde{\rho }}_{\alpha }\rangle +\left({\hat{u}}_{0}\cdot \nabla \right)\tilde{u}+a_{0}\nabla \tilde{\theta }\;\right)=u_{*}^{2}\{\left(\ell +1\right)\tau_{0}\left({\hat{u}}_{0}\cdot \nabla \right)\langle {\hat{\rho }}_{\alpha 0}\nabla {\tilde{\rho }}_{\alpha }\rangle \\ +{\bar{\mu }}_{0}\Delta \tilde{u}+\left({\bar{\chi }}_{0}+\ell \tau_{0}{\hat{\theta }}_{0}{\left(a\gamma \right)}_{0}\right)\nabla \mathrm{div}\tilde{u}+\tau_{0}{\left({\hat{u}}_{0}\cdot \nabla \right)}^{2}\tilde{u}+\left(\ell +1\right)\tau_{0}a_{0}\left({\hat{u}}_{0}\cdot \nabla \right)\nabla \tilde{\theta }\;\}, \end{array}$$

(42) $$\begin{array}{c} \partial_{t}\tilde{\theta }+u_{*}\left(a_{0}\mathrm{div}\tilde{u}+{\hat{u}}_{0}\cdot \nabla \tilde{\theta }\;\right)\\ =u_{*}^{2}\left\{\tau_{0}a_{0}\langle {\hat{\rho }}_{\alpha 0}\Delta {\tilde{\rho }}_{\alpha }\rangle +\left(\ell +1\right)\tau_{0}a_{0}\left({\hat{u}}_{0}\cdot \nabla \right)\mathrm{div}\tilde{u}+\ell \tau_{0}{\left({\hat{u}}_{0}\cdot \nabla \right)}^{2}\tilde{\theta }+\left({\bar{\varkappa }}_{0}+\tau_{0}a_{0}^{2}\right)\Delta \tilde{\theta }\;\right\},\\ +b_{\left(\theta \right)}{\langle d_{\alpha \beta 0}\left(b_{\alpha }\Delta {\tilde{\rho }}_{\alpha }-b_{\beta }\Delta {\tilde{\rho }}_{\beta }\right){\bar{e}}_{\alpha 0}\rangle }_{\alpha ,\beta }+b_{\left(\theta \right)}^{2}{\langle d_{\alpha \beta 0}\left({\bar{e}}_{\alpha 0}-{\bar{e}}_{\beta 0}\right){\bar{e}}_{\alpha 0}\rangle }_{\alpha ,\beta }\Delta \tilde{\theta }\}. \end{array}$$

Here, the following constant factors have been introduced

$$\begin{array}{c} a_{\alpha }:=\frac{R_{\alpha }\theta_{*}}{u_{*}^{2}},\;\;a_{0}:=\langle \frac{\rho_{\alpha 0}}{\rho_{0}}a_{\alpha }\rangle =\frac{R_{0}\theta_{*}}{u_{*}^{2}},\;\;{\left(a\gamma \right)}_{0}=\langle \frac{\rho_{\alpha 0}}{\rho_{0}}a_{\alpha }\gamma_{\alpha }\rangle ,\;\;\\ b_{\alpha }:={\left(\frac{R_{\alpha }\theta_{0}}{\rho_{\alpha 0}}\right)}^{1/2},\;\;b_{\left(\theta \right)}:={\left(\frac{\theta_{0}}{c_{V0}\rho_{0}}\right)}^{1/2},\;\;{\bar{\mu }}_{0}:=\frac{\mu_{0}}{\rho_{0}u_{*}^{2}},\;\;{\bar{\chi }}_{0}:=\frac{\chi_{0}}{\rho_{0}u_{*}^{2}},\;\;{\bar{\varkappa }}_{0}:=\frac{\varkappa_{0}}{c_{V0}\rho_{0}u_{*}^{2}}, \end{array}$$

and, for the last two terms in (40) and (42), we have taken into account the formulas

$$\theta_{0}\frac{R_{\beta }\rho_{\beta *}}{\rho_{\beta 0}\rho_{\alpha *}}=b_{\alpha }b_{\beta },\;\;\frac{\theta_{*}}{\rho_{\alpha *}}=b_{\alpha }b_{\left(\theta \right)},\;\;\frac{{\hat{\theta }}_{0}\rho_{\alpha *}}{c_{V0}\rho_{0}}=\frac{b_{\left(\theta \right)}}{b_{\alpha }},\;\;\alpha ,\beta =\bar{1,K}.$$

Next, we study the initial-boundary value problem (IBVP) for the linearized system of PDEs (40)–(42) in the cylinder $Q_{\infty }:=\Omega \times \left(0,\infty \right)$ under the boundary and initial conditions

(43) $$\begin{array}{c} \tilde{z}{{|}_{\partial \Omega \times (0,\infty )}=0,\;\;\tilde{z}|}_{t=0}={\tilde{z}}^{\left(0\right)}\left(x\right). \end{array}$$

Let $L^{2}\left(\Omega \right)$ and $L^{2}\left(\Omega \right)$ be, respectively, the standard Lebesgue spaces of functions and vector functions defined on Ω and denote by ${\left(\cdot ,\cdot \right)}_{\Omega }={\left(\cdot ,\cdot \right)}_{L^{2}\left(\Omega \right)}$, ${∥\cdot ∥}_{\Omega }={∥\cdot ∥}_{L^{2}\left(\Omega \right)}$, ${\left(\cdot ,\cdot \right)}_{\Omega }={\left(\cdot ,\cdot \right)}_{L^{2}\left(\Omega \right)}$ and ${∥\cdot ∥}_{\Omega }={∥\cdot ∥}_{L^{2}\left(\Omega \right)}$ their inner products and norms. Let $H^{1}\left(\Omega \right)=W_{2}^{1}\left(\Omega \right)$ be a standard Sobolev space of vector functions defined on Ω, and $H_{0}^{1}\left(\Omega \right)$ be the closure in the $H^{1}\left(\Omega \right)$-norm of the space of smooth vector-functions with a compact support in Ω.

For $\partial_{t}\tilde{z}\left(\cdot ,t\right),\nabla \tilde{z}\left(\cdot ,t\right)\in L^{2}\left(\Omega \right)$, PDEs (40)–(42) correspond to the integral identity

(44) $$\begin{array}{c} {\left(\partial_{t}\tilde{z}\left(\cdot ,t\right),z\right)}_{\Omega }+u_{*}B_{\Omega }\left(\tilde{z}\left(\cdot ,t\right),z\right)+u_{*}^{2}A_{\Omega }\left(\tilde{z}\left(\cdot ,t\right),z\right)+A_{\Omega }^{d}\left(\tilde{z}\left(\cdot ,t\right),z\right)=0\;\;\forall z\in H_{0}^{1}\left(\Omega \right), \end{array}$$

for any vector-function $z=\left(\rho ,u,\theta \right)\left(x\right)\in H_{0}^{1}\left(\Omega \right)$ (which here is not the solution to the original quasilinear system of PDEs) and almost all t>0.

In the identity, the three bilinear forms are involved

$$\begin{array}{c} B_{\Omega }\left(\tilde{z},z\right):=\langle {\left({\hat{u}}_{0}\cdot \nabla {\tilde{\rho }}_{\alpha }+{\hat{\rho }}_{\alpha 0}\mathrm{div}\tilde{u},\rho_{\alpha }\right)}_{\Omega }\rangle \\ +{\left(\langle {\hat{\rho }}_{\alpha 0}\nabla {\tilde{\rho }}_{\alpha }\rangle +\left({\hat{u}}_{0}\cdot \nabla \right)\tilde{u}+a_{0}\nabla \tilde{\theta },u\right)}_{\Omega }+{\left(a_{0}\mathrm{div}\tilde{u}+{\hat{u}}_{0}\cdot \nabla \tilde{\theta },\theta \right)}_{\Omega },\\ A_{\Omega }\left(\tilde{z},z\right):={\bar{\mu }}_{0}{\left(\nabla \tilde{u},\nabla u\right)}_{\Omega }+{\bar{\chi }}_{0}{\left(\mathrm{div}\tilde{u},\mathrm{div}u\right)}_{\Omega }+{\bar{\varkappa }}_{0}{\left(\nabla \tilde{\theta },\nabla \theta \right)}_{\Omega }\\ +\tau_{0}\{{\left(\langle {\hat{\rho }}_{\alpha 0}\nabla {\tilde{\rho }}_{\alpha }\rangle ,\langle {\hat{\rho }}_{\alpha 0}\nabla \rho_{\alpha }\rangle \right)}_{\Omega }+\ell \langle {\left({\hat{u}}_{0}\cdot \nabla {\tilde{\rho }}_{\alpha },\left({\hat{u}}_{0}\cdot \nabla \right)\rho_{\alpha }\right)}_{\Omega }\rangle +\left(\ell +1\right){\left(\left({\hat{u}}_{0}\cdot \nabla \right)\tilde{u},\langle {\hat{\rho }}_{\alpha 0}\nabla \rho_{\alpha }\rangle \right)}_{\Omega }\\ +{\left(a_{0}\nabla \tilde{\theta },\langle {\hat{\rho }}_{\alpha 0}\nabla \rho_{\alpha }\rangle \right)}_{\Omega }+\left(\ell +1\right){\left(\langle {\hat{\rho }}_{\alpha 0}\nabla {\tilde{\rho }}_{\alpha }\rangle ,\left({\hat{u}}_{0}\cdot \nabla \right)u\right)}_{\Omega }+\ell {\left({\hat{\theta }}_{0}{\left(a\gamma \right)}_{0}\mathrm{div}\tilde{u},\mathrm{div}u\right)}_{\Omega }\\ +{\left(\left({\hat{u}}_{0}\cdot \nabla \right)\tilde{u},\left({\hat{u}}_{0}\cdot \nabla \right)u\right)}_{\Omega }+\left(\ell +1\right){\left(a_{0}\nabla \tilde{\theta },\left({\hat{u}}_{0}\cdot \nabla \right)u\right)}_{\Omega }\\ +{\left(\langle {\hat{\rho }}_{\alpha 0}\nabla {\tilde{\rho }}_{\alpha }\rangle ,a_{0}\nabla \theta \right)}_{\Omega }+\left(\ell +1\right){\left(\left({\hat{u}}_{0}\cdot \nabla \right)\tilde{u},a_{0}\nabla \theta \right)}_{\Omega }+\ell {\left({\hat{u}}_{0}\cdot \nabla \tilde{\theta },{\hat{u}}_{0}\cdot \nabla \theta \right)}_{\Omega }+{\left(a_{0}\nabla \tilde{\theta },a_{0}\nabla \theta \right)}_{\Omega }\}, \end{array}$$

where the tensors $\nabla \tilde{u}$ and ∇u are considered as vectors of length $n^{2}$, and

$$\begin{array}{c} A_{\Omega }^{d}\left(\tilde{z},z\right):={\langle {\left(d_{\alpha \beta 0}\left(b_{\alpha }\nabla {\tilde{\rho }}_{\alpha }-b_{\beta }\nabla {\tilde{\rho }}_{\beta }\right),b_{\alpha }\nabla \rho_{\alpha }\right)}_{\Omega }\rangle }_{\alpha ,\beta }+{\langle {\left(d_{\alpha \beta 0}b_{\left(\theta \right)}\left({\bar{e}}_{\alpha 0}-{\bar{e}}_{\beta 0}\right)\nabla \tilde{\theta },b_{\alpha }\nabla \rho_{\alpha }\right)}_{\Omega }\rangle }_{\alpha ,\beta }\\ +{\langle {\left(d_{\alpha \beta 0}\left(b_{\alpha }\nabla {\tilde{\rho }}_{\alpha }-b_{\beta }\nabla {\tilde{\rho }}_{\beta }\right),b_{\left(\theta \right)}{\bar{e}}_{\alpha 0}\nabla \theta \right)}_{\Omega }\rangle }_{\alpha ,\beta }+{\langle {\left(d_{\alpha \beta 0}b_{\left(\theta \right)}\left({\bar{e}}_{\alpha 0}-{\bar{e}}_{\beta 0}\right)\nabla \tilde{\theta },b_{\left(\theta \right)}{\bar{e}}_{\alpha 0}\nabla \theta \right)}_{\Omega }\rangle }_{\alpha ,\beta }. \end{array}$$

The last bilinear form corresponds to the diffusive terms and is independent of $\tilde{u}$ and u. Formally, identity (44) arises after multiplying Equations (40)–(42) by $\rho_{\alpha }$, u and θ, respectively, integrating over Ω and by parts and summing up the results.

Let us study properties of the defined bilinear forms that is crucial below.

 **Lemma 2.** *Let $d_{\alpha \beta 0}=d_{\beta \alpha 0}$ for any α≠β. The following skew symmetry and symmetry properties hold*

(45) $$\begin{array}{c} B_{\Omega }\left(\tilde{z},z\right)=-B_{\Omega }\left(z,\tilde{z}\right)\;\;\forall \tilde{z}\in H^{1}\left(\Omega \right),z\in H_{0}^{1}\left(\Omega \right), \end{array}$$

(46) $$\begin{array}{c} A_{\Omega }\left(\tilde{z},z\right)=A_{\Omega }\left(z,\tilde{z}\right),\;\;A_{\Omega }^{d}\left(\tilde{z},z\right)=A_{\Omega }^{d}\left(z,\tilde{z}\right)\;\;\forall \;\tilde{z},z\in H^{1}\left(\Omega \right). \end{array}$$

*Moreover, let $z=\left(\rho ,u,\theta \right)\in H^{1}\left(\Omega \right)$ with $u\in H_{0}^{1}\left(\Omega \right)$. The following representations for the quadratic forms hold*

(47) $$\begin{array}{c} A_{\Omega }\left(z,z\right)={\bar{\mu }}_{0}{∥\nabla u∥}_{\Omega }^{2}+{\bar{\chi }}_{0}{∥\mathrm{div}u∥}_{\Omega }^{2}+{\bar{\varkappa }}_{0}{∥\nabla \theta ∥}_{\Omega }^{2}+\tau_{0}{∥\langle {\hat{\rho }}_{\alpha 0}\nabla \rho_{\alpha }\rangle +\left({\hat{u}}_{0}\cdot \nabla \right)u+a_{0}\nabla \theta ∥}_{\Omega }^{2}\\ +\ell \tau_{0}\left(\langle ∥{\hat{u}}_{0}\cdot \nabla \rho_{\alpha }+{\hat{\rho }}_{\alpha 0}{\mathrm{div}u∥}_{\Omega }^{2}\rangle +∥a_{0}\mathrm{div}u+{\hat{u}}_{0}\cdot \nabla \theta {∥}_{\Omega }^{2}+g_{0}{∥\mathrm{div}u∥}_{\Omega }^{2}\right), \end{array}$$

(48) $$\begin{array}{c} A_{\Omega }^{d}\left(z,z\right)=\frac{1}{2}{\langle d_{\alpha \beta 0}{∥b_{\alpha }\nabla \rho_{\alpha }-b_{\beta }\nabla \rho_{\beta }+b_{\left(\theta \right)}\left({\bar{e}}_{\alpha 0}-{\bar{e}}_{\beta 0}\right)\nabla \theta ∥}_{\Omega }^{2}\rangle }_{\alpha ,\beta }, \end{array}$$

with $g_{0}:=\frac{1}{u_{*}^{2}}\left(\langle \gamma_{\alpha }p_{\alpha 0}\rangle -\gamma_{0}p_{0}\right)⩾0$, see relations (22), where $p_{\alpha 0}$, $\gamma_{0}$ and $p_{0}$ are the values of $p_{\alpha }$, γ and p on the background solution $z_{0}$.

 **Proof.**  To prove the skew symmetry property (45), we integrate by parts term by term in the definition of $B_{\Omega }\left(\tilde{z},z\right)$, rearrange the summands and obtain the equalities

$$\begin{array}{c} B_{\Omega }\left(\tilde{z},z\right)=-\langle {\left({\tilde{\rho }}_{\alpha },{\hat{u}}_{0}\cdot \nabla \rho_{\alpha }\right)}_{\Omega }\rangle -(\tilde{u},\langle {\hat{\rho }}_{\alpha 0}\nabla \rho_{\alpha }\rangle {)}_{\Omega }-\langle {\left({\tilde{\rho }}_{\alpha },{\hat{\rho }}_{\alpha 0}\mathrm{div}u\right)}_{\Omega }\rangle -{\left(\tilde{u},\left({\hat{u}}_{0}\cdot \nabla \right)u\right)}_{\Omega }\\ -{\left(\tilde{\theta },a_{0}\mathrm{div}u\right)}_{\Omega }-(\tilde{u},a_{0}{\nabla \theta )}_{\Omega }-{\left(\tilde{\theta },{\hat{u}}_{0}\cdot \nabla \theta \right)}_{\Omega }=-B_{\Omega }\left(z,\tilde{z}\right). \end{array}$$

The first symmetry property (46) is obvious. Due to identity (16) applied to each term, the following formulas hold

$$\begin{array}{c} 2A_{\Omega }^{d}\left(\tilde{z},z\right)={\langle {\left(d_{\alpha \beta 0}\left(b_{\alpha }\nabla {\tilde{\rho }}_{\alpha }-b_{\beta }\nabla {\tilde{\rho }}_{\beta }\right),b_{\alpha }\nabla \rho_{\alpha }-b_{\beta }\nabla \rho_{\beta }\right)}_{\Omega }\rangle }_{\alpha ,\beta }\\ +{\langle {\left(d_{\alpha \beta 0}b_{\left(\theta \right)}\left({\bar{e}}_{\alpha 0}-{\bar{e}}_{\beta 0}\right)\nabla \tilde{\theta },b_{\alpha }\nabla \rho_{\alpha }-b_{\beta }\nabla \rho_{\beta }\right)}_{\Omega }\rangle }_{\alpha ,\beta }\\ +{\langle {\left(d_{\alpha \beta 0}\left(b_{\alpha }\nabla {\tilde{\rho }}_{\alpha }-b_{\beta }\nabla {\tilde{\rho }}_{\beta }\right),b_{\left(\theta \right)}\left({\bar{e}}_{\alpha 0}-{\bar{e}}_{\beta 0}\right)\nabla \theta \right)}_{\Omega }\rangle }_{\alpha ,\beta }\\ +{\langle {\left(d_{\alpha \beta 0}b_{\left(\theta \right)}\left({\bar{e}}_{\alpha 0}-{\bar{e}}_{\beta 0}\right)\nabla \tilde{\theta },b_{\left(\theta \right)}\left({\bar{e}}_{\alpha 0}-{\bar{e}}_{\beta 0}\right)\nabla \theta \right)}_{\Omega }\rangle }_{\alpha ,\beta }\\ ={\langle {\left(d_{\alpha \beta 0}\left(b_{\alpha }\nabla {\tilde{\rho }}_{\alpha }-b_{\beta }\nabla {\tilde{\rho }}_{\beta }+b_{\left(\theta \right)}\left({\bar{e}}_{\alpha 0}-{\bar{e}}_{\beta 0}\right)\nabla \tilde{\theta }\;\right),b_{\alpha }\nabla \rho_{\alpha }-b_{\beta }\nabla \rho_{\beta }+b_{\left(\theta \right)}\left({\bar{e}}_{\alpha 0}-{\bar{e}}_{\beta 0}\right)\nabla \theta \right)}_{\Omega }\rangle }_{\alpha ,\beta }. \end{array}$$

They imply the symmetry property (46) for $A_{\Omega }^{d}\left(\tilde{z},z\right)$ and representation (48) for $A_{\Omega }^{d}\left(z,z\right)$.

Property (47) for ℓ=0 has recently been checked in [37] (for $\Omega =R^{n}$ that is not essential). The rest of the terms in $A_{\Omega }\left(z,z\right)$ are as follows

$$\begin{array}{c} \ell \tau_{0}\{\langle ∥{\hat{u}}_{0}\cdot \nabla \rho_{\alpha }{∥}_{\Omega }^{2}\rangle +2{\left(\langle {\hat{\rho }}_{\alpha 0}\nabla \rho_{\alpha }\rangle ,\left({\hat{u}}_{0}\cdot \nabla \right)u\right)}_{\Omega }+2{\left(\left({\hat{u}}_{0}\cdot \nabla \right)u,a_{0}\nabla \theta \right)}_{\Omega }\\ +{\hat{\theta }}_{0}{\left(a\gamma \right)}_{0}{∥\mathrm{div}u∥}_{\Omega }^{2}+{∥{\hat{u}}_{0}\cdot \nabla \theta ∥}_{\Omega }^{2}\}=:A_{\ell \Omega }\left(z,z\right). \end{array}$$

Next, we recall the following algebraic formula and integral identity

(49) $$\begin{array}{c} \langle {\left({\hat{u}}_{0}\cdot \nabla \rho_{\alpha }+{\hat{\rho }}_{\alpha 0}\mathrm{div}u\right)}^{2}\rangle =\langle {\left({\hat{u}}_{0}\cdot \nabla \rho_{\alpha }\right)}^{2}\rangle \\ +\left(\langle {\hat{\rho }}_{\alpha 0}^{\;2}\rangle +a_{0}^{2}\right){\left(\mathrm{div}u\right)}^{2}+{\left({\hat{u}}_{0}\cdot \nabla \theta \right)}^{2}+2\left({\hat{u}}_{0}\cdot \langle {\hat{\rho }}_{\alpha 0}\nabla \rho_{\alpha }\rangle \right)\mathrm{div}u+2a_{0}\left({\hat{u}}_{0}\cdot \nabla \theta \right)\mathrm{div}u,\\ {\left({\hat{u}}_{0}\cdot \nabla \varphi ,\mathrm{div}u\right)}_{\Omega }={\left(\nabla \varphi ,\left({\hat{u}}_{0}\cdot \nabla \right)u\right)}_{\Omega }\;\;\forall \varphi \in H^{1}\left(\Omega \right),u\in H_{0}^{1}\left(\Omega \right), \end{array}$$

see ([35], formulas (4.14) and (4.18)). Integrating Formula (49) over Ω and applying the last identity, we find

$$A_{\ell \Omega }\left(z,z\right)=\ell \tau_{0}\left(\langle ∥{\hat{u}}_{0}\cdot \nabla \rho_{\alpha }+{\hat{\rho }}_{\alpha 0}{\mathrm{div}u∥}_{\Omega }^{2}\rangle +∥a_{0}\mathrm{div}u+{\hat{u}}_{0}\cdot \nabla \theta {∥}_{\Omega }^{2}+g_{0}{∥\mathrm{div}u∥}_{\Omega }^{2}\right)$$

with $g_{0}:={\hat{\theta }}_{0}{\left(a\gamma \right)}_{0}-\left(\langle {\hat{\rho }}_{\alpha 0}^{\;2}\rangle +a_{0}^{2}\right)$. According to ([35], proof of Lemma 3.1) and relations (22), we obtain $g_{0}=\frac{1}{u_{*}^{2}}\left(\langle \gamma_{\alpha }p_{\alpha 0}\rangle -\gamma_{0}p_{0}\right)⩾0$ that completes the proof of representation (47). □

 **Corollary 1.** *Let $d_{\alpha \beta 0}=d_{\beta \alpha 0}⩾d>0$ for any α≠β. The following positive definiteness inequality holds*

(50) $$\begin{array}{c} u_{*}^{2}A_{\Omega }\left(z,z\right)+A_{\Omega }^{d}\left(z,z\right)⩾\max\left\{\delta_{1}\langle ∥\nabla \rho_{\alpha }{∥}_{\Omega }^{2}\rangle ,\delta_{0}\left({∥\nabla u∥}_{\Omega }^{2}+{∥\nabla \theta ∥}_{\Omega }^{2}\right)\right\}, \end{array}$$

for any $z=\left(\rho ,u,\theta \right)\in H^{1}\left(\Omega \right)$ with $u\in H_{0}^{1}\left(\Omega \right)$, with $\delta_{0}:=u_{*}^{2}\min\left\{{\bar{\mu }}_{0},{\bar{\varkappa }}_{0}\right\}>0$ and some $\delta_{1}>0$.

 **Proof.**  Clearly, representations (47) and (48) imply the lower bound

$$\begin{array}{c} u_{*}^{2}A_{\Omega }\left(z,z\right)+A_{\Omega }^{d}\left(z,z\right)⩾u_{*}^{2}\left({\bar{\mu }}_{0}{∥\nabla u∥}_{\Omega }^{2}+{\bar{\chi }}_{0}{∥\mathrm{div}u∥}_{\Omega }^{2}+{\bar{\varkappa }}_{0}{∥\nabla \theta ∥}_{\Omega }^{2}\right)\\ +u_{*}^{2}\tau_{0}{∥\langle {\hat{\rho }}_{\alpha 0}\nabla \rho_{\alpha }\rangle +\left({\hat{u}}_{0}\cdot \nabla \right)u+a_{0}\nabla \theta ∥}_{\Omega }^{2}+\frac{1}{2}d{\langle ∥b_{\alpha }\nabla \rho_{\alpha }-b_{\beta }\nabla \rho_{\beta }+b_{\left(\theta \right)}\left({\bar{e}}_{\alpha 0}-{\bar{e}}_{\beta 0}\right){\nabla \theta ∥}_{\Omega }^{2}\rangle }_{\alpha ,\beta }. \end{array}$$

We further apply simple bounds for the terms containing $\nabla \rho_{\alpha }$:

$$\begin{array}{c} ∥\langle {\hat{\rho }}_{\alpha 0}\nabla \rho_{\alpha }\rangle {∥}_{\Omega }^{2}⩽2\left(∥\langle {\hat{\rho }}_{\alpha 0}\nabla \rho_{\alpha }\rangle +\left({\hat{u}}_{0}\cdot \nabla \right)u+a_{0}{\nabla \theta ∥}_{\Omega }^{2}+2|{\hat{u}}_{0}{|}^{2}{∥\nabla u∥}_{\Omega }^{2}+a_{0}^{2}{∥\nabla \theta ∥}_{\Omega }^{2}\right),\\ ∥b_{\alpha }\nabla \rho_{\alpha }-b_{\beta }\nabla \rho_{\beta }{∥}_{\Omega }^{2}⩽2∥b_{\alpha }\nabla \rho_{\alpha }-b_{\beta }\nabla \rho_{\beta }+b_{\left(\theta \right)}\left({\bar{e}}_{\alpha 0}-{\bar{e}}_{\beta 0}\right){\nabla \theta ∥}_{\Omega }^{2}+2b_{\left(\theta \right)}^{2}{\left({\bar{e}}_{\alpha 0}-{\bar{e}}_{\beta 0}\right)}^{2}{∥\nabla \theta ∥}_{\Omega }^{2} \end{array}$$

and

$$\begin{array}{c} b_{\alpha }^{2}{∥\nabla \rho_{\alpha }∥}_{\Omega }^{2}⩽2\left(∥b_{\alpha }\nabla \rho_{\alpha }-\frac{1}{{\langle {\overset{ˇ}{\rho }}_{\beta 0}\rangle }_{\beta }}{\langle {\hat{\rho }}_{\beta 0}\nabla \rho_{\beta }\rangle }_{\beta }{∥}_{\Omega }^{2}+∥\frac{1}{{\langle {\overset{ˇ}{\rho }}_{\beta 0}\rangle }_{\beta }}{\langle {\hat{\rho }}_{\beta 0}\nabla \rho_{\beta }\rangle }_{\beta }{∥}_{\Omega }^{2}\right)\\ =\frac{2}{{\langle {\overset{ˇ}{\rho }}_{\beta 0}\rangle }_{\beta }^{2}}\left(∥{\langle {\overset{ˇ}{\rho }}_{\beta 0}\left(b_{\alpha }\nabla \rho_{\alpha }-b_{\beta }\nabla \rho_{\beta }\right)\rangle }_{\beta }{∥}_{\Omega }^{2}+{∥{\langle {\hat{\rho }}_{\beta 0}\nabla \rho_{\beta }\rangle }_{\beta }∥}_{\Omega }^{2}\right)\\ ⩽\frac{2}{{\langle {\overset{ˇ}{\rho }}_{\beta 0}\rangle }_{\beta }^{2}}\left({\langle {\overset{ˇ}{\rho }}_{\beta 0}^{2}\rangle }_{\beta }\langle ∥{(b_{\alpha }\nabla \rho_{\alpha }-b_{\beta }\nabla \rho_{\beta }{∥}_{\Omega }^{2}\rangle }_{\beta }+{∥{\langle {\hat{\rho }}_{\beta 0}\nabla \rho_{\beta }\rangle }_{\beta }∥}_{\Omega }^{2}\right), \end{array}$$

where ${\overset{ˇ}{\rho }}_{\beta 0}:={\hat{\rho }}_{\beta 0}/b_{\beta }$. According to these bounds, we derive

$$\begin{array}{c} \langle ∥\nabla \rho_{\alpha }{∥}_{\Omega }^{2}\rangle ⩽\frac{2}{{\langle {\overset{ˇ}{\rho }}_{\beta 0}\rangle }_{\beta }^{2}\min_{\alpha =\bar{1,K}}b_{\alpha }^{2}}\left({\langle {\overset{ˇ}{\rho }}_{\beta 0}^{2}\rangle }_{\beta }\langle ∥{(b_{\alpha }\nabla \rho_{\alpha }-b_{\beta }\nabla \rho_{\beta }{∥}_{\Omega }^{2}\rangle }_{\alpha ,\beta }+K{∥\langle {\hat{\rho }}_{\alpha 0}\nabla \rho_{\alpha }\rangle ∥}_{\Omega }^{2}\right)\\ ⩽{\tilde{\delta }}_{1}{(∥\nabla u∥}_{\Omega }^{2}+{∥\nabla \theta ∥}_{\Omega }^{2}+{∥\langle {\hat{\rho }}_{\alpha 0}\nabla \rho_{\alpha }\rangle +\left({\hat{u}}_{0}\cdot \nabla \right)u+a_{0}\nabla \theta ∥}_{\Omega }^{2}\\ +{\langle ∥b_{\alpha }\nabla \rho_{\alpha }-b_{\beta }\nabla \rho_{\beta }+b_{\left(\theta \right)}\left({\bar{e}}_{\alpha 0}-{\bar{e}}_{\beta 0}\right){\nabla \theta ∥}_{\Omega }^{2}\rangle }_{\alpha ,\beta }), \end{array}$$

with ${\tilde{\delta }}_{1}$ depending on ${\hat{\rho }}_{\alpha 0}$, $|{\hat{u}}_{0}|$, ${\bar{e}}_{\alpha 0}$ ($\alpha =\bar{1,K}$), $a_{0}$, $b_{\alpha }$ and $b_{\left(\theta \right)}$. This estimate and the above lower bound imply the positive definiteness inequality (50). □

Let Ω be a bounded domain in $R^{n}$. Define the dual space $H^{-1}\left(\Omega \right)={\left(H_{0}^{1}\left(\Omega \right)\right)}^{*}$ and the duality relation ${\langle \cdot ,\cdot \rangle }_{\Omega }$ on $H^{-1}\left(\Omega \right)\times H_{0}^{1}\left(\Omega \right)$. Denote by $V(Q_{T})$, with $Q_{T}=\Omega \times \left(0,T\right)$, the space of vector functions $\tilde{z}\in L^{2}\left(\left(0,T\right);H_{0}^{1}\left(\Omega \right)\right)$ possessing a distributional derivative $\partial_{t}\tilde{z}\in L^{2}\left(\left(0,T\right);H^{-1}\left(\Omega \right)\right)$, see these notions, for example, in [56].

We define the weak solution $\tilde{z}\in V\left(Q_{T}\right)$, for any T>0, to the IBVP for the system of PDEs (40)–(42) in $Q_{\infty }$ together with conditions (43), such that the integral identity

(51) $$\begin{array}{c} \int_{0}^{T}{\langle \partial_{t}\tilde{z}\left(\cdot ,t\right),z\left(\cdot ,t\right)\rangle }_{\Omega }\;dt+u_{*}B_{Q_{T}}\left(\tilde{z},z\right)+u_{*}^{2}A_{Q_{T}}\left(\tilde{z},z\right)+A_{Q_{T}}^{d}\left(\tilde{z},z\right)=0, \end{array}$$

for any $z\in L^{2}\left(\left(0,T\right);H_{0}^{1}\left(\Omega \right)\right)$ and any T>0, together with the initial condition $\tilde{z}{|}_{t=0}={\tilde{z}}^{\left(0\right)}\in L^{2}\left(\Omega \right)$ are valid. Here, the inner products in the bilinear forms $B_{Q_{T}}$, $A_{Q_{T}}$ and $A_{Q_{T}}^{d}$ are taken over $Q_{T}$ instead of Ω as originally. Due to the well known embedding $V\left(Q_{T}\right)\subset C\left(\left[0,T\right];L^{2}\left(\Omega \right)\right)$ [56], the initial condition is understood by continuity in $L^{2}\left(\Omega \right)$. Formally, identity (51) arises from the previous one (44) for z=z(·,t) by integration over (0,T).

Now, we are ready to state the second main result.

 **Theorem 2.** *Let $d_{\alpha \beta 0}=d_{\beta \alpha 0}⩾d>0$ for any α≠β. The defined weak solution $\tilde{z}\in V\left(Q_{T}\right)$, for any T>0, to the IBVP (40)–(43) for the linearized system of PDEs exists and is unique. It satisfies the energy equality and bound*

$$\begin{array}{c} \frac{1}{2}∥\tilde{z}{\left(\cdot ,T\right)∥}_{L^{2}\left(\Omega \right)}^{2}+u_{*}^{2}A_{Q_{T}}\left(\tilde{z},\tilde{z}\right)+A_{Q_{T}}^{d}\left(\tilde{z},\tilde{z}\right)=\frac{1}{2}{∥{\tilde{z}}^{\left(0\right)}∥}_{L^{2}\left(\Omega \right)}^{2}\;\;\forall T>0,\\ \max\{\max_{t⩾0}{∥\tilde{z}\left(\cdot ,t\right)∥}_{L^{2}\left(\Omega \right)},\;\sqrt{2\delta_{1}}{\langle ∥\nabla {\tilde{\rho }}_{\alpha }{∥}_{L^{2}\left(Q\right)}^{2}\rangle }^{1/2},\\ \sqrt{2}u_{*}{\left({\bar{\mu }}_{0}{∥\nabla u∥}_{L^{2}\left(Q\right)}^{2}+{\bar{\chi }}_{0}{∥\mathrm{div}u∥}_{L^{2}\left(Q\right)}^{2}+{\bar{\varkappa }}_{0}{∥\nabla \theta ∥}_{L^{2}\left(Q\right)}^{2}\right)}^{1/2}\}⩽{∥{\tilde{z}}^{\left(0\right)}∥}_{L^{2}\left(\Omega \right)}. \end{array}$$

*In addition, the derivative $\partial_{t}\left(∥\tilde{z}{\left(\cdot ,t\right)∥}_{L^{2}\left(\Omega \right)}^{2}\right)\in L^{1}\left(0,\infty \right)$ exists, and another form of the energy equality holds*

(52) $$\begin{array}{c} \frac{1}{2}\partial_{t}(∥\tilde{z}\left(\cdot ,t\right){∥}_{L^{2}\left(\Omega \right)}^{2})+u_{*}^{2}A_{\Omega }\left(\tilde{z}\left(\cdot ,t\right),\tilde{z}\left(\cdot ,t\right)\right)+A_{\Omega }^{d}\left(\tilde{z}\left(\cdot ,t\right),\tilde{z}\left(\cdot ,t\right)\right)=0 \end{array}$$

*for almost all t>0 and, consequently, the following strong $L^{2}\left(\Omega \right)$-dissipativity property holds*

$$\partial_{t}\left(∥\tilde{z}{\left(\cdot ,t\right)∥}_{L^{2}\left(\Omega \right)}^{2}\right)⩽0\;\;\mathrm{for}\;\mathrm{almost}\;\mathrm{all}\;\;t>0.$$

The proof is based on some general results from [56] together with Lemma 2 and Corollary 1 and is quite similar to that of ([35], Theorem 3.2 and Corollary 3.1), so we omit it. In the case d=0, the Cauchy problem can be considered similarly to ([37], Theorem 2).

Notice that the exponential decay $∥\tilde{z}{\left(\cdot ,t\right)∥}_{L^{2}\left(\Omega \right)}⩽e^{-\delta_{2}t}{∥{\tilde{z}}^{\left(0\right)}∥}_{L^{2}\left(\Omega \right)}$ for t⩾0, with $\delta_{2}=\frac{1}{2}\left(\delta_{0}+\delta_{1}\right)>0$, follows from the energy equality (52) and Corollary 1. Methods developed in theory of linear parabolic PDEs (see, for example, references [56,57,58]) allow one to derive various regularity properties for $\tilde{z}$ which we do not consider here.

Lemmas 1 and 2 and Corollary 1 lead also to other important corollaries which we briefly describe now. First, it can be checked that the system of PDEs (1), (36) and (37) is uniformly parabolic in the Petrovskii sense [58] in any bounded subdomain $D\subset D_{+}:={\left(0,\infty \right)}^{K}\times R^{n}\times \left(0,\infty \right)$ of values of its solutions z=(ρ,u,θ) under additional assumptions on the diffusion fluxes $d_{\alpha \beta }=d_{\beta \alpha }>0$ and $d_{\alpha \beta },e_{\alpha }\in C^{2}\left(D_{+}\right)$ for all α≠β.

Due to the equivalence (for classical solutions) between system of PDEs (1), (36) and (37) and the original quasilinear system of PDEs (1)–(3) for multicomponent gas dynamics introduced in Section 2 and some very general results from [58], only this Petrovskii parabolicity property implies the local-in-time classical (in anisotropic Hölder spaces) unique solvability of the Cauchy problem to the latter system. Its statement is identical to that of the corresponding ([35], Theorem 3.3). Moreover, the proof of the Petrovskii parabolicity property can be made very close as well, using the remark from ([35], proof of Lemma 3.2) that the uniform in D positive definiteness of a matrix $A(z_{0},\xi )$, defining the property is equivalent to a result such as Corollary 1 in the case $\Omega =R^{n}$, which goes back to a technique using the integral Fourier transform from ([17], Section 3) (where the Petrovskii parabolicity was studied in the single-component gas case). Notice that it was shown in [35] that the matrix $A(z_{0},\xi )$ can be symmetrized by the same scaling as accomplished above for passing to the linearized system of PDEs (40)–(42), and thus this symmetry property is directly connected to the symmetry of the bilinear forms in Lemma 2. For brevity, here we omit details of both the statement and proof of such theorem.

In addition, the Petrovskii parabolicity property allows one to pose correctly some simple boundary conditions in IBVPs for the original system of PDEs, similarly to the single-component case. We emphasize that the presence of the diffusion fluxes is crucial for validity of this property, for without them, the original system of PDEs becomes more complicated composite hyperbolic–parabolic, not parabolic. For ℓ=0, this has recently been checked in detail in [37]. The reason is that the quadratic form $A_{\Omega }\left(z,z\right)$ is only non-negative rather than positive definite in $H_{0}^{1}\left(\Omega \right)$, and the degeneration occurs with respect to ∇ρ. For ℓ=1, this can be performed similarly and, moreover, this is clear if D contains a point $z_{0}=\left(\rho_{0},0,\theta_{0}\right)$, since then the quadratic form $A_{\Omega }\left(z,z\right)$ is identical for ℓ=0 and 1 at such point $z_{0}$, with ${\bar{\chi }}_{0}+\tau_{0}a^{2}$ substituted for ${\bar{\chi }}_{0}$. For systems of composite hyperbolic–parabolic type, results of type ([35], Theorem 3.3) are not valid any more, and the statement of correct boundary conditions in IBVPs for the original system of PDEs becomes more complicated and has not yet been studied.

## 4. The 1D Regularized System of PDEs for Gas Mixture Dynamics and Its Entropy Correct Spatial Discretization

Starting from this section, we pass to the particular 1D case of the above regularized system of PDEs for the gas mixture dynamics, and its constituent balance PDEs for the mass of the components and the momentum and total energy of the mixture take a simpler form

(53) $$\begin{array}{c} \partial_{t}\rho_{\alpha }+\partial_{x}\left(\rho_{\alpha }\left(u-w_{\ell \alpha }\right)+d_{\alpha }\right)=0,\;\;\alpha =\bar{1,K}, \end{array}$$

(54) $$\begin{array}{c} \partial_{t}\left(\rho u\right)+\partial_{x}\left(\langle \rho_{\alpha }\left(u-w_{\ell \alpha }\right)\rangle u+p\right)=\partial_{x}\Pi_{\ell }+\left(\rho -\ell \tau \partial_{x}\left(\rho u\right)\right)f, \end{array}$$

(55) $$\begin{array}{c} \partial_{t}E+\partial_{x}\langle \left(E_{\alpha }+p_{\alpha }\right)\left(u-w_{\ell \alpha }\right)\rangle =\partial_{x}\left(-q_{\ell }+\Pi_{\ell }u\right)+\langle \rho_{\alpha }\left(u-w_{\ell \alpha }\right)\rangle f+Q, \end{array}$$

with ℓ=0,1. The main sought functions are the component densities $\rho_{1}>0,\ldots ,\rho_{K}>0$ and their common velocity and temperature *u* and θ>0 depending on (x,t)∈[−X,X]×[0,T]. We exploit the previous Formulas (4)–(6) for the main gas state variables and the total energy of the components and the mixture, where now ${\left|u\right|}^{2}=u^{2}$.

The 1D formulas for the regularizing velocities, viscous stress and heat flux look as follows

(56) $$\begin{array}{c} w_{\ell \alpha }=\ell \frac{\tau }{\rho_{\alpha }}u\partial_{x}\left(\rho_{\alpha }u\right)+\hat{w},\;\;\hat{w}=\frac{\tau }{\rho }\left(\rho u\partial_{x}u+\partial_{x}p-\rho f\right),\;\; \end{array}$$

(57) $$\begin{array}{c} \Pi_{\ell }=\nu \partial_{x}u+\Pi_{\ell }^{\tau },\;\;\Pi_{\ell }^{\tau }=\rho u\hat{w}+\ell \tau \left\{u\partial_{x}p+\langle \gamma_{\alpha }p_{\alpha }\rangle \partial_{x}u-\langle \left(\gamma_{\alpha }-1\right)Q_{\alpha }\rangle \right\}, \end{array}$$

(58) $$\begin{array}{c} q_{\ell }=-\varkappa \partial_{x}\theta +\ell q^{\tau }+q^{d},\;\; \end{array}$$

(59) $$\begin{array}{c} -q^{\tau }=\tau \left\{\left(\langle c_{V\alpha }\rho_{\alpha }\rangle \partial_{x}\theta -\theta \partial_{x}\langle R_{\alpha }\rho_{\alpha }\rangle \right)u^{2}-Qu\right\}=\tau \left\{\left(c_{V}\rho \partial_{x}\theta -\theta \partial_{x}\left(R\rho \right)\right)u^{2}-Qu\right\}, \end{array}$$

(60) $$\begin{array}{c} -d_{\alpha }={\langle d_{\alpha \beta }\left(\partial_{x}\left(G_{\alpha }-G_{\beta }\right)+\left(e_{\alpha }-e_{\beta }\right)\partial_{x}\theta \right)\rangle }_{\beta },\;\;q^{d}=\langle \left(G_{\alpha }+e_{\alpha }\theta \right)d_{\alpha }\rangle , \end{array}$$

where $\nu :=\frac{4}{3}\mu +\lambda$ and $\alpha =\bar{1,K}$.

As above, introducing the average regularizing velocity $w_{\ell }:=\langle \frac{\rho_{\alpha }}{\rho }w_{\ell \alpha }\rangle =\ell \frac{\tau }{\rho }u\partial_{x}\left(\rho u\right)+\hat{w}$ allows one to simplify the form of the balance PDEs for the momentum and total energy of the mixture (54) and (55):

(61) $$\begin{array}{c} \partial_{t}\left(\rho u\right)+\partial_{x}\left(\rho (u-w_{\ell })u\right)+\partial_{x}p=\partial_{x}\Pi_{\ell }+\left(\rho -\ell \tau \partial_{x}\left(\rho u\right)\right)f, \end{array}$$

(62) $$\begin{array}{c} \partial_{t}E+\partial_{x}\left(0.5\rho u^{2}\left(u-w_{\ell }\right)+\langle \rho_{\alpha }c_{p\alpha }\theta \left(u-w_{\ell \alpha }\right)\rangle \right)=\partial_{x}\left(-q_{\ell }+\Pi_{\ell }u\right)+\rho \left(u-w_{\ell }\right)f+Q. \end{array}$$

However, for further discretization, we prefer to use the original form of these PDEs since this approach allows us to derive a counterpart of Theorem 1.

Let us first introduce the mesh notation. Define the uniform mesh ${\bar{\omega }}_{h}$ on [−X,X], with the nodes $x_{i}=-X+ih$, 0⩽i⩽N, and the step $h=\frac{2X}{N}$. Let $\omega_{h}={\bar{\omega }}_{h}\setminus \left\{-X,X\right\}$ be its internal part. Define also an auxuliary mesh $\omega_{h}^{*}$ with the nodes $x_{i+1/2}=\left(i+1/2\right)h$, 0⩽i⩽N−1.

Let H(ω) be the space of functions defined on a mesh ω. We introduce the shifts of the argument $v_{-,i+1/2}=v_{i}$ and $v_{+,i+1/2}=v_{i+1}$ and the averages and difference quotients

$$\begin{array}{c} \;{\left[v\right]}_{i+1/2}=0.5\left(v_{i}+v_{i+1}\right),\;\delta v_{i+1/2}=\frac{v_{i+1}-v_{i}}{h},\;{\left[y\right]}_{i}^{*}=0.5\left(y_{i-1/2}+y_{i+1/2}\right),\;\delta^{*}y_{i}=\frac{y_{i+1/2}-y_{i-1/2}}{h} \end{array}$$

on functions $v\in H({\bar{\omega }}_{h})$ and $y\in H(\omega_{h}^{*})$, where $v_{i}=v\left(x_{i}\right)$ and $y_{i+1/2}=y\left(x_{i+1/2}\right)$.

First, for simplicity, let there be no body force (i.e., f=0). Following [41,47], we apply a non-standard spatial discretization of balance PDEs for the mass of the components, the momentum and total energy of the gas mixture (53)–(55) and construct their following three-point and symmetric semi-discrete counterparts

(63) $$\begin{array}{c} \partial_{t}\rho_{\alpha }+\delta^{*}\left({\left[\rho_{\alpha }\right]}_{\ln}\left(\left[u\right]-w_{\ell \alpha }\right)+d_{\alpha }\right)=0,\;\;\alpha =\bar{1,K}, \end{array}$$

(64) $$\begin{array}{c} \partial_{t}\left(\rho u\right)+\delta^{*}\left(\langle {\left[\rho_{\alpha }\right]}_{\ln}\left(\left[u\right]-w_{\ell \alpha }\right)\rangle \left[u\right]+\left[p\right]\right)=\delta^{*}\Pi_{\ell }, \end{array}$$

(65) $$\begin{array}{c} \partial_{t}E+\delta^{*}\left\{\langle \left({\left[E_{\alpha }\right]}_{2}+\left[p_{\alpha }\right]\right)\left(\left[u\right]-w_{\ell \alpha }\right)\rangle -0.25h^{2}\delta u\cdot \delta p\right\}=\delta^{*}\left(-q_{\ell }+\Pi_{\ell }\left[u\right]\right)+{\left[Q\right]}^{*} \end{array}$$

on $\omega_{h}\times \left[0,T\right]$. The main sought functions $\rho_{1}>0,\ldots ,\rho_{K}>0$, *u* and θ>0 together with the functions $p_{\alpha }$, $\varepsilon_{\alpha }$ and $E_{\alpha }$ are defined in space on the main mesh ${\bar{\omega }}_{h}$. In the equations, the above expressions (4) for $p_{\alpha }$, $\varepsilon_{\alpha }$ and $E_{\alpha }$ as well as (5) for ρ, *p*, ε and *E*, with ${\left|u\right|}^{2}=u^{2}$ and the coefficients *R* and $c_{V}$ from Formula (6), are exploited.

We apply the following discretizations of the regularizing velocities (56):

(66) $$\begin{array}{c} w_{\ell \alpha }=\ell \frac{\tau }{\left[\rho_{\alpha }\right]}\;\left[u\right]\delta \left(\rho_{\alpha }u\right)+\hat{w},\;\;\alpha =\bar{1,K},\;\;\hat{w}=\frac{\tau }{\left[\rho \right]}\;\left(\left[\rho \right]\left[u\right]\delta u+\delta p\right), \end{array}$$

as well as of the viscous stress, and heat flux and diffusion fluxes (57)–(60):

(67) $$\begin{array}{c} \Pi_{\ell }=\nu \delta u+\Pi_{\ell }^{\tau },\;\;\Pi_{\ell }^{\tau }=\left[u\right]\left[\rho \right]\hat{w}+\ell \tau \left\{\left[u\right]\delta p+\langle \gamma_{\alpha }{\left[p_{\alpha }\right]}_{1}\rangle \delta u-\langle \gamma_{\alpha }Q_{\alpha }\rangle +Q\right\}, \end{array}$$

(68) $$\begin{array}{c} q_{\ell }=-\varkappa \delta \theta +q^{d}+\ell q^{\tau },\;\; \end{array}$$

(69) $$\begin{array}{c} -q^{\tau }=\tau \left\{\left(\langle c_{V\alpha }\left[\rho_{\alpha }\right]\rangle \delta \theta -\left[\theta \right]\delta \langle R_{\alpha }\rho_{\alpha }\rangle \right){\left[u\right]}^{2}-Q\left[u\right]\right\}=\tau \left\{\left(\left[c_{V}\rho \right]\delta \theta -\left[\theta \right]\delta \left(R\rho \right)\right){\left[u\right]}^{2}-Q\left[u\right]\right\}, \end{array}$$

(70) $$\begin{array}{c} -d_{\alpha }={\langle d_{\alpha \beta }\left(\delta \left(G_{\alpha }-G_{\beta }\right)+\left(e_{\alpha }-e_{\beta }\right)\delta \theta \right)\rangle }_{\beta },\;\;q^{d}=\langle \left(\left[G_{\alpha }\right]+e_{\alpha }\left[\theta \right]\right)d_{\alpha }\rangle . \end{array}$$

The functions $w_{\ell \alpha }$, $\hat{w}$, $\Pi_{\ell \alpha }$, $q_{\ell \alpha }$, τ, $\nu_{\alpha }$, $\varkappa_{\alpha }$ and $Q_{\alpha }$ are defined in space on the auxiliary mesh $\omega_{h}^{*}$. Moreover, $G_{\alpha }$, the Gibbs potential of the component α, see Formula (15), is defined in space on ${\bar{\omega }}_{h}$, whereas the functions $d_{\alpha },e_{\alpha }$ and $q^{d}$ are defined in space on $\omega_{h}^{*}$.

Here, we apply nonstandard averages of $\rho_{\alpha }$, $p_{\alpha }$, $E_{\alpha }$ and $\varepsilon_{\alpha }$ of the form [41,47]

$$\begin{array}{c} {\left[\rho_{\alpha }\right]}_{\ln}=\frac{1}{\ln(\rho_{\alpha -};\rho_{\alpha +})},\;\;{\left[p_{\alpha }\right]}_{1}=R_{\alpha }\left[\rho_{\alpha }\right]\left[\theta_{\alpha }\right],\;\;{\left[E_{\alpha }\right]}_{2}=0.5{\left[\rho_{\alpha }\right]}_{\ln}u_{-}u_{+}+{\left[\rho_{\alpha }\right]}_{\ln}{\left[\varepsilon_{\alpha }\right]}^{\ln},\;\;\\ {}[\varepsilon_{\alpha }{]}^{\ln}=c_{V\alpha }{\left[\theta \right]}^{\ln},\;\;{\left[\theta \right]}^{\ln}:=\ln\left({\frac{1}{\theta }}_{-};{\frac{1}{\theta }}_{+}\right)=\theta_{-}\theta_{+}\ln\left(\theta_{-};\theta_{+}\right) \end{array}$$

that exploit the divided difference for the logarithmic function

$$\ln\left(a;b\right)=\frac{\ln b-\ln a}{b-a}\;\;\mathrm{for}\;\;a\neq b,\;\;\ln\left(a;a\right)=\frac{1}{a},\;\;a>0,\;\;b>0.$$

Consequently, we have $\langle \gamma_{\alpha }{\left[p_{\alpha }\right]}_{1}\rangle =\langle \left[\rho_{\alpha }\right]R_{\alpha }\gamma_{\alpha }\rangle \left[\theta \right]$ in expression (67). Note that $u_{-}u_{+}$ is similar to the geometric mean for $u^{2}$ (although it is negative for $u_{-}$ and $u_{+}$ of different signs). Concerning the case of $\tau =T\left(\rho ,u,\theta \right)$, one can set, in particular, $\tau =T\left([\rho ],[u],[\theta ]\right)$ or $\tau =\left[T(\rho ,u,\theta )\right]$ in space on $\omega_{h}^{*}$. In computations in Section 5 below, we apply the second formula.

This spatial discretization is close to a similar one recently constructed in ([41], Section 5) and differs from it by expression (66) for $w_{\ell \alpha }$ (approximating formulas (9), not (7), in the 1D case) and the much more general expression (70) for $d_{\alpha }$ in the case K⩾3. In its turn, this discretization in [41] generalises the original one from [47] in the case of the single-component gas dynamics to the considered multicomponent gas mixture dynamics PDEs.

Notice that the arising semi-discrete counterparts of Formula (17) are nontrivial: since

$$\begin{array}{c} \delta G_{\alpha }=-\left[\theta \right]\delta s_{\alpha }+\left(c_{p\alpha }-\left[s_{\alpha }\right]\right)\delta \theta ,\;\;\delta s_{\alpha }=-R_{\alpha }\ln\left(\rho_{\alpha -};\rho_{\alpha +}\right)\delta \rho_{\alpha }+c_{V\alpha }\ln\left(\theta_{-};\theta_{+}\right)\delta \theta , \end{array}$$

we obtain $\left[G_{\alpha }\right]+e_{\alpha }\left[\theta \right]=\left(c_{p\alpha }+e_{\alpha }\right)\left[\theta \right]-\left[s_{\alpha }\theta \right]$ and

$$\begin{array}{c} \delta G_{\alpha }+e_{\alpha }\delta \theta =R_{\alpha }\frac{\left[\theta \right]}{{\left[\rho_{\alpha }\right]}_{\ln}}\delta \rho_{\alpha }+\left(c_{p\alpha }-c_{V\alpha }\frac{\left[\theta \right]}{{\left[\theta \right]}_{\ln}}-\left[s_{\alpha }\right]+e_{\alpha }\right)\delta \theta . \end{array}$$

The main result in this section is a 1D semi-discrete counterpart of the balance equation for the mixture entropy, see Theorem 1. It corresponds to ([41], Theorem 2) but concerns another definition (66) of the semi-discrete regularizing velocity and deals with a much more general form of $d_{\alpha }$, for K⩾3; this form is applicable in [41] as well.

 **Theorem 3.** *Let $d_{\alpha \beta }=d_{\beta \alpha }⩾0$ for any α≠β. For the 1D semi-discrete method (63)–(70), the balance equation for the mixture entropy holds*

(71) $$\begin{array}{c} \partial_{t}\left(\rho s\right)+\delta^{*}\langle j_{\ell \alpha }\left[s_{\alpha }\right]\rangle =\delta^{*}\left(\left(\varkappa \delta \theta -\ell q^{\tau }\right)\left[\frac{1}{\theta }\right]-\langle e_{\alpha }d_{\alpha }\rangle \frac{{\left[\theta \right]}^{2}}{\theta_{-}\theta_{+}}+B_{h}^{\left(d\right)}\right)+{\left[P_{h}^{NS}+P_{h}^{\tau }\right]}^{*} \end{array}$$

*on $\omega_{h}\times \left[0,T\right]$, with the component mass fluxes $j_{\ell \alpha }={\left[\rho_{\alpha }\right]}_{\ln}\left(\left[u\right]-w_{\ell \alpha }\right)$ and the terms*

$$\begin{array}{c} B_{h}^{\left(d\right)}:=\langle R_{\alpha }j_{\ell \alpha }\left(1-\frac{\left[\rho_{\alpha }\right]}{{\left[\rho_{\alpha }\right]}_{\ln}}\right)+c_{V\alpha }j_{\ell \alpha }\left(1-{\left[\varepsilon_{\alpha }\right]}^{\ln}\left[\frac{1}{\varepsilon_{\alpha }}\right]\right)\rangle \\ -0.25h^{2}\left(\Pi_{\ell }\delta u-\langle d_{\alpha }\delta G_{\alpha }\rangle +\langle w_{\ell \alpha }\delta p_{\alpha }\rangle +Q\right)\delta \frac{1}{\theta },\\ P_{h}^{NS}:=\frac{1}{\theta_{-}\theta_{+}}\left\{\varkappa {\left(\delta \theta \right)}^{2}+\nu \left[\theta \right]{\left(\delta u\right)}^{2}+0.5\left[\theta \right]{\langle d_{\alpha \beta }{\left(\delta G_{\alpha }+e_{\alpha }\delta \theta -\left(\delta G_{\beta }+e_{\beta }\delta \theta \right)\right)}^{2}\rangle }_{\alpha ,\beta }\right\}⩾0,\\ P_{h}^{\tau }:=\frac{1}{\theta_{-}\theta_{+}}\{\left[\theta \right]\left[\rho \right]\frac{1}{\tau }{\hat{w}}^{2}+\ell \tau {\left[\theta \right]}^{2}\langle \frac{R_{\alpha }}{\left[\rho_{\alpha }\right]}{\left(\delta (\rho_{\alpha }u)\right)}^{2}\rangle \\ +\ell \tau \langle c_{V\alpha }\left[\rho_{\alpha }\right]{\left(\left[u\right]\delta \theta +\left(\gamma_{\alpha }-1\right)\left[\theta \right]\delta u-\frac{Q_{\alpha }}{2c_{V\alpha }\left[\rho_{\alpha }\right]}\right)}^{2}\rangle +\left[\theta \right]\langle Q_{\alpha }\left(1-\ell \frac{\tau \left(\gamma_{\alpha }-1\right)Q_{\alpha }}{4{\left[p_{\alpha }\right]}_{1}}\right)\rangle \}. \end{array}$$

The term ${\left[P_{h}^{NS}+P_{h}^{\tau }\right]}^{*}$ in Equation (71) is the semi-discrete entropy production. The first three terms of $P_{h}^{\tau }$ are non-negative, and the last term is non-negative for ℓ=0, as well as for ℓ=1 under the condition $\tau \langle \frac{\left(\gamma_{\alpha }-1\right)Q_{\alpha }^{2}}{4{\left[p_{\alpha }\right]}_{1}}\rangle ⩽Q.$ This condition is certainly true provided that $\tau \left(\gamma_{\alpha }-1\right)Q_{\alpha }⩽4{\left[p_{\alpha }\right]}_{1}$, $\alpha =\bar{1,K}$.

 **Proof.**  The following semi-discrete balance equations for the total mass and kinetic and internal energies of the mixture hold

(72) $$\begin{array}{c} \partial_{t}\rho +\delta^{*}j_{\ell }=0,\;\;0.5\partial_{t}\left(\rho u^{2}\right)+0.5\delta^{*}\left(j_{\ell }u_{-}u_{+}\right)+\left(\delta^{*}\left[p\right]\right)u=\left(\delta^{*}\Pi_{\ell }\right)u,\\ \partial_{t}\left(\rho \varepsilon \right)+\delta^{*}\langle j_{\ell \alpha }{\left[\varepsilon_{\alpha }\right]}^{\ln}\rangle =-\delta^{*}q_{\ell }-\langle p_{\alpha }\delta^{*}\left(\left[u\right]-w_{\ell \alpha }\right)\rangle +{\left[\Pi_{\ell }\delta u+\langle w_{\ell \alpha }\delta p_{\alpha }\rangle +Q\right]}^{*} \end{array}$$

on $\omega_{h}\times \left[0,T\right]$, with $j_{\ell }:=\langle j_{\ell \alpha }\rangle$. They are counterparts of the balance PDEs (29)–(31) and have recently been proved in ([41], Lemma 3), and their derivations remain valid for any $w_{\ell \alpha }$, $\Pi_{\ell }$ and $q_{\ell }$, in particular, given by expressions (66)–(68).

According to the proof of ([41], Theorem 2), the following preliminary 1D semi-discrete balance equation for the mixture entropy holds

(73) $$\begin{array}{c} \partial_{t}\left(\rho s\right)+\delta^{*}\langle j_{\ell \alpha }\left[s_{\alpha }\right]\rangle =\delta^{*}\left(\left(\varkappa \delta \theta -\ell q^{\tau }\right)\left[\frac{1}{\theta }\right]-\langle e_{\alpha }d_{\alpha }\rangle \frac{{\left[\theta \right]}^{2}}{\theta_{-}\theta_{+}}+B_{h}^{\left(d\right)}\right)\\ +{\left[\frac{\varkappa {\left(\delta \theta \right)}^{2}}{\theta_{-}\theta_{+}}+\frac{\nu \left[\theta \right]{\left(\delta u\right)}^{2}}{\theta_{-}\theta_{+}}+q^{d}\delta \frac{1}{\theta }-\langle d_{\alpha }\delta \frac{G_{\alpha }}{\theta }\rangle +\frac{\langle A_{\ell \alpha }\rangle }{\theta_{-}\theta_{+}}\right]}^{*}, \end{array}$$

where we have taken into account that $e_{\alpha }$ plays the role of $K^{-1}b_{\alpha }$ in the definition of $q^{d}$ in [41]. Recall that its derivation starts from the semi-discrete balance equations for the mass of components (63) and the internal energy of the mixture (72), and the specific form of $w_{\ell \alpha }$ does not matter in this derivation. Here, the following term and its decomposition

$$\begin{array}{c} A_{\ell }=-\ell q^{\tau }\delta \theta +\left(\Pi_{\ell }^{\tau }\delta u+\langle w_{\ell \alpha }\delta p_{\alpha }\rangle +Q\right)\left[\theta \right]=\langle A_{\ell \alpha }\rangle \end{array}$$

are involved, with

$$\begin{array}{c} A_{\ell \alpha }=-\ell q_{\alpha }^{\tau }\delta \theta +\left(\Pi_{\ell \alpha }^{\tau }\delta u+w_{\ell \alpha }\delta p_{\alpha }+Q_{\alpha }\right)\left[\theta \right],\;\;-q_{\alpha }^{\tau }=\tau \left\{{\left[u\right]}^{2}\left(c_{V\alpha }\left[\rho_{\alpha }\right]\delta \theta -R_{\alpha }\left[\theta \right]\delta \rho_{\alpha }\right)-Q_{\alpha }\left[u\right]\right\},\\ \Pi_{\ell \alpha }^{\tau }=\left[u\right]\left[\rho_{\alpha }\right]\hat{w}+\ell \tau \left\{\left[u\right]\delta p_{\alpha }+\gamma_{\alpha }{\left[p_{\alpha }\right]}_{1}\delta u-\left(\gamma_{\alpha }-1\right)Q_{\alpha }\right\}, \end{array}$$

where only the term $\left[u\right]\left[\rho_{\alpha }\right]\hat{w}$ is written differently, but the corresponding average $\langle \left[u\right]\left[\rho_{\alpha }\right]\hat{w}\rangle =\left[u\right]\left[\rho \right]\hat{w}$ is the same term of $\Pi_{\ell }^{\tau }$ in the above expression for $A_{\ell }$.

Let us transform the difference of the third and fourth terms under the sign ${\left[\cdot \right]}^{*}$ on the right in Equation (73). We need the elementary formulas

$$\begin{array}{c} \delta \frac{G_{\alpha }}{\theta }=\left[G_{\alpha }\right]\delta \frac{1}{\theta }+\left(\delta G_{\alpha }\right)\left[\frac{1}{\theta }\right],\;\;\delta \frac{1}{\theta }=-\frac{\delta \theta }{\theta_{-}\theta_{+}},\;\;\left[\frac{1}{\theta }\right]=\frac{\left[\theta \right]}{\theta_{-}\theta_{+}}. \end{array}$$

Applying them and then identity (16), we obtain

$$\begin{array}{c} q^{d}\delta \frac{1}{\theta }-\langle d_{\alpha }\delta \frac{G_{\alpha }}{\theta }\rangle =\left(q^{d}-\langle d_{\alpha }\left[G_{\alpha }\right]\rangle \right)\delta \frac{1}{\theta }-\langle d_{\alpha }\delta G_{\alpha }\rangle \left[\frac{1}{\theta }\right]\\ =-\frac{\left[\theta \right]}{\theta_{-}\theta_{+}}\langle d_{\alpha }\left(e_{\alpha }\delta \theta +\delta G_{\alpha }\right)\rangle =\frac{\left[\theta \right]}{2\theta_{-}\theta_{+}}{\langle d_{\alpha \beta }{\left(\delta G_{\alpha }+e_{\alpha }\delta \theta -\left(\delta G_{\beta }+e_{\beta }\delta \theta \right)\right)}^{2}\rangle }_{\alpha ,\beta }. \end{array}$$

Next, using expressions (66), we can extract from $A_{\ell \alpha }$ the term $\left[\theta \right]A_{\ell \alpha }^{\prime }$ such that

$$\begin{array}{c} A_{\ell \alpha }^{\prime }:=\left[\rho_{\alpha }\right]\left[u\right]\hat{w}\delta u+w_{\ell \alpha }\delta p_{\alpha }=\left(\left[\rho_{\alpha }\right]\left[u\right]\delta u+\delta p_{\alpha }\right)\hat{w}+\ell \frac{\tau }{\left[\rho_{\alpha }\right]}\;\left[u\right]\left(\delta (\rho_{\alpha }u)\right)\delta p_{\alpha }, \end{array}$$

and, according to ([41], Appendix A) or [47], the following formula holds

$$\begin{array}{c} A_{\ell \alpha }-\left[\theta \right]\left(\left[\rho_{\alpha }\right]\left[u\right]\delta u+\delta p_{\alpha }\right)\hat{w}=\ell \tau {\left[\theta \right]}^{2}\frac{R_{\alpha }}{\left[\rho_{\alpha }\right]}{\left(\delta (\rho_{\alpha }u)\right)}^{2}\\ +\ell \tau c_{V\alpha }\left[\rho_{\alpha }\right]{\left(\left[u\right]\delta \theta +\left(\gamma_{\alpha }-1\right)\left[\theta \right]\delta u-\frac{Q_{\alpha }}{2c_{V\alpha }\left[\rho_{\alpha }\right]}\right)}^{2}+\left[\theta \right]Q_{\alpha }\left(1-\ell \frac{\tau \left(\gamma_{\alpha }-1\right)Q_{\alpha }}{4{\left[p_{\alpha }\right]}_{1}}\right). \end{array}$$

Applying the operation 〈·〉 to it and accomplishing the transformations

(74) $$\begin{array}{c} \langle \left[\theta \right]\left(\left[\rho_{\alpha }\right]\left[u\right]\delta u+\delta p_{\alpha }\right)\hat{w}\rangle =\left[\theta \right]\left(\left[\rho \right]\left[u\right]\delta u+\delta p\right)\hat{w}=\left[\theta \right]\left[\rho \right]\frac{1}{\tau }{\hat{w}}^{2}, \end{array}$$

we complete the proof. □

As in the differential case, the entropy production remains non-negative for τ⩾0, where one should pass to another form for the first relaxation term in $P_{h}^{\tau }$ inside the curly brackets: $\left[\theta \right]\left[\rho \right]\frac{1}{\tau }{\hat{w}}^{2}=\tau \frac{\left[\theta \right]}{\left[\rho \right]}{\left(\left[\rho \right]\left[u\right]\delta u+\delta p\right)}^{2}.$

At the end of the section, following [41,47], we generalize the constructed semi-discrete method and Theorem 3 to the case of any *f*. Recall the general momentum and total energy balance PDEs (54) and (55) and expressions for the regularized velocities (56), and generalize the semi-discrete Equations (64) and (65) by adding, respectively, the terms

$${\left[\rho_{*\ell }f\right]}^{*},\;\;\langle {\left[\left[\rho_{\alpha }\right]\left(\left[u\right]-w_{\ell \alpha }\right)f\right]}^{*}\rangle +0.25h^{2}{\left[\rho_{*\ell }\left(\delta u\right)\left(\delta \frac{1}{\theta }\right)f\right]}^{*}\theta$$

to their right-hand sides, with the functions $\rho_{*\ell }:=\left[\rho \right]-\ell \tau \delta \left(\rho u\right)$ and *f* defined in space on $\omega_{h}^{*}$. We also generalize the expression for $\hat{w}$ as $\hat{w}=\frac{\tau }{\left[\rho \right]}\left(\left[\rho \right]\left[u\right]\delta u+\delta p-\left[\rho \right]f\right)$.

The new terms with *f* produce the following additional term on the right-hand side of the semi-discrete balance equation for the internal energy (72):

$$\Psi :=\langle {\left[\left[\rho_{\alpha }\right]\left(\left[u\right]-w_{\ell \alpha }\right)f\right]}^{*}\rangle -{\left[\rho_{*\ell }f\right]}^{*}u+0.25h^{2}{\left[\rho_{*\ell }\left(\delta u\right)\left(\delta \frac{1}{\theta }\right)f\right]}^{*}\theta .$$

To derive the semi-discrete balance equation for the mixture entropy (71), one should multiply this term by $\frac{1}{\theta }$ and transform the result. The required transformation was accomplished in [41] for general $w_{\ell \alpha }=\ell \frac{\tau }{\left[\rho_{\alpha }\right]}\;\left[u\right]\delta \left(\rho_{\alpha }u\right)+{\hat{w}}_{\alpha }$ with any ${\hat{w}}_{\alpha }$, and in our case where ${\hat{w}}_{\alpha }=\hat{w}$ is independent of α, it leads to the formulas

$$\begin{array}{c} \frac{\Psi }{\theta }=-{\left[\langle \left[\rho_{\alpha }\right]\rangle \hat{w}f\left[\frac{1}{\theta }\right]\right]}^{*}+\delta^{*}C_{h},\;\;C_{h}:=0.25h^{2}\left(\left[\rho \right]\hat{w}\delta \frac{1}{\theta }+\rho_{*\ell }\left(\delta u\right)\left[\frac{1}{\theta }\right]\right)f. \end{array}$$

As a result, in the preliminary entropy balance equation (73), the additional term $-\left[\theta \right]\left[\rho_{\alpha }\right]\hat{w}f$ appears in $A_{\ell \alpha }$, and the term $C_{h}$ should be added to $B_{h}^{\left(d\right)}$. Thus, Formula (74) takes the form

$$\langle \left[\theta \right]\left(\left[\rho_{\alpha }\right]\left[u\right]\delta u+\delta p_{\alpha }-\left[\rho_{\alpha }\right]f\right)\hat{w}\rangle =\left[\theta \right]\left(\left[\rho \right]\left[u\right]\delta u+\delta p-\left[\rho \right]f\right)\hat{w}=\left[\theta \right]\left[\rho \right]\frac{1}{\tau }{\hat{w}}^{2}.$$

With the given *f*-dependent extensions, Theorem 3 remains valid.

## 5. Numerical Experiments

We are still dealing with the 1D system of PDEs. Let us compare three cases *A*, *B* and *C* of the regularizing velocities $w_{\ell \alpha }$ in the balance PDEs (53)–(55):

$$\begin{array}{c} w_{\ell \alpha }=\ell \frac{\tau }{\rho_{\alpha }}u\partial_{x}\left(\rho_{\alpha }u\right)+\hat{w},\;\;\hat{w}=\tau \left(u\partial_{x}u+\frac{1}{\rho }\partial_{x}p\right)\;\;\left(A\right);\\ w_{\ell \alpha }=\ell \frac{\tau }{\rho_{\alpha }}u\partial_{x}\left(\rho_{\alpha }u\right)+{\hat{w}}_{\alpha },\;\;{\hat{w}}_{\alpha }=\tau \left(u\partial_{x}u+\frac{1}{\rho_{\alpha }}\partial_{x}p_{\alpha }\right)\;\;\left(B\right);\;\;w_{\ell \alpha }=\frac{\tau }{\rho }u\partial_{x}\left(\rho u\right)+\hat{w}\;\;\left(C\right), \end{array}$$

with ℓ=1 and α=1,2 (the case of binary mixtures), considered, respectively, in this paper (see Formula (56)), papers [34,35,41] (see also Formula (7) for n=1) and [34,37,39]. Case *A* is the main one below, and we demonstrate some its advantages over the other two cases. In case *C*, $w_{\ell \alpha }$ is independent of *ℓ* and α. We discretize these expressions, respectively, according to Formula (66) as well as

$$\begin{array}{c} w_{\ell \alpha }=\ell \frac{\tau }{\left[\rho_{\alpha }\right]}\;\left[u\right]\delta \left(\rho_{\alpha }u\right)+{\hat{w}}_{\alpha },\;\;{\hat{w}}_{\alpha }=\tau \left(\left[u\right]\delta u+\frac{1}{\left[\rho_{\alpha }\right]}\delta p_{\alpha }\right)\;\;\mathrm{and}\;\;w_{\ell \alpha }=\frac{\tau }{\left[\rho \right]}\;\left[u\right]\delta \left(\rho u\right)+\hat{w}. \end{array}$$

We consider four test examples known in the literature. In Examples 1–3, we take the following piecewise constant initial data $\left(\rho_{1},\rho_{2},p,u\right){|}_{t=0}=\left(\rho_{1}^{0},\rho_{2}^{0},p^{0},u^{0}\right)$ (a Riemann problem) and piecewise constant physical parameters

$$\begin{array}{c} \left(\rho_{1}^{0},\rho_{2}^{0},p^{0},u^{0},\gamma ,c_{V}\right)\left(x\right)=\left\{\begin{array}{cc} (\rho_{1l},\rho_{2l},p_{l},u_{l},\gamma_{1},c_{V1}), & -X⩽x<0\\ (\rho_{1r},\rho_{2r},p_{r},u_{r},\gamma_{2},c_{V2}), & 0⩽x⩽X \end{array}\right.. \end{array}$$

Moreover, $\rho_{1r}=\rho_{2l}=0$, although, in computations, we take them suitably small positive (equal $10^{-10}$) instead. The parameters of the gases to the left and right of x=0 and the final time of computations $t_{fin}$ can be found in Table 1. Note that there the simplest values $c_{V1}=c_{V2}=1$ in Examples 1, 3 and 4 were not required originally (since in the case τ=ν=ϰ=0, there exists a closed Euler-type system of PDEs for the sought functions ρ, γ, *u* and θ, see details in Appendix A), and we have chosen them ourselves. The initial temperature $\theta^{0}$ is defined in accordance with Formulas (4) and (5): $p^{0}=R\rho^{0}\theta^{0}=\left(\left(\gamma_{1}-1\right)c_{V1}\rho_{1}+\left(\gamma_{2}-1\right)c_{V2}\rho_{2}\right)\theta^{0}.$ The boundary values of the sought functions at x=±X in time are kept the same as their values given at t=0. We also take X=0.5 in Examples 1–3 and X=5 in Example 4.

In Examples 1–3, the initial pressure drop rapidly increases: $\frac{p_{l}}{p_{r}}=1,194.3,2500.$

Recall that in order to avoid loss of accuracy in computation of ${\left[\rho_{\alpha }\right]}_{\ln}$ and ${\left[\varepsilon_{\alpha }\right]}^{\ln}$, one can apply the trapezoidal or midpoint rule to the integral representation of ln(a;b) in the case $\frac{b}{a}\approx 1$:

$$\ln\left(a;b\right)=\int_{0}^{1}\frac{1}{(1-r)a+br}\;dr\approx \frac{1}{2a}+\frac{1}{2b},\;\frac{2}{a+b}.$$

We apply them for ε and $\rho_{\alpha }$, respectively, that leads to the formulas ${\left[\rho_{\alpha }\right]}_{\ln}\approx \left[\rho_{\alpha }\right]$ and ${\left[\varepsilon_{\alpha }\right]}^{\ln}\approx \left[\varepsilon_{\alpha }\right]$.

We introduce a non-uniform mesh in time $0=t_{0}<t_{1}<\ldots <t_{\bar{m}}=t_{fin}$, with the steps $h_{tm}=t_{m}-t_{m-1}$. We take the relaxation parameter and the artificial viscosity and heat conductivity coefficients according to Formula (19) and $\nu =\frac{4}{3}\mu +\lambda$:

$$\tau_{i}^{m}=\frac{ah}{c_{si}^{m}+i_{\tau }\left|u_{i}^{m}\right|},\;\;\nu =\tau \left(a_{S1}p_{1}+a_{S2}p_{2}\right),\;\;\varkappa =\tau a_{Pr}\left(\gamma_{1}c_{V1}p_{1}+\gamma_{2}c_{V2}p_{2}\right).$$

Here, 0<a<1 is a parameter, $h=\frac{2X}{N}$, $i_{\tau }=0$ in Examples 1 and 3 or $i_{\tau }=1$ in Examples 2 and 4, and, for example, $u_{i}^{m}=u\left(x_{i},t_{m}\right)$. In addition, $a_{S1}=a_{S2}=\frac{3}{4}$ and $a_{Pr}=1$ in Examples 1–3.

To complement the above spatial discretization, we apply the simplest explicit Euler method for the temporal discretization, together with the automatic choice of the time steps $h_{tm}=t_{m}-t_{m-1}$ according to

$$h_{tm}=\frac{\beta h}{\max_{i}(c_{si}^{m-1}+|u_{i}^{m-1}\left|\right)},\;\;1⩽m⩽\bar{m}-1,\;\;h_{t\bar{m}}=t_{fin}-t_{\bar{m}-1}⩽\frac{\beta h}{\max_{i}\left(c_{si}^{\bar{m}-1}+\left|u_{i}^{\bar{m}-1}\right|\right)},$$

where β is the Courant-type parameter. A linearized stability (more precisely, $L^{2}$-dissipativity) conditions for such an explicit scheme theoretically and practically were studied in [19,49] in the single-component gas case. In every example, we adjust the parameters *a* and β. If the values of $\rho_{1}$ or $\rho_{2}$ less than $10^{-10}$ arise at the upper time level, we replace them by $10^{-10}$.

Notice that, with a code for the considered discretization of the single-component gas dynamics PDEs, as in [49], at one’s disposal, it is not difficult to extend it to the case of the binary gas mixture. Proposition A2 in Appendix D (the 1D discrete counterpart of Proposition 2) was applied for initial testing of our code for mixtures.

 **Example 1.** (the test from ([54], p. 266))*. In this first rather simple test, there is a contact discontinuity between the two gases that moves to the right with constant velocity u; the pressure p and temperature θ are also constant. The Mach number of the mixture ranges approximately from 0.14 to 0.42 and is not high. However, it is known that not all numerical methods are able to reproduce this constancy well, especially for moderate N.*

**Figure 1:**
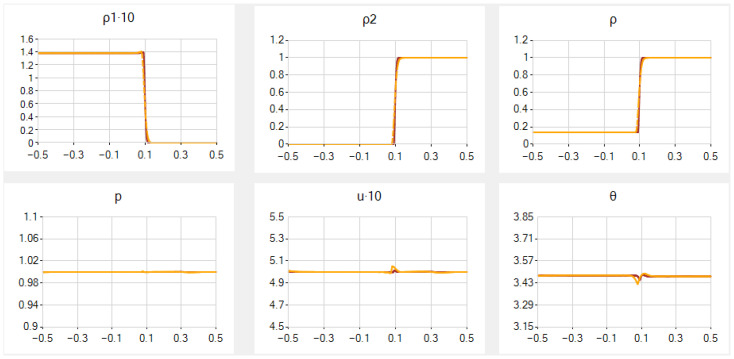
Example 1, case *A*. The results for a=0.5, β=0.7, N=251 (orange) and 1001 (brown) for t=0.2. Hereafter, the graphs for two values of *N* mainly almost coincide except for vicinities of the contact discontinuity and the shock wave.

In the main case *A*, the results for a=0.5, β=0.7, N=251 (similarly to [54]) and 1001 are given in Figure 1; note that the scales for *p*, *u* and θ are enlarged there. In this and other examples, we exhibit graphs of six functions: $\rho_{1}$, $\rho_{2}$, ρ, *p*, *u* and θ. The deviations from constant values near the contact discontinuity are very small in $\rho_{1}$ and *p*, slightly larger for *u* and θ. They diminish as *N* grows and, for N=1001, they disappear for *p*, almost disappear in $\rho_{1}$ and *u* and become very small in θ. Hereafter, the graphs for two values of *N* in general almost coincide except for vicinities of the contact discontinuity and the shock wave; the differences become more visible after several magnifications.

**Figure 2:**
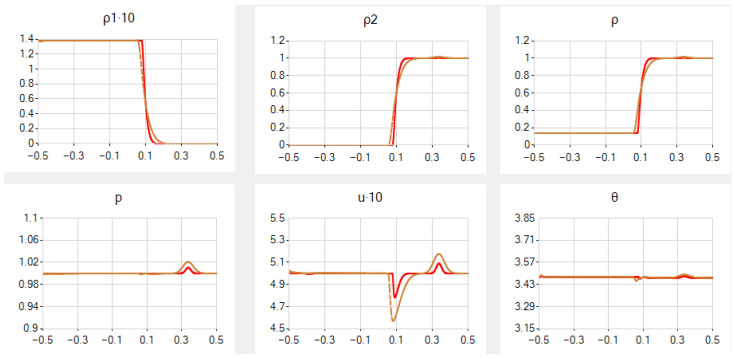
Example 1, case *B*. The results for a=0.2, β=0.1, N=251 (bronze) and 1001 (red) for t=0.2.

In case *B*, we need to take smaller *a* and especially β to obtain suitable results: a=0.2 and β=0.1. However, for N=251, the results are still not so nice: the behaviour of $\rho_{1}$, $\rho_{2}$ and ρ is too smooth near the contact discontinuity, the deviations from constant values near the contact discontinuity are very small for *p*, small for θ, but rather large in *u*. For N=1001, the results become better, see Figure 2.

In case *C*, the results for a=0.2, β=0.1 and N=251, are worse than in case *B*: all the deviations mentioned above in it are significantly larger. Hereafter, we mainly omit the graphs in this case for brevity (except for Example 3).

 **Example 2.** **(a version of the Sod problem)** from ([52], Table I)*. In the original paper, $t_{fin}$ was missed, and we adjusted its value to the graphs given there. The final solution contains the contact discontinuity between the two gases, with jumps in the values of $\rho_{1}$, $\rho_{2}$, ρ and θ, but not p and u, as well as a rarefaction wave in gas 1 to the left and a shock wave (the strong discontinuity) in gas 2 to the right of the contact discontinuity. The functions $\rho_{1}$ and p are non-increasing, whereas $\rho_{2}$, ρ, u and θ are non-monotone, with the maximal values of $\rho_{2}$, u and θ in front of the shock. In addition, $\rho_{2}$ is piecewise constant. The final maximal Mach number is $M_{max}:=\max_{i}\frac{|u_{i}^{\bar{m}}|}{c_{si}^{\bar{m}}}\approx 1.67$, so now the flow is partially supersonic (note that u=M=0 closely to the boundaries). Note that the parameters are not scaled in this example in contrast to the rest of them, and, at first, we do not use scaling in our computations to check further our method’s capabilities. We choose a=0.5 and β=0.4. Notice that, in this example, we can take zero artificial viscosity ν=0 without essential changing the results.*

**Figure 3:**
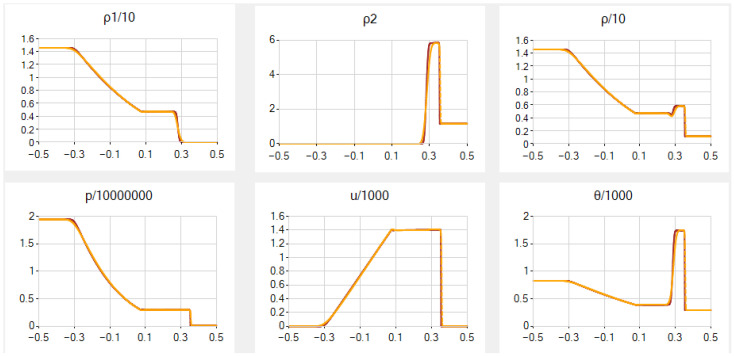
Example 2, case *A*. The results for a=0.5, β=0.4, N=501 (orange) and 2001 (brown) for t=0.0002.

In the main case *A*, the results for N=501 and 2001 are given in Figure 3, and they correspond well to those from [52] excluding the very small hollow in ρ at the contact discontinuity. Notice that scaling *u* and θ by the natural divisors $u_{*}=\theta_{*}=1000$ (with no scaling of $\rho_{1}$, $\rho_{2}$ and *x*) and consequently *p* by the divisor $p_{*}=10^{6}$, see details in Appendix B, does not improve the results.

**Figure 4:**
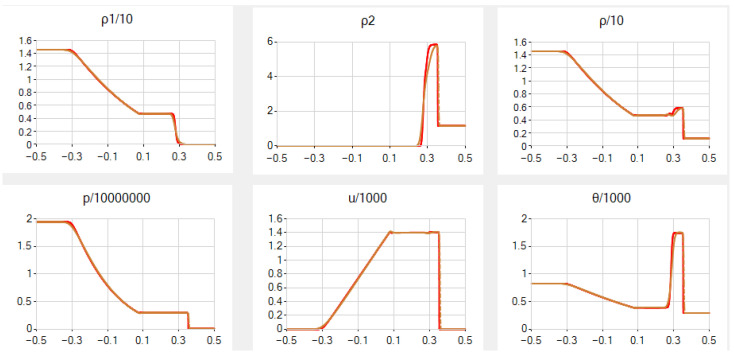
Example 2, case *B*. The results for a=0.5, β=0.4, N=501 (bronze) and 2001 (red) for t=0.0002.

In case *B*, once again, the results for the same *N* are rather nice, but the graph of $\rho_{2}$ is slightly more smoothed and the graph of ρ has an additional false step, both near the contact discontinuity. See Figure 4.

In case *C*, the results for the same *N* are of low quality in general. Despite the fact that $\rho_{1}$, *p* and θ are computed rather accurately, the graphs of $\rho_{2}$ and *u* have high “fingers” at the point of contact discontinuity. Additionally, there are single oscillations of relatively small amplitude in *p* and of high-amplitude in ρ near that point. Since the unknown functions satisfy the unified system of PDEs, it is somewhat surprising that some of them are computed accurately, while the rest are not. In this case, even in a simplified example, low quality of numerical results has recently been detected in [59] for another scheme.

We also briefly comment on two simpler Sod problems, see Examples 1 and 2 in [41], where the initial pressure drop is much less and equals 10 and 20 and the case *B* was studied only. If the initial pressure drop equals 10, results in cases *A* and *B* are very close and both equally correct. Even in case *C*, results are rather well, though with small ledges in the graphs of $\rho_{2}$ and *u* at the contact discontinuity.

If the initial pressure drop equals 20, the best results are in case *A*. In case *B*, they are also nice, though the graph of $\rho_{2}$ is slightly more smoothed and the graph of ρ has a slightly deeper hollow near the contact discontinuity. In case *C*, the results are already poor in general: though $\rho_{1}$, *p* and θ are computed rather accurately, the graphs of $\rho_{2}$ and *u* have visible “fingers” at the point of contact discontinuity, and the graph of ρ has a single oscillation near that point.

 **Example 3.** **(stiff two-gas shock-tube problem)** from ([53], Test 5.4). *In [53], the values $p_{l}$ and $p_{r}$ were confused, and $t_{fin}$ was not specified so we adjusted its value. In this example, the initial pressure drop equals 2500 and is very high. In general, the behavior of the final solution is similar to the previous example. However, the support of the maximal value of $\rho_{2}$ is narrower, θ is non-increasing and the jumps in the values of $\rho_{2}$, ρ and θ are high. Furthermore, $M_{max}\approx 1.44$, so the flow is partially supersonic once again. We take a=0.25 and β=0.4.*

**Figure 5:**
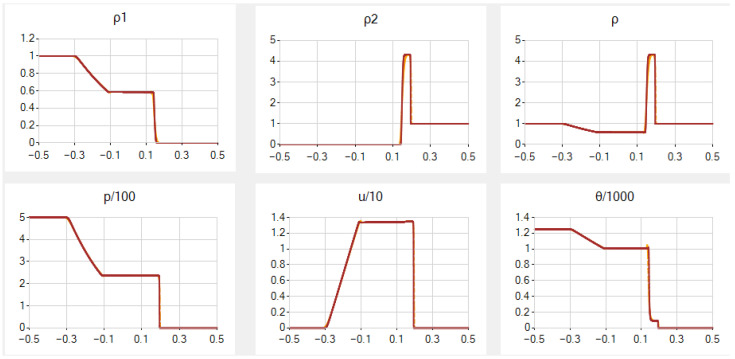
Example 3, case *A*. The results for a=0.25, β=0.4, N=1001 (orange) and 4001 (brown) for t=0.011.

In the main case *A*, for N=4001, the results correspond well to those in [53], see Figure 5. For smaller N=1001, the graphs of $\rho_{2}$ and ρ are more smoothed near the contact discontinuity, as well as *u* and θ have very small ledges near the right end of the rarefaction wave and the contact discontinuity, respectively, though the rest of the graphs look well.

In case *B*, the computation for the same β=0.4 and *N* fails. For four times smaller β=0.1, the results are not bad (see them for N=4001 in ([41], Example 3)), but now the graph of $\rho_{1}$ acquires an additional rather high, though very narrow, false step, and the graphs of $\rho_{2}$ and ρ are slightly more smoothed, both near the point of contact discontinuity.

**Figure 6:**
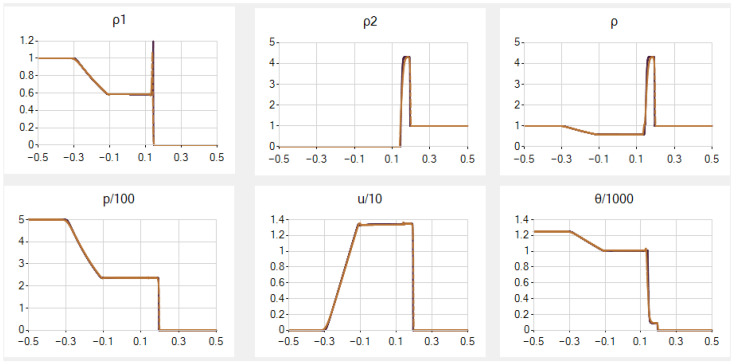
Example 3, case *C*. The results for a=0.25, β=0.2, N=1001 (copper) and 4001 (purple) for t=0.011. The graphs of $\rho_{1}$ have high “fingers” at the contact discontinuity.

In case *C*, the results are specific, see Figure 6. Now, for β=0.2 and the same *N*, the graph of $\rho_{1}$ has a large “finger” at the contact discontinuity which height is about twice the value of $\rho_{1}$ to the left of that point. Surprisingly, the rest of graphs look rather accurate including even ρ (although after a magnification, the defect in its graph just to the right of the contact discontinuity becomes more noticeable), and the situation does not change for some larger times as well. This figure shows that the rather accurate computation of ρ, *p*, *u* and θ for the mixture does not guarantee the same concerning both $\rho_{1}$ and $\rho_{2}$ for the components (the latter graphs are sometimes omitted from the numerical results).

 **Example 4.** **(Shock-bubble interaction problem)** from ([55], Test 3.4)*. In this example, the structure of the initial parameters is more complicated than in previous examples:*

(ρ1,ρ2,u,1.4p,γ)|t=0=(0,.37640.1819,1.22,001,.56981.648),|x+4|<12(1,.37640,.18191.22,001,.56981.4),x<−3(1.3764,0,.18190.8864,1.5698,1.4),otherwise,

*thus, in gas 1 with $\gamma_{1}=1.4$, there is “the bubble” of gas 2 with $\gamma_{2}=1.648$ moving to the right. Here, $t_{fin}=4$, and $M_{\min}:=\min_{i}\frac{|u_{i}^{\bar{m}}|}{c_{si}^{\bar{m}}}\approx 0.32$ and $M_{\max}\approx 1.22$, so the flow is transonic. In this problem, shock waves and contact discontinuities interact, that complicates computations. Let a=0.5 and β=0.2.*

**Figure 7:**
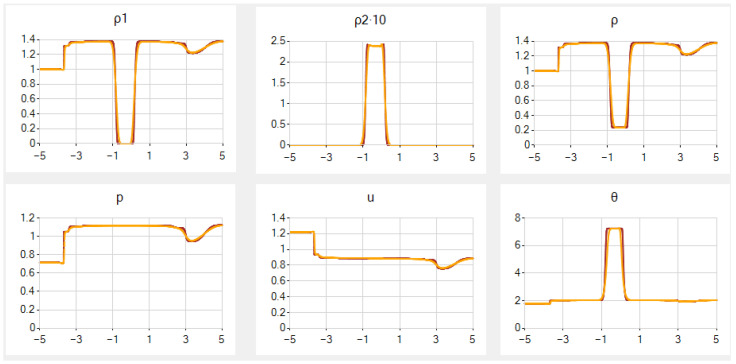
Example 4, case *A*. The results for a=0.5, β=0.2, N=1001 (orange) and 4001 (brown) for t=4.

In the main case *A*, the results for N=1001 and 4001 are given in Figure 7. For N=4001, the graphs of ρ and *p* are very close to those only presented in [55]. The very small ledges in the graphs of $\rho_{2}$ are observed at the two points of contact discontinuities. To achieve their smallness, we have changed the values $a_{S2}=0.15$ and $a_{Pr}=10$.

**Figure 8:**
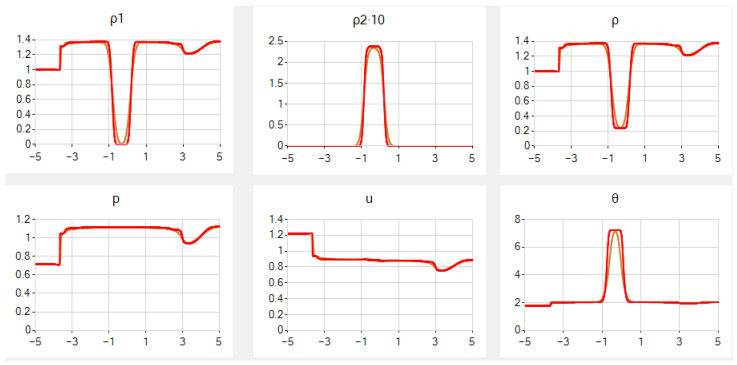
Example 4, case *B*. The results for a=0.5, β=0.2, N=1001 (bronze) and 4001 (red) for t=4.

In case *B*, the results for the same *N* are shown in Figure 8. On the one hand, the overall quality of the solution for N=1001 is high, without any ledges, even for the same standard $a_{S\alpha }$ and $a_{Pr}$ as in previous examples. On the other hand, $\rho_{1}$, $\rho_{2}$, ρ and θ are too smooth near the two mentioned points, especially for N=1001.

In case *C*, the graphs of $\rho_{2}$ have high “fingers” at the same two points which we could not remove by changing the parameters. Nevertheless, as in Example 3, the other graphs are correct except for a small “finger” in θ at the right of the same points.

Finally, we see that the results in the main case *A* are better or not worse than in the other two cases. The weakest results are in case *C*.

## Figures and Tables

**Table 1:** The initial parameters to the left and right of the discontinuity between two gases and the final time of computations.

Example	ρ	*p*	*u*	γ	$c_{V}$	$t_{\mathrm{fin}}$
(1) left	0.138	1	0.5	1.67	3.11	0.2
(1) right	1	1	0.5	1.4	0.72	
(2) left	14.54903	1.943$\;\times \;10^{7}$	0	5/3	2420	0.0002
(2) right	1.16355	$10^{5}$	0	1.4	732	
(3) left	1	500	0	1.4	1	0.011
(3) right	1	0.2	0	1.6	1	

## Data Availability

The datasets generated during the current study are available from the corresponding author on reasonable request.

## Appendix A

In the case τ=μ=λ=ϰ=0 and $d_{1}=\ldots =d_{K}=0$, the regularized balance PDEs for the mass of components, total momentum and total energy of the mixture (1)–(3) are reduced to the following Euler-type system of PDEs

(A1) $$\begin{array}{c} \partial_{t}\rho_{\alpha }+\mathrm{div}\left(\rho_{\alpha }u\right)=0,\;\;\alpha =\bar{1,K}, \end{array}$$

(A2) $$\begin{array}{c} \partial_{t}\left(\rho u\right)+\mathrm{div}\left(\rho u⊗u\right)+\nabla p=\rho f, \end{array}$$

(A3) $$\begin{array}{c} \partial_{t}E+\mathrm{div}\left((E+p)u\right)=\rho u\cdot f+Q. \end{array}$$

We consider differentiable solutions to these PDEs and first obtain some of their corollaries. The balance PDEs for the mass, kinetic and internal energies of the mixture (29)–(31) are essentially simplified as well:

(A4) $$\begin{array}{c} \partial_{t}\rho +\mathrm{div}\left(\rho u\right)=0, \end{array}$$

(A5) $$\begin{array}{c} \;0.5\partial_{t}{\left(\rho |u\right|}^{2})+0.5\mathrm{div}\left({\rho |u|}^{2}u\right)+\left(\nabla p\right)\cdot u=\rho f\cdot u,\;\;\partial_{t}\left(\rho \varepsilon \right)+\mathrm{div}\left(\rho \varepsilon u\right)+p\mathrm{div}u=Q. \end{array}$$

Differentiating on the left in the Euler total momentum balance equation (A2), using the total mass balance equation (A4) and dividing the result by ρ, we derive the equation for u:

(A6) $$\begin{array}{c} \partial_{t}u+\left(u\cdot \nabla \right)u+\frac{1}{\rho }\nabla p=f. \end{array}$$

Differentiating the ratio $C_{\alpha }=\frac{\rho_{\alpha }}{\rho }$ and using the Euler mass balance PDEs (A1) and (A4), we obtain

$$\begin{array}{c} \partial_{t}C_{\alpha }+u\cdot \nabla C_{\alpha }=\frac{1}{\rho^{2}}\left\{\left(\partial_{t}\rho_{\alpha }+u\cdot \nabla \rho_{\alpha }\right)\rho -\rho_{\alpha }\left(\partial_{t}\rho +u\cdot \nabla \rho \right)\right\}\\ =\frac{1}{\rho^{2}}\left\{\left(\partial_{t}\rho_{\alpha }+\mathrm{div}\left(\rho_{\alpha }u\right)\right)\rho -\rho_{\alpha }\left(\partial_{t}\rho +\mathrm{div}\left(\rho u\right)\right)\right\}=0. \end{array}$$

By definition (6) of *R* and $c_{V}$ and the formula $\gamma =\frac{R}{c_{V}}+1$ we immediately find

$$\begin{array}{c} \partial_{t}R+u\cdot \nabla R=0,\;\;\partial_{t}c_{V}+u\cdot \nabla c_{V}=0,\\ \partial_{t}\gamma +u\cdot \nabla \gamma =\frac{1}{c_{V}^{2}}\left[\left(\partial_{t}R+u\cdot \nabla R\right)c_{V}-R\left(\partial_{t}c_{V}+u\cdot \nabla c_{V}\right)\right]=0. \end{array}$$

Note that due to Equation (A4), we can also write the derived PDEs in the divergent form

$$\partial_{t}\left(\rho \varphi \right)+\mathrm{div}\left(\rho \varphi u\right)=0\;\;\mathrm{for}\;\;\varphi =C_{\alpha },R,c_{V}.$$

The derived equation for γ and the balance equation for the internal energy (A5) imply

$$\begin{array}{c} \partial_{t}p+u\cdot \nabla p=\left(\partial_{t}\gamma +u\cdot \nabla \gamma \right)\rho \varepsilon +\left(\gamma -1\right)\left(\partial_{t}\left(\rho \varepsilon \right)+u\cdot \nabla \left(\rho \varepsilon \right)\right)\\ =(\gamma -1)(-\rho \varepsilon \mathrm{div}u-p\mathrm{div}u+Q) \end{array}$$

since p=(γ−1)ρε and, consequently, the following equation for *p*:

(A7) $$\begin{array}{c} \partial_{t}p+u\cdot \nabla p+\gamma p\mathrm{div}u=\left(\gamma -1\right)Q. \end{array}$$

Equations (A6) and (A7) are well-known in the case of the single-component gas dynamics PDEs. In the general case, they are known too, and we have included their short derivations for completeness and the reader’s convenience.

The derived PDEs have an important corollary. The original system of PDEs (A1)–(A3) consists of K+n+1 PDEs for K+n+1 sought scalar functions ρ, u and θ. Now we see that the closed systems of PDEs exist for n+4 sought scalar functions ρ, *R*, $c_{V}$, u and θ, or even n+3 sought scalar functions ρ, γ, u and ε, since p=Rρθ=(γ−1)ρε and $E={0.5\rho |u|}^{2}+c_{V}\rho \theta =0.5\rho {\left|u\right|}^{2}+\rho \varepsilon$. Other choices of sought functions are possible as well.

Next, consider the following formal regularization [11], also for the reader’s convenience. In the Euler balance PDEs for the mass of components (A1), we replace

(A8) $$\begin{array}{c} \rho_{\alpha }u\;\to \;\rho_{\alpha }u+\tau \partial_{t}\left(\rho_{\alpha }u\right)=\rho_{\alpha }u+\tau \left(\left(\partial_{t}\rho_{\alpha }\right)u+\rho_{\alpha }\partial_{t}u\right)\\ =\rho_{\alpha }u-\tau \left(\mathrm{div}\left(\rho_{\alpha }u\right)u+\rho_{\alpha }\left(\left(u\cdot \nabla \right)u+\frac{1}{\rho }\nabla p-f\right)\right)=\rho_{\alpha }\left(u-w_{1\alpha }\right), \end{array}$$

where τ>0 is a regularization parameter and the arisen regularizing velocity $w_{1\alpha }$ is given by Formula (9) for ℓ=1. Thus, we come to the regularized balance Equation (1), namely with the regularizing velocity (9), for ℓ=1 and $d_{1}=\ldots =d_{K}=0$.

In the Euler balance equation for the total momentum (A2), we replace

$$\begin{array}{c} \mathrm{div}\left(\rho u⊗u\right)+\nabla p-\rho f\;\to \;\mathrm{div}\left(\rho u⊗u+\tau \partial_{t}\left(\rho u⊗u\right)\right)+\nabla \left(p+\tau \partial_{t}p\right)-\left(\rho +\tau \partial_{t}\rho \right)f\\ =\mathrm{div}\left(\rho u⊗u-\tau (\rho w_{1}⊗u+\rho u⊗\hat{w})\right)\\ +\nabla \left(p-\tau (u\cdot \nabla p+\gamma p\mathrm{div}u-(\gamma -1)Q)\right)-\left(\rho -\tau \mathrm{div}(\rho u)\right)f\\ =\mathrm{div}(\rho \left(u-w_{1}\right)⊗u)+\nabla p-\mathrm{div}{\tilde{\Pi }}^{\tau }-\left(\rho -\tau \mathrm{div}(\rho u)\right)f \end{array}$$

since $\partial_{t}\left(\rho u⊗u\right)=\partial_{t}\left(\rho u\right)⊗u+\rho u⊗\partial_{t}u$ and then owing to the above Euler PDEs (A2), (A6), (A7) and (A4), sequentially. Here, the averaged regularizing velocity $w_{1}$ is given by Formula (8) for ℓ=1, and the regularizing viscous stress is defined by

$${\tilde{\Pi }}^{\tau }:=\rho u⊗\hat{w}+\tau \left(u\cdot \nabla p+\gamma p\mathrm{div}u-(\gamma -1)Q\right)$$

which in the last two terms differs from $\Pi_{1}^{\tau }$ given by (11) for ℓ=1 (except for the simple case $\gamma_{1}=\ldots =\gamma_{K}$).

In the Euler balance equation for the total energy of the mixture (A3), we first replace

$$\left(E+p\right)u\;\to \;\left(E+p\right)u+\tau \partial_{t}\left((E+p)u\right)=\left(E+p\right)u+\tau \left(\left(\partial_{t}E+\partial_{t}p\right)u+\left(E+p\right)\partial_{t}u\right).$$

We accomplish the following transformations

$$\begin{array}{c} \partial_{t}E=-\mathrm{div}\left((E+p)u\right)+\rho u\cdot f+Q=-\nabla \frac{E+p}{\rho }\cdot \rho u-\frac{E+p}{\rho }\mathrm{div}\left(\rho u\right)+\rho u\cdot f+Q\\ =-\left(\left(u\cdot \nabla \right)u+\nabla \varepsilon +\frac{1}{\rho }\nabla p-\frac{p}{\rho^{2}}\nabla \rho -f\right)\cdot \rho u-\left(E+p\right)\frac{1}{\rho }\mathrm{div}\left(\rho u\right)+Q. \end{array}$$

Consequently, we derive

$$\begin{array}{c} \left(E+p\right)u+\tau \partial_{t}\left((E+p)u\right)=\left(E+p\right)\left(u-\tau \left(\frac{1}{\rho }\mathrm{div}\left(\rho u\right)u-\partial_{t}u\right)\right)\\ -\tau \left(\left(\rho \nabla \varepsilon -\frac{p}{\rho }\nabla \rho \right)\cdot u-Q\right)u-\left(\hat{w}\cdot \rho u-\partial_{t}p\right)\cdot u=\left(E+p\right)\left(u-w_{1}\right)+{\tilde{q}}^{\tau }-{\tilde{\Pi }}^{\tau }u, \end{array}$$

with a regularizing heat flux

$$-{\tilde{q}}^{\tau }:=\tau \left((\rho \nabla \varepsilon -R\theta \nabla \rho )\cdot u-Q\right)u,$$

which in the first and second terms essentially differs from $-q^{\tau }$ given by (12) (including even the particular cases $c_{V1}=\ldots =c_{VK}$ and $R_{1}=\ldots =R_{K}$). Second, we also replace

$$\begin{array}{c} \rho u\cdot f\;\to \;\left(\rho u+\tau \partial_{t}\left(\rho u\right)\right)\cdot f=\rho \left(u-w_{1}\right)\cdot f \end{array}$$

owing to the Euler balance equation for the total momentum (A2).

All these replacements together lead from the original Euler-type system (A1)–(A3) to its following regularized version

$$\begin{array}{c} \partial_{t}\rho_{\alpha }+\mathrm{div}\left(\rho_{\alpha }\left(u-w_{1\alpha }\right)\right)=0,\;\;\alpha =\bar{1,K},\\ \partial_{t}\left(\rho u\right)+\mathrm{div}\left(\rho \left(u-w_{1}\right)⊗u\right)+\nabla p=\mathrm{div}{\tilde{\Pi }}^{\tau }+\left(\rho -\tau \mathrm{div}(\rho u)\right)f,\\ \partial_{t}E+\mathrm{div}\left(\left(E+p\right)\left(u-w_{1}\right)\right)=\mathrm{div}\left(-{\tilde{q}}^{\tau }+{\tilde{\Pi }}^{\tau }u\right)+\rho \left(u-w_{1}\right)\cdot f+Q. \end{array}$$

Notice that not only the terms ${\tilde{\Pi }}^{\tau }$ and ${\tilde{q}}^{\tau }$ but also the term $\mathrm{div}\left(\left(E+p\right)\left(u-w_{1}\right)\right)$ are different from their respective counterparts in Equations (2) and (3). One can also add the above Navier–Stokes–Fourier terms $\Pi^{NS}$ and $q^{F}$, see (10), to ${\tilde{\Pi }}^{\tau }$ and ${\tilde{q}}^{\tau }$ and thus obtain the regularized QGD-type system. Such a system, where $w_{1\alpha }$ is replaced with $w_{1}$, has recently been applied in [39] (without any its derivation, only by the formal analogy to the single-component gas case) and, in a sense, looks closer to the standard form of the corresponding single-component gas system of Equations (23)–(25), than the regularized system (1)–(3) from Section 2, though both the systems for mixtures are reduced to it in the case of a single component. Recall that the derivation of system (1)–(3) was accomplished in a quite different way by aggregating the regularized multi-velocity and multi-temperature gas mixture equations [30].

However, a significant theoretical drawback of the system derived in this Appendix and used in practice, with the regularizing velocity either $w_{1\alpha }$, or $w_{1}$ instead, is the failure of attempts to prove the non-negativity of the entropy production for it, in contrast to the regularized systems considered in [34,35,37] and system (1)–(3) with the combined regularizing velocity (9). The reason of this drawback is any of the above mentioned differences in the regularized terms.

## Appendix B

In this appendix, we accomplish the scaling of the regularized system of PDEs from Section 2 that is used to solve many problems. Define the scaled sought functions and variables

$${\bar{\rho }}_{\alpha }=\frac{\rho_{\alpha }}{\rho_{*}},\;\;\bar{u}=\frac{u}{u_{*}},\;\;\bar{\theta }=\frac{\theta }{\theta_{*}},\;\;\bar{x}=\frac{x}{x_{*}},\;\;\bar{t}=\frac{t}{t_{*}}$$

with arbitrary scaling constant parameters $\rho_{*}>0$, $u_{*}>0$, $\theta_{*}>0$ and $x_{*}>0$ as well as $t_{*}=\frac{x_{*}}{u_{*}}$.

Inserting $\rho_{\alpha }=\rho_{*}{\bar{\rho }}_{\alpha }$, $u=u_{*}\bar{u}$, $\theta =\theta_{*}\bar{\theta }$, $x=x_{*}\bar{x}$ and $t=t_{*}\bar{t}$ in the main balance PDEs (1), (2) and (3) and multiplying them by $\frac{x_{*}}{\rho_{*}u_{*}}$, $\frac{x_{*}}{\rho_{*}u_{*}^{2}}$ and $\frac{x_{*}}{\rho_{*}u_{*}^{3}}$, respectively, we find that the PDEs do not change their form for the scaled sought functions:

$$\begin{array}{c} \partial_{\bar{t}}{\bar{\rho }}_{\alpha }+{\mathrm{div}}_{\bar{x}}\left({\bar{\rho }}_{\alpha }\left(\bar{u}-{\bar{w}}_{\ell \alpha }\right)+{\bar{d}}_{\alpha }\right)=0,\;\;\alpha =\bar{1,K},\\ \partial_{\bar{t}}\left(\bar{\rho }\bar{u}\right)+{\mathrm{div}}_{\bar{x}}\left(\bar{\rho }\left(\bar{u}-{\bar{w}}_{\ell }\right)⊗\bar{u}\right)+\nabla_{\bar{x}}\bar{p}={\mathrm{div}}_{\bar{x}}\bar{\Pi }+\left(\bar{\rho }-\ell \tau {\mathrm{div}}_{\bar{x}}\left(\bar{\rho }\bar{u}\right)\right)\bar{f},\\ \partial_{\bar{t}}\bar{E}+{\mathrm{div}}_{\bar{x}}\left(0.5\bar{\rho }{\left|\bar{u}\right|}^{2}\left(\bar{u}-{\bar{w}}_{\ell }\right)+\langle {\bar{\rho }}_{\alpha }{\bar{h}}_{\alpha }\left(\bar{u}-{\bar{w}}_{\ell \alpha }\right)\rangle \right)={\mathrm{div}}_{\bar{x}}\left(-\bar{q}+\bar{\Pi }\bar{u}\right)+\bar{\rho }\left(\bar{u}-{\bar{w}}_{\ell }\right)\cdot \bar{f}+\bar{Q} \end{array}$$

for $\left(\bar{x},\bar{t}\right)\in \tilde{\Omega }\times \left[0,\bar{T}\right]$, where $\tilde{\Omega }:=\Omega /x_{*}$, i.e., $\bar{x}\in \tilde{\Omega }\Leftrightarrow x_{*}\bar{x}\in \Omega$, and $\bar{T}=\frac{T}{t_{*}}$. Here, the following derivatives and differential operators are involved $\partial_{\bar{t}}=\frac{\partial }{\partial \bar{t}}$, $\partial_{{\bar{x}}_{i}}=\frac{\partial }{\partial {\bar{x}}_{i}}$, ${\mathrm{div}}_{\bar{x}}=\nabla_{\bar{x}}\cdot$ and $\nabla_{\bar{x}}=\left(\partial_{{\bar{x}}_{1}},\ldots ,\partial_{{\bar{x}}_{n}}\right)$; also $\bar{f}=\frac{x_{*}f}{u_{*}^{2}}$, ${\bar{Q}}_{\alpha }=\frac{x_{*}Q_{\alpha }}{\rho_{*}u_{*}^{3}}$ and $\bar{Q}=\langle {\bar{Q}}_{\alpha }\rangle$.

The scaled density, specific internal energy, total energy and specific enthalpy of the components conserve their form

$$\begin{array}{c} {\bar{p}}_{\alpha }=\left(\gamma_{\alpha }-1\right){\bar{\rho }}_{\alpha }{\bar{\varepsilon }}_{\alpha }={\bar{R}}_{\alpha }{\bar{\rho }}_{\alpha }\bar{\theta },\;\;{\bar{\varepsilon }}_{\alpha }={\bar{c}}_{V\alpha }\bar{\theta },\;\;{\bar{E}}_{\alpha }=0.5{\bar{\rho }}_{\alpha }{\left|\bar{u}\right|}^{2}+{\bar{\rho }}_{\alpha }{\bar{\varepsilon }}_{\alpha },\;\;{\bar{h}}_{\alpha }={\bar{\varepsilon }}_{\alpha }+\frac{{\bar{p}}_{\alpha }}{{\bar{\rho }}_{\alpha }}={\bar{c}}_{p\alpha }\bar{\theta }, \end{array}$$

with the same $\gamma_{\alpha }$ but the other constants scaled:

$$\begin{array}{c} \gamma_{\alpha }-1=\frac{{\bar{R}}_{\alpha }}{{\bar{c}}_{V\alpha }}=\frac{R_{\alpha }}{c_{V\alpha }},\;\;{\bar{R}}_{\alpha }=\frac{\theta_{*}R_{\alpha }}{u_{*}^{2}},\;\;{\bar{c}}_{V\alpha }=\frac{\theta_{*}c_{V\alpha }}{u_{*}^{2}},\;\;{\bar{c}}_{p\alpha }=\frac{\theta_{*}c_{p\alpha }}{u_{*}^{2}},\;\;\alpha =\bar{1,K}. \end{array}$$

The scaled total density and pressure, average specific internal energy and total energy of the mixture also conserve their form

$$\begin{array}{c} \;\bar{\rho }=\langle {\bar{\rho }}_{\alpha }\rangle ,\;\bar{p}=\langle {\bar{p}}_{\alpha }\rangle =\bar{R}\bar{\rho }\bar{\theta }=\left(\gamma -1\right)\bar{\rho }\bar{\varepsilon },\;\bar{\varepsilon }=\langle \frac{{\bar{\rho }}_{\alpha }}{\bar{\rho }}{\bar{\varepsilon }}_{\alpha }\rangle ={\bar{c}}_{V}\bar{\theta },\;\bar{E}=\langle {\bar{E}}_{\alpha }\rangle =0.5\bar{\rho }{\left|\bar{u}\right|}^{2}+\bar{\rho }\bar{\varepsilon }, \end{array}$$

with the same γ but the scaled $\bar{R}$ and ${\bar{c}}_{V}$:

$$\begin{array}{c} \gamma -1=\frac{\bar{R}}{{\bar{c}}_{V}}=\frac{R}{c_{V}},\;\;\bar{R}:=\langle \frac{{\bar{\rho }}_{\alpha }}{\bar{\rho }}{\bar{R}}_{\alpha }\rangle =\frac{\theta_{*}R}{u_{*}^{2}},\;\;{\bar{c}}_{V}:=\langle \frac{{\bar{\rho }}_{\alpha }}{\bar{\rho }}{\bar{c}}_{V\alpha }\rangle =\frac{\theta_{*}c_{V}}{u_{*}^{2}}. \end{array}$$

The scaled regularizing velocities together with the viscosity tensor and heat flux are expressed in a natural way

$$\begin{array}{c} {\bar{w}}_{\ell \alpha }=\frac{\bar{\tau }}{{\bar{\rho }}_{\alpha }}{\mathrm{div}}_{\bar{x}}\left({\bar{\rho }}_{\alpha }\bar{u}\right)\bar{u}+\ell \bar{\hat{w}},\;\;\bar{\hat{w}}=\bar{\tau }\left(\left(\bar{u}\cdot \nabla_{\bar{x}}\right)\bar{u}+\frac{1}{\bar{\rho }}\nabla_{\bar{x}}\bar{p}-\bar{f}\right),\;\;\\ {\bar{w}}_{\ell }:=\langle \frac{{\bar{\rho }}_{\alpha }}{\bar{\rho }}{\bar{w}}_{\ell \alpha }\rangle =\ell \frac{\bar{\tau }}{\bar{\rho }}{\mathrm{div}}_{\bar{x}}\left(\bar{\rho }\bar{u}\right)\bar{u}+\bar{\hat{w}},\\ \bar{\Pi }={\bar{\Pi }}^{NS}+{\bar{\Pi }}_{\ell }^{\tau },\;\;\bar{q}={\bar{q}}^{F}+{\bar{q}}^{d}+\ell {\bar{q}}^{\tau },\\ {\bar{\Pi }}^{NS}=\bar{\mu }\left(\nabla_{\bar{x}}\bar{u}+{\left(\nabla_{\bar{x}}\bar{u}\right)}^{T}\right)+\left(\bar{\lambda }-\frac{2}{3}\bar{\mu }\right)\left({\mathrm{div}}_{\bar{x}}\bar{u}\right)I,\;\;-{\bar{q}}^{F}=\bar{\varkappa }\nabla_{\bar{x}}\bar{\theta },\\ {\bar{\Pi }}_{\ell }^{\tau }=\bar{\rho }\bar{u}⊗\bar{\hat{w}}+\ell \bar{\tau }\left(\bar{u}\cdot \nabla_{\bar{x}}\bar{p}+\langle \gamma_{\alpha }{\bar{p}}_{\alpha }\rangle {\mathrm{div}}_{\bar{x}}\bar{u}-\langle \gamma_{\alpha }{\bar{Q}}_{\alpha }\rangle +\bar{Q}\right)I,\\ -{\bar{q}}^{\tau }=\bar{\tau }\left\{\left({\bar{c}}_{V}\bar{\rho }\nabla_{\bar{x}}\bar{\theta }-\bar{\theta }\nabla_{\bar{x}}\left(\bar{R}\bar{\rho }\right)\right)\cdot \bar{u}-\bar{Q}\right\}\bar{u}, \end{array}$$

but with the following scaled coefficients

$$\bar{\tau }=\frac{u_{*}\tau }{x_{*}},\;\;\bar{\mu }=\frac{\mu }{\rho_{*}u_{*}x_{*}},\;\;\bar{\lambda }=\frac{\lambda }{\rho_{*}u_{*}x_{*}},\;\;\bar{\varkappa }=\frac{\theta_{*}\varkappa }{\rho_{*}u_{*}^{3}x_{*}}.$$

In particular, in the case of τ and the artificial coefficients given by expressions (19) or (2), they conserve their form

$$\bar{\tau }=\frac{a\bar{h}}{{\bar{c}}_{s}+i_{\tau }\left|\bar{u}\right|},\;\;\bar{\mu }=\bar{\tau }\langle a_{S\alpha }{\bar{p}}_{\alpha }\rangle ,\;\;\bar{\lambda }=\bar{\tau }\langle a_{1S\alpha }{\bar{p}}_{\alpha }\rangle ,\;\;\bar{\varkappa }=\bar{\tau }\langle a_{Pr\alpha }\gamma_{\alpha }{\bar{c}}_{V\alpha }{\bar{p}}_{\alpha }\rangle ,$$

with $\bar{h}:=\frac{h}{x_{*}}$ and ${\bar{c}}_{s}=\sqrt{\gamma \left(\gamma -1\right)\bar{\varepsilon }}=\sqrt{\gamma \bar{R}\bar{\theta }}$, or $\bar{\mu }=\alpha_{S}\bar{\tau }\bar{p}$, $\bar{\lambda }=\alpha_{1S}\bar{\tau }\bar{p}$ and $\bar{\varkappa }=\alpha_{Pr}\gamma {\bar{c}}_{V}\bar{\mu }$.

The diffusion fluxes and additional respective heat flux take the form

$$\begin{array}{c} -{\bar{d}}_{\alpha }:={\langle {\bar{d}}_{\alpha \beta }\left(\nabla_{\bar{x}}\left({\bar{G}}_{\alpha }-{\bar{G}}_{\beta }\right)+\left({\bar{e}}_{\alpha }-{\bar{e}}_{\beta }\right)\nabla_{\bar{x}}\bar{\theta }\right)\rangle }_{\beta },\;\;{\bar{q}}^{d}=\langle \left({\bar{G}}_{\alpha }+{\bar{e}}_{\alpha }\bar{\theta }\right){\bar{d}}_{\alpha }\rangle ,\\ {\bar{G}}_{\alpha }={\bar{\varepsilon }}_{\alpha }-{\bar{s}}_{\alpha }\bar{\theta }+\frac{{\bar{p}}_{\alpha }}{{\bar{\rho }}_{\alpha }}=\left({\bar{c}}_{p\alpha }-{\bar{s}}_{\alpha }\right)\bar{\theta },\;\;{\bar{s}}_{\alpha }={\bar{s}}_{\alpha 0}-{\bar{R}}_{\alpha }\ln{\bar{\rho }}_{\alpha }+{\bar{c}}_{V\alpha }\ln\bar{\theta }, \end{array}$$

with the scaled coefficients

$${\bar{d}}_{\alpha \beta }=\frac{u_{*}d_{\alpha \beta }}{\rho_{*}x_{*}},\;\;{\bar{e}}_{\alpha }=\frac{\theta_{*}e_{\alpha }}{u_{*}^{2}},\;\;{\bar{s}}_{\alpha 0}=\frac{\theta_{*}s_{\alpha 0}}{u_{*}^{2}},\;\;\rho_{\alpha 0}=\rho_{*},\;\;\theta_{0}=\theta_{*}.$$

## Appendix C

We consider the 1D version of the Euler-type system of PDEs (A1)–(A3) considered in Appendix A. Then, for discontinuous solutions, we consider the Rankine–Hugoniot conditions on the line of discontinuity x=ξ(t) of the values of the sought functions. Quite similarly, for example, to ([60], Section 2.4) and taking into account Formula (5) for ρ, *p*, ε and *E*, these conditions have the form

(A9) $$\begin{array}{c} \xi^{\prime }{\left[\rho_{\alpha }\right]}_{\xi }={\left[\rho_{\alpha }u\right]}_{\xi },\;\;\alpha =\bar{1,K},\;\;\xi^{\prime }{\left[\rho u\right]}_{\xi }={\left[\rho u^{2}+p\right]}_{\xi },\;\;\xi^{\prime }{\left[E\right]}_{\xi }={\left[\left(E+p\right)u\right]}_{\xi }; \end{array}$$

in this appendix, ${\left[\varphi \right]}_{\xi }\left(t\right):=\varphi \left(\xi \left(t\right)+0,t\right)-\varphi \left(\xi \left(t\right)-0,t\right)$ is the jump in the values of a function φ through the specified line of discontinuity. It is also assumed that the limit values of all the sought functions to the left and to the right of the discontinuity are not identical, otherwise there is actually no solution discontinuity.

In the literature, the so-called *stationary shock waves* are known for which ξ(t)=const. Let $\psi_{l}:=\psi \left(\xi -0\right)$ and $\psi_{r}:=\psi \left(\xi +0\right)$ be the limit values from the left and from the right at the point x=ξ of a function ψ(x).

 **Proposition A1.**
*For the existence of the stationary shock wave, the following conditions should be valid*

(A10)
$$\begin{array}{c} M_{l}^{2}:=\frac{u_{l}^{2}}{\gamma \left(\gamma -1\right)\varepsilon_{l}}>\frac{\gamma -1}{2\gamma }\;\;\mathrm{with}\;\;\gamma =\gamma_{l}=\frac{R_{l}}{c_{Vl}}+1,\;\;M_{l}^{2}\neq 1. \end{array}$$

*Herewith $\gamma_{r}=\gamma_{l}$, and the values of the functions to the right of the discontinuity can be expressed in terms of their values to the left of it with the help of the relations*

(A11)
$$\begin{array}{c} \frac{\rho_{\alpha l}}{\rho_{\alpha r}}=\frac{u_{r}}{u_{l}}=a:=\frac{2+\left(\gamma -1\right)M_{l}^{2}}{\left(\gamma +1\right)M_{l}^{2}},\;\;\alpha =\bar{1,K},\;\;\frac{p_{r}}{p_{l}}=b:=\frac{2\gamma M_{l}^{2}-\left(\gamma -1\right)}{\gamma +1},\;\;\frac{\theta_{r}}{\theta_{l}}=ab. \end{array}$$

 **Proof.** For $\xi^{\prime }=0$, conditions (A9) lead to the system of nonlinear algebraic equations

(A12) $$\begin{array}{c} \rho_{\alpha r}u_{r}=\rho_{\alpha l}u_{l},\;\;\alpha =\bar{1,K},\;\;\rho_{r}u_{r}^{2}+p_{r}=\rho_{l}u_{l}^{2}+p_{l}, \end{array}$$

(A13) $$\begin{array}{c} \left(0.5\rho_{r}u_{r}^{2}+\rho_{r}\varepsilon_{r}+p_{r}\right)u_{r}=\left(0.5\rho_{l}u_{l}^{2}+\rho_{l}\varepsilon_{l}+p_{l}\right)u_{l}. \end{array}$$

By virtue of the first of them, $u_{l}\neq 0$, and we sequentially obtain

(A14) $$\begin{array}{c} \rho_{\alpha r}=a^{-1}\rho_{\alpha l},\;\;\alpha =\bar{1,K},\;\;a:=\frac{u_{r}}{u_{l}}, \end{array}$$

(A15) $$\begin{array}{c} \rho_{r}=a^{-1}\rho_{l},\;\;R_{r}=R_{l},\;\;c_{Vr}=c_{Vl},\;\;\gamma_{r}=\gamma_{l},\;\;\rho_{r}u_{r}=\rho_{l}u_{l}. \end{array}$$

Then $\rho_{r}u_{r}^{2}=a\rho_{l}u_{l}^{2}$, and by virtue of the second Equation (A12) we obtain $p_{r}=p_{l}+\rho_{l}u_{l}^{2}\left(1-a\right)$. Now, we rewrite Equation (A13), after division by $u_{l}$ and due to the formula $\rho \varepsilon =\frac{p}{\gamma -1}$, in the form

$$\left\{0.5a\rho_{l}u_{l}^{2}+\frac{\gamma }{\gamma -1}\left(p_{l}+\rho_{l}u_{l}^{2}\left(1-a\right)\right)\right\}a=0.5\rho_{l}u_{l}^{2}+\frac{\gamma }{\gamma -1}p_{l}.$$

We divide it by $\gamma p_{l}$, apply the formula $\frac{\rho u^{2}}{\gamma p}=\frac{u^{2}}{\gamma (\gamma -1)\varepsilon }$ and obtain the quadratic equation for *a*:

$$\left(\frac{\gamma }{\gamma -1}-0.5\right)M_{l}^{2}a^{2}-\frac{1}{\gamma -1}\left(1+\gamma M_{l}^{2}\right)a+0.5M_{l}^{2}+\frac{1}{\gamma -1}=0.$$

Its trivial root a=1 corresponds to the coincidence of the right and left values of the sought functions. Therefore, the nontrivial root is $a=\frac{2+\left(\gamma -1\right)M_{l}^{2}}{\left(\gamma +1\right)M_{l}^{2}}\neq 1$ for $M_{l}^{2}\neq 1$, that according to equalities (A14) leads to the first Formula (A11). Substituting it in the formula $p_{r}=p_{l}+\rho_{l}u_{l}^{2}\left(1-a\right)$ leads to the expression for $\frac{p_{r}}{p_{l}}$ specified in Formula (A11), which is correct (gives the value $p_{r}>0$) under the first condition (A10). The last formula (A11) follows from the formulas $\theta =\frac{p}{R\rho }$ and (A15). □

This proposition should be used to state more precisely the initial data for some numerical experiments such as in ([8], Section 9.8.3). Relations (A11) are well-known for the single-component gas, for example, see ([61], Chapter 4). Obviously $\frac{\gamma -1}{2\gamma }<0.5$. In addition, inequalities a<1 or b>1 are equivalent to $M_{l}^{2}>1$. It is simple to check that the inequality ab>1 is equivalent to

$$\gamma M_{l}^{4}-\left(\gamma -1\right)M_{l}^{2}-1=\gamma \left(M_{l}^{2}-1\right)\left(M_{l}^{2}+\gamma^{-1}\right)>0,$$

i.e., to $M_{l}^{2}>1$ once again. The same is true concerning the inequality $\frac{a}{b}<1$. Consequently, $\frac{1}{b}<a<1<b$ for $M_{l}^{2}>1$, or $\frac{1}{b}>a>1>b$ for $\frac{\gamma -1}{2\gamma }<M_{l}^{2}<1$. Thus, in particular, we have

(A16) $$\begin{array}{c} \rho_{\alpha r}>\rho_{\alpha l},\;\;|u_{r}\left|<\right|u_{l}|,\;\;p_{r}>p_{l},\;\;\theta_{r}>\theta_{l}\;\;\mathrm{for}\;\;M_{l}^{2}>1, \end{array}$$

and the opposite inequalities hold for $\frac{\gamma -1}{2\gamma }<M_{l}^{2}<1$. In addition, it is simple to check that

$$\frac{\gamma -1}{2\gamma }<M_{r}^{2}:=\frac{u_{r}^{2}}{\gamma \left(\gamma -1\right)\varepsilon_{r}}=\frac{a^{2}u_{l}^{2}}{\gamma \left(\gamma -1\right)ab\varepsilon_{l}}=\frac{a}{b}M_{l}^{2}<1$$

for $M_{l}^{2}>1$, or $M_{r}^{2}>1$ for $\frac{\gamma -1}{2\gamma }<M_{l}^{2}<1$. We emphasize that the important stability issue is not touched here.

The expression $M^{2}=\frac{u^{2}}{\gamma (\gamma -1)\varepsilon }$ that has just arisen in relations (A10) is the squared Mach number in mixtures corresponding to the sound speed in mixtures defined as in Section 2. We have considered the stationary shock waves but it is well-known that non-stationary ones can be reduced to them by passing to a moving system of coordinates [60].

## Appendix D

We present the 1D finite-difference counterpart of Proposition 2. Let $\delta_{t}y^{m}=\frac{y^{m+1}-y^{m}}{h_{t(m+1)}}$.

 **Proposition A2.**
*Let $0<C_{\alpha }<1$, $\alpha =\bar{1,K}$, be arbitrary constants such that $\langle C_{\alpha }\rangle =1$. Consider the sought functions of the particular form $\rho_{\alpha }=C_{\alpha }\rho$ with ρ>0, $\alpha =\bar{1,K}$, u and θ>0, to the finite-difference scheme such as the semi-discrete Equations (63)–(69), but with $\partial_{t}$ replaced with $\delta_{t}$ and [0,T] replaced with the mesh $\left\{t_{0},\ldots ,t_{\bar{m}-1}\right\}$, in the case of $d_{\alpha }=0$, f=0 and, for example, τ=[T(ρ,ε,u)]⩾0, ν=[N(ρ,ε,u)]⩾0 and ϰ=[K(ρ,ε,u)]⩾0 in space on $\omega_{h}^{*}$. For them, this finite-difference scheme is reduced to the following one for the regularized system of PDEs for a single-component gas dynamics*

(A17)
$$\begin{array}{c} \delta_{t}\rho +\delta^{*}\left({\left[\rho \right]}_{\ln}\left(\left[u\right]-w_{\ell }\right)\right)=0, \end{array}$$

(A18)
$$\begin{array}{c} \delta_{t}\left(\rho u\right)+\delta^{*}\left({\left[\rho \right]}_{\ln}\left(\left[u\right]-w_{\ell }\right)\left[u\right]+\left[p\right]\right)=\delta^{*}\Pi_{\ell }, \end{array}$$

(A19)
$$\begin{array}{c} \delta_{t}E+\delta^{*}\left\{\left({\left[E\right]}_{2}+\left[p\right]\right)\left(\left[u\right]-w_{\ell }\right)-0.25h^{2}\delta u\cdot \delta p\right\}=\delta^{*}\left(-q_{\ell }+\Pi_{\ell }\left[u\right]\right)+{\left[Q\right]}^{*} \end{array}$$

*for the sought functions ρ, u and θ on $\omega_{h}\times \left\{t_{0},\ldots ,t_{\bar{m}-1}\right\}$. Here, the above expressions (26) for ρ, ε, E, R and $c_{V}$ are used with |u|=u together with the formulas $\tilde{\gamma }=\langle C_{\alpha }R_{\alpha }\gamma_{\alpha }\rangle /\langle C_{\alpha }R_{\alpha }\rangle$ and*

$$\begin{array}{c} w_{\ell }=\ell \frac{\tau }{\left[\rho \right]}\;\left[u\right]\delta \left(\rho u\right)+\hat{w},\;\;\hat{w}=\frac{\tau }{\left[\rho \right]}\;\left(\left[\rho \right]\left[u\right]\delta u+\delta p\right), \end{array}$$

(A20)
$$\begin{array}{c} \Pi_{\ell }=\nu \delta u+\Pi_{\ell }^{\tau },\;\;\Pi_{\ell }^{\tau }=\left[u\right]\left[\rho \right]\hat{w}+\ell \tau \{\left[u\right]\delta p+\tilde{\gamma }{\left[p\right]}_{1}\delta u-\left(\gamma_{1}-1\right)Q\}, \end{array}$$

(A21)
$$\begin{array}{c} q_{\ell }=-\varkappa \delta \theta +\ell \tau \left\{\left(c_{V}\left[\rho \right]\delta \theta -R\left[\theta \right]\delta \rho \right){\left[u\right]}^{2}-Q\left[u\right]\right\}. \end{array}$$

 **Proof.** For $\rho_{\alpha }=C_{\alpha }\rho$, under the assumptions made about $C_{\alpha }$, we obtain

$$\begin{array}{c} {\left[C_{\alpha }\rho \right]}_{\ln}=C_{\alpha }{\left[\rho \right]}_{\ln},\;\;w_{\ell \alpha }=\ell \frac{\tau }{\left[\rho \right]}\left[u\right]\delta \left(\rho u\right)+\hat{w}=w_{\ell },\;\;\langle \gamma_{\alpha }{\left[p_{\alpha }\right]}_{1}\rangle =\tilde{\gamma }{\left[p\right]}_{1},\\ \langle {\left[E_{\alpha }\right]}_{2}\rangle =\langle 0.5C_{\alpha }{\left[\rho \right]}_{\ln}u_{-}u_{+}+C_{\alpha }{\left[\rho \right]}_{\ln}c_{V\alpha }{\left[\theta \right]}^{\ln}\rangle =0.5{\left[\rho \right]}_{\ln}u_{-}u_{+}+{\left[\rho \right]}_{\ln}\langle c_{V\alpha }C_{\alpha }\rangle {\left[\theta \right]}^{\ln}={\left[E\right]}_{2},\\ \langle c_{V\alpha }\left[\rho_{\alpha }\right]\rangle =\langle c_{V\alpha }C_{\alpha }\rangle \left[\rho \right]=c_{V}\rho ,\;\;\langle R_{\alpha }\rho_{\alpha }\rangle =\langle R_{\alpha }C_{\alpha }\rangle \rho =R\rho . \end{array}$$

Consequently, the discrete balance equations for the mass of components such as (63), with $\partial_{t}$ replaced with $\delta_{t}$, are reduced to Equation (A17). In addition, expressions (67) and (68) for $\Pi_{\ell }^{\tau }$ and $-q^{\tau }$ are reduced to those given in Formulas (A20) and (A21). Therefore the discrete balance equations for the total momentum and total energy such as (64) and (65), with $\partial_{t}$ replaced with $\delta_{t}$, take forms (A18) and (A19). □

Proposition A2 is useful to check some properties of solutions to the constructed finite-difference scheme for the gas mixture dynamics and also to test codes that implement the scheme.
